# Task-specific theta enhancement and domain-general alpha/beta suppression as oscillatory signatures of cognitive flexibility

**DOI:** 10.1038/s44271-026-00521-9

**Published:** 2026-10-15

**Authors:** Mareike Hülsemann, Christoph Löffler, Anna-Lena Schubert

**Affiliations:** Department of Psychology, Johannes Gutenberg University Mainz, Mainz, Germany

**Keywords:** Cognitive control, Intelligence

## Abstract

Cognitive flexibility–the ability to adapt to changing situational or task demands–is a fundamental aspect of human cognition and a potential source of individual differences in higher-order cognitive abilities. To further understand the neural mechanisms underlying this ability, we examined event-related spectral perturbations (ERSP) in three cued switching tasks (parity/magnitude, global/local, number/letter) in a sample of 148 adults. Employing a data-driven approach that combined mass-univariate time-frequency analysis, cluster-based permutation testing, and latent change structural equation modeling, we identified two robust neural signatures: a transient frontal theta increase and a sustained alpha/beta suppression. Both effects were more pronounced in switch compared to repeat trials. Frontal theta reflected task-specific control processes, whereas parietal alpha/beta indexed domain-general attentional processes that persisted from cue onset through target processing–consistent with two-stage models of task switching. Notably, flexibility-related alpha/beta dynamics did not correlate with measures of intelligence or working memory capacity. However, stronger cue-elicited alpha/beta suppression correlated with reduced non-decision-time switch costs, indicating more effective task preparation, underscoring that alpha/beta suppression captures a neurally coherent but behaviorally distinct dimension of cognitive flexibility. These findings provide evidence that alpha/beta suppression constitutes a reliable, cross-task generalizable neural signature of attentional reconfiguration during task switching, while highlighting the multi-component nature of individual differences in cognitive flexibility.

## Abstract (web summary)

Using time-frequency analysis and latent change structural equation modeling on cued-task switching data, we show that parietal alpha/beta suppression indexes cognitive flexibility with stronger suppression indicating more effective task preparation.

## Introduction

Cognitive flexibility is a key element of human behavior that enables us to adapt to changing circumstances—whether switching between different tasks during the working day^1^ or generating creative solutions to novel problems^2^. We define cognitive flexibility as the ability to adapt to changing task demands by switching between mental sets or rules^3^. As a core component of an influential—though contested—executive function model^4,5^, alongside inhibition and updating, cognitive flexibility is theorized to underlie more complex goal-directed processes such as planning and problem-solving. Yet while cognitive flexibility represents a fundamental capacity for adaptive behavior, individuals show marked differences in their flexibility abilities, and understanding these individual differences remains a central challenge in cognitive psychology.

### Individual differences in cognitive flexibility

Individual differences in cognitive flexibility have profound implications across multiple domains of human functioning. They predict meaningful real-world outcomes such as academic achievement^6,7^, problem-solving capabilities^8^, social interactions^9^, and mental health outcomes^10^. Conversely, reduced cognitive flexibility characterizes numerous clinical populations, including individuals with schizophrenia^11^, depression^12^, and autism spectrum disorder^13^. Some researchers propose that individual differences in cognitive flexibility may even explain the positive correlations observed across diverse cognitive tasks, as most complex cognitive operations require some degree of flexible adaptation^14,15^. For example, the process overlap theory^14^ posits that domain-general cognitive processes, such as core executive functions, act as bottlenecks that cause the positive manifold. In contrast, resource-based frameworks^16^ conceptualize core executive functions as limited resources, predicting that individuals with greater resources will perform better. Taken together, many influential theoretical frameworks suggest that flexibility plays an important role in general cognitive abilities.

### Neural mechanisms underlying cognitive flexibility

To understand individual differences in cognitive flexibility, researchers have turned to neural measures that can provide mechanistic insights beyond behavioral performance alone. Cognitive flexibility is typically assessed using a cued task-switching paradigm, where participants must switch between different rule sets based on contextual cues^4,17^. This paradigm reveals cognitive flexibility through comparing switch trials (requiring rule changes) to repeat trials (maintaining the same rule), with flexibility indexed by switch costs in reaction time and accuracy.

Electrophysiological studies using event-related potentials (ERPs) and time-frequency analyses have identified consistent neural signatures of cognitive flexibility. ERP studies show greater centro-parietal positivity during cue processing in switch versus repeat trials, reflecting proactive control processes, and reduced P3b amplitude following target presentation in switch trials, suggesting ongoing reconfiguration demands^18^. In addition, oscillatory analyses reveal robust spectral signatures: enhanced theta power increases and alpha power suppression are consistently observed in switch compared to repeat conditions^19–22^. Frontal theta enhancement reflects active cognitive control and working memory recruitment^23,24^, while parietal alpha suppression indicates disengagement from task-irrelevant information^25,26^. Crucially, these neural signatures show stability over time^27^ and scale with task difficulty^28^, suggesting they reflect meaningful individual differences in cognitive control capacity.

This dataset has already been used to investigate several cognitive functions. Schubert et al.^29^ showed that individual differences in attentional control can be captured with a neurocognitive psychometric approach, whereas Schubert et al.^30^ demonstrated that neural processing-speed measures robustly predict general cognitive ability. Critically, recent evidence from the dataset used in the present analyses suggests that traditional behavioral indices and the amplitudes of ERP components fail to capture executive function abilities, but merely reflect processing speed^5,31^. However, the failure of established indicators to isolate executive functions using traditional tasks does not preclude measuring them via other indicators within those tasks. For example, Schubert et al.^32^ found in the same dataset that mid-frontal global theta connectivity is a stable, trait-like neural signature of cognitive control that strongly correlates with general cognitive ability. Therefore, in the present analyses, we further explore, whether oscillatory dynamics can serve as measures of individual differences in flexibility. In particular, we focus on measuring flexibility in isolation from basic cognitive processes using latent change modeling.

### Critical knowledge gap: neural correlates of individual differences

Despite this rich understanding of both behavioral individual differences and neural mechanisms at the group level, a fundamental disconnect remains: we do not yet understand whether individual variation in neural signatures like theta enhancement and alpha suppression accounts for the behavioral variability observed across participants. Although experimental work shows that oscillatory dynamics, such as theta power, are causally involved in flexible rule-switching^33^ and can predict individual differences in goal-directed behavior (e.g., response conflict)^34^, the extent to which they reliably index trait-level cognitive flexibility has not yet been demonstrated. This represents a critical gap in our mechanistic understanding of cognitive flexibility. Moving beyond group-average neural responses to examine individual differences in these neural markers is essential for developing a precise understanding of how brain oscillations support flexible cognition^35^.

However, investigating neural correlates of individual differences requires establishing that neural measures meet basic psychometric standards. Currently, the reliability of neural flexibility measures has received insufficient attention in cognitive neuroscience^36^, despite reliability being a prerequisite for valid individual differences research^37^. Without reliable neural measures, variations in brain signals cannot be confidently attributed to genuine individual differences rather than measurement noise. Furthermore, for neural measures to serve as meaningful individual difference variables, they must demonstrate convergent validity across different flexibility tasks. Moreover, they should demonstrate convergent validity across different flexibility measures, such as process-based behavioral measures. Lastly, they should show criterion validity through associations with relevant psychological constructs such as working memory capacity (WMC) and fluid intelligence.

Addressing this knowledge gap requires overcoming several methodological limitations that have constrained previous research on neural mechanisms of cognitive flexibility. First, some studies use simultaneous cue-target presentation or very short cue-target intervals, which conflate endogenous preparation processes with exogenous implementation processes^38,39^. Understanding individual differences requires separating these temporally distinct control processes, as individuals may vary in their capacity for endogenous versus exogenous control strategies.

Second, many previous studies have focused on predefined frequency bands and brain regions, typically examining theta and alpha activity over frontal and parietal regions^19,40^. While this approach has identified important effects, it may miss other frequency bands or brain regions that contribute to individual differences in flexibility. A data-driven mass-univariate approach with cluster-based permutation testing^41^ offers greater statistical power^42^ and enables assessment of effect specificity across the full time-frequency-space domain.

Third, most research examines single tasks, despite evidence that substantial variance in flexibility measures reflects task-specific rather than general flexibility processes^5^. Individual differences in general cognitive flexibility can only be validly assessed by extracting common variance across multiple tasks, mathematically eliminating task-specific influences such as processing speed, semantic proficiency, or stimulus-specific effects.

Finally, previous studies often used modest sample sizes, which preclude robust analyses of individual differences and limit the reliability of neural measures. Adequate statistical power is essential for detecting individual differences in neural oscillations and for implementing advanced analytical approaches such as structural equation modeling.

### The present study

The present study addresses these methodological challenges to provide a comprehensive investigation of individual differences in neural correlates of cognitive flexibility. We employed three distinct switching tasks with relatively long cue-target intervals (800–1200 ms) to distinguish endogenous and exogenous control processes. Using a data-driven time-frequency approach across the full scalp, we identified neural signatures of flexibility and assessed their reliability and validity as individual difference measures. Most importantly, by examining common variance across multiple flexibility tasks in a large sample (*N* = 148), we isolated neural correlates of general cognitive flexibility while eliminating task-specific influences. Additionally, we explore the factor structure underlying behavioral flexibility measures and integrate those measures with the neural measures to support and expand interpretability of those findings.

This methodologically rigorous approach enables us to answer fundamental questions about the neural basis of individual differences in cognitive flexibility: Are these neural individual differences reliable across time and valid across tasks? And do neural flexibility measures relate to other cognitive abilities in theoretically meaningful ways? By bridging the gap between neural mechanisms and individual differences research, this study has the potential to advance both theoretical models of executive functioning and applied research on assessment of cognitive control skills.

## Methods

### Sample

The final sample consisted of 148 participants from the general population (96 females, 51 males, one person declared no affiliation to either gender). The median age of participants was 24 years, with an age range of 18 to 60 years (*M* = 31.5, SD = 13.9). All participants were fluent in German, with all but four being native speakers. Thirteen participants stated to be left-handed. A total of 38.5% of the sample had obtained a university degree, while 58.8% had received a high school diploma as their highest qualification. The majority of the sample (58.8%) was currently enrolled in a university program. The sample was recruited via advertisements in local newspapers, flyers, and via the departmental participant pool at Heidelberg University. Initially, 151 people were invited to participate in the study. However, three participants subsequently withdrew from the study. Participants, who suffered from claustrophobia, were undergoing psychiatric or neurological treatment, had psychological or neurological disorders, were taking medication that affected the nervous system, had skin allergies, had a pacemaker, were color-blind, were obese, or had chronic back pain, were excluded. Participants provided written informed consent prior to participation. For participation, they received monetary compensation and personalized feedback on their performance on intelligence and working memory tests. The study was approved by the local ethics committee (reference number: Löf 2019/1–3). Gender was assessed via self-report. Data on socioeconomic status, communities of descent, and ethnicity were not collected.

### Openness and transparency

This data set is available via OSF^43^ (osf.io/4pvz3) and was initially described in Löffler et al. ^5^. This comprehensive dataset has been analyzed and reported on in previous publications^29–32,44–47^. Access to the pre-processed data and analysis code used in this paper is provided via OSF^48^ (osf.io/aqeph) or via Github (https://github.com/mhuelsemann/flexibility_TFA). The study and current analyses were not pre-registered.

### Materials

All computer-based tasks were programmed in MATLAB 2018b (The MathWorks Inc., Natick, Massachusetts) using Psychtoolbox version 3.0.13^49^. They were presented on a 21.3 in. LCD monitor (Eizo FlexScan S2100) with a 1280 × 1024 resolution and a 60 Hz refresh rate. Participants had a distance of approximately 100 cm to the computer screen.

#### Measurement of flexibility

We used the well-validated and widely used task-cueing paradigm to investigate cognitive flexibility^39^. We implemented three different shifting tasks, which required participants to switch between two categorization tasks indicated by a cue. In each trial, participants had to classify the target according to the cue. The color of the cue (fixation cross in red or green) indicated which rule-set to use. The cue was always valid, such that the cue and target had the same color throughout all trials.

**Fig. 1:**
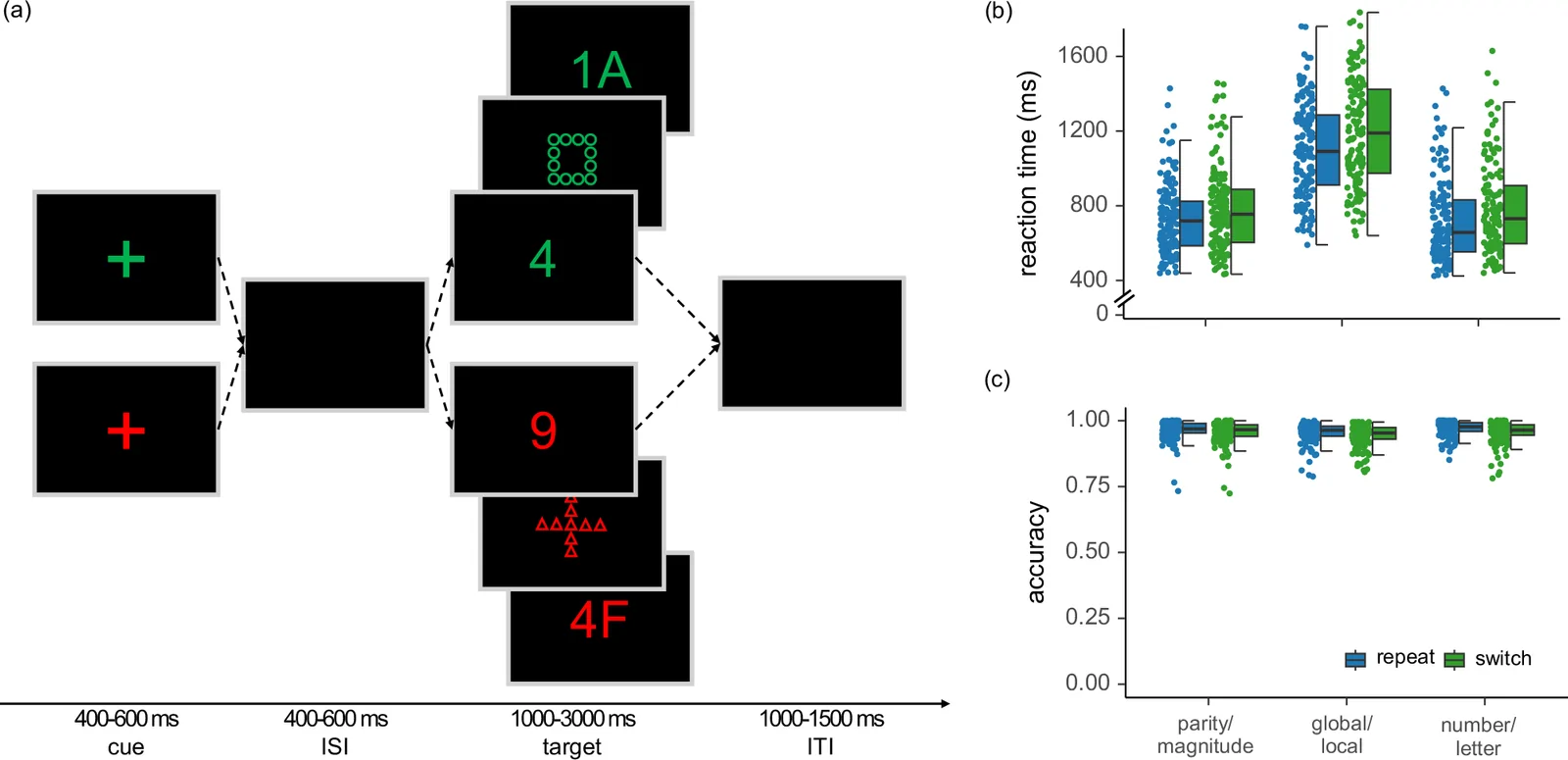
A schematic illustration of a trial of the cued task-switching paradigm and corresponding reaction times and accuracies. **a** Each trial starts with a colored fixation cross, cueing the subsequent rule-set. After a variable inter-stimulus interval, the target was presented in the same color as the cue. Participants had to respond according to the correct rule-set by a key press. Exemplary targets for each of the three shifting tasks are illustrated (from inner to outer: parity/magnitude, global/local, number/letter). Raw data and boxplots of (**b**) reaction times (ms) and **c** accuracy (%) for all three shifting tasks for both conditions are depicted. The boxplot shows the mean (center line), upper and lower quartiles (box limits), and the 95% confidence interval (whiskers). Sample size is *n* = 142 for parity/magnitude task, *n* = 144 for global/local task, and *n* = 134 for number/letter task.

For all tasks, a trial consisted of a sequence of four slides (Fig. 1a). First, the cue was presented 400–600 ms (randomly selected from a uniform distribution). The cue was followed by a blank screen for 400–600 ms (randomly selected from a uniform distribution), referred to as inter-stimulus interval (ISI). Then the target was presented until the participant responded, but always for a minimum of 1000 ms and a maximum of 3000 ms. Lastly, there was an inter-trial interval (ITI) of 1000–1500 ms (randomly selected from a uniform distribution), showing again a blank screen. The duration of one trial varied between 2800 and 5700 ms, depending on cue presentation time, ISI and ITI intervals, as well as participants’ response time (i.e., target presentation time).

In the *parity/magnitude task*, participants had to decide whether the target, a number between one and nine (except five), was either smaller or larger than five (rule-set magnitude, red) or whether it was odd or even (rule-set parity, green). This task was adapted from Sudevan et al.^50^ and had a total of 384 trials.

In the *global/local task*, the target was one of four geometric figures (circle, triangle, square, cross). Each figure was composed of small geometric shapes from the same set. The larger figure and the smaller shapes could never have the same geometrical shape. Participants had to indicate what shape a figure was (rule-set global, red) or what shapes it was made of (rule-set local, green). This task was adapted from Miyake et al.^4^ and had total of 384 trials.

In the *number/letter task*, the target was a number-letter pair (e.g., 3K). Participants had to decide whether the presented number was smaller or larger than five (rule-set number, red) or whether the letter was a vowel or consonant (rule-set letter, green). Numbers from one to nine (except five) and letters A, E, I, U, G, K, M, and R were used. This task was adapted from Rogers et al.^38^ and a total of 256 trials were presented.

Of all trials in each switching task, 50% were switch trials, requiring a shift in the rule-set, and 50% were repeat trials, requiring the same rule-set as in the trial before. Half of all trials belonged to each rule-set. Condition (switch vs. repeat) and rule-set were fully crossed. Trial conditions were randomized with the restriction that neither rule-set nor condition occurred more than three times in a row. Furthermore, none of the targets occurred twice in a row. Mapping of response keys was kept constant across participants.

Before starting the experimental trials, participants practiced the task, receiving feedback on the correctness of their response. The practice consisted of 10 trials for each rule-set followed by 20 trials, in which both rule-sets were intermixed. Participants were instructed to respond as fast and accurate as possible, by pressing one of two keys (parity/magnitude task and number/letter task) or one of four keys (global/local task) on the keyboard. If participants did not respond within 3000 ms of target onset, the trial was marked as missing and during practice participants received the feedback “too slow".

Slides were black with either red (#FF1920) or green (#00B21E) stimuli, depending on the rule-set. Numbers and letters were presented in typeface Arial and font size 40. All stimuli were centered horizontally and vertically.

As expected, and consistent with previous research, we observed longer reaction times in switch trials than in repeat trials across all three switching tasks. See Fig. 1b and c for reaction times and accuracy, respectively.

#### Intelligence

To assess fluid intelligence, we used the short version of the Berlin Intelligence Structure Test (BIS)^51^. Here, a total of 15 tasks were utilized to fully cross the four operation-related components of intelligence (processing capacity [PC], processing speed [PS], memory [M], and creativity [C]) with the three content-related components of intelligence (verbal, numerical, and figural). The short version of the BIS was completed in approximately 50-60 minutes and was conducted in groups of up to 4 participants. The operation-related component scores for each participant were calculated by aggregating the normalized *z*-scores of all tasks that measured the respective component. Participants had a mean IQ of 96 (SD = 16).

#### Working memory capacity

To evaluate the WMC of the participants, the Working Memory Test Battery, developed by Lewandowsky et al.^52^, was employed. Four tasks from the battery were utilized: the memory updating task, the operation span task, the sentence span task, and the spatial short-term memory task. However, due to an error in the programming code, we could not use the data of the spatial short-term memory task in our analyses. Furthermore, we employed the location-letter binding task, as described by Wilhelm et al.^53^. With the exception of six participants, all subjects completed this letter binding task. One participant did not take part in any of the WMC tasks. Additionally, four participants did not adhere to the task instructions of the operation span task. Consequently, their data was not included in the analyses. For each set size in the working memory tasks, the mean proportion of correctly solved items was calculated for each participant as the dependent variable.

### Procedure

The study comprised three measurement occasions, with data collected at three-month intervals. The duration of each measurement occasion was approximately 3.5 hours. Prior to their inclusion in the study, participants were required to sign an informed consent form at the beginning of the first session. They then completed the Ishihara test^54^ to ascertain that they exhibited no form of color blindness, as this could have affected the results of the study. In the initial two sessions, an electroencephalogram (EEG) was recorded while participants completed six distinct cognitive tasks on a computer (for details see Löffler et al.^5^). EEG was recorded in a dimly lit, electromagnetically shielded cabin. To minimize between-subjects error variance, the tasks were presented in a consistent sequence for the entire sample^55^. In the first session, participants completed the parity/magnitude task and the global/local task. Additionally, demographic data about the participants was gathered at the end of the first session. In the second session, the number/letter task was administered. The third session was conducted in groups of 1 to 4 participants. First, the BIS was administered as a paper-and-pencil test. Subsequently, the participants completed the WMC tasks on computers. Furthermore, participants completed two brief cognitive tests measuring cognitive abilities, a questionnaire assessing mind-wandering, and a pretzel task (the results of which are not presented here). From the initial sample of 148 participants attending session 1, six did not attend session 2 and eight did not attend session 3.

### EEG recording and quantification

#### Hardware

EEG was continuously recorded with 32 equidistant passive silver-silver chloride (Ag/AgCl) electrodes (EasyCap GmbH, Herrsching, Germany). See Supplementary Fig. S1 for the electrode layout. All sites were referenced online to Cz. Fpz served as ground. We used a BrainAmp DC amplifier with an input impedance of 10*MΩ* (Brain Products GmbH, Munich, Germany). Recordings, in AC mode, were sampled at 1000 Hz with a 0.1 μ*V* resolution. The impedances of EEG electrodes were kept below 5 kΩ. The pass-band of the hardware filter was set to 0.016–1000 Hz. Due to recording errors, data from two subjects in session one and four subjects in session two had to be discarded. Additionally, due to insufficient battery capacity, data from one subject each is missing for the parity/magnitude and number/letter tasks. EEG data were processed in MATLAB (R2021a, The MathWorks, Inc.) using the EEGLAB 2023.0 toolbox^56^.

#### Pre-processing for independent component analysis

EEG data were analyzed independently for each task. We segmented the data from the beginning to the end of each task and then performed the following pre-processing and data analysis steps. Raw EEG data were first pre-processed for running independent component analysis (ICA)^57^. ICA was used for removing eye, muscle, and heart artifacts. Data was high-pass filtered at 1 Hz using a zero-phase Hamming windowed sinc FIR filter implemented in EEGLAB^58^. Noisy channels were rejected using the channel criterion of the clean_artefacts function^59^. Channels were rejected if they correlated less then *r* = 0.80 to their neighboring channels. Across all tasks, on average 0.60 ± 0.83 (median = 0) channels were removed in each data set ranging from 0 to 4. Line noise was removed using Cleanline (v2.0) with the default settings^60,61^. Then the data was re-referenced to the average reference and the online reference Cz was recovered. Cz was then discarded after re-referencing to match the number of channels to the rank of the data for ICA. Cz was chosen because its activity is best captured by neighboring electrodes. Therefore, presumably the least amount of information is lost by discarding this electrode. Data were temporally segmented into consecutive two-second epochs and artefactual epochs were rejected automatically using threshold, joint probability, and kurtosis^62^. Epochs were rejected if activation exceeded amplitude thresholds of ±1000 μV. Furthermore, improbable data epochs were discarded on the assumption that epochs with artifacts are improbable. This was done by computing the probability distribution of all data points, deriving the probability of each data point, and defining the probability of an epoch by the joint probability of all data points within an epoch. Probability was calculated for single electrodes and for the entire set of electrodes. Improbability was determined by the standard deviation of the mean probability distribution. The threshold was set at five *SD* for single channels and two *SD* for all channels. Similarly, epochs were classified as artefactual if the kurtosis of the data point distribution within an epoch exceeded a threshold. Again, the threshold, determined as the standard deviation from the mean kurtosis values, was set at five *SD* for single channels and two *SD* for all channels. Rejection of an epoch always led to the rejection of this epoch in all channels. This automatic procedure led to an average of 12 ± 3% of rejected epochs (range: 5–20%) across all tasks. ICA was performed on the remaining segments, using the extended infomax algorithm^57,63^. ICs were automatically classified using ICLabel^64^. ICLabel provides the probability of each IC to be generated by brain, muscle, eye, heart, line noise, channel noise, and others. ICs were removed if the highest probability was generation by eye (if probability exceeded 25%), muscle (if probability exceeded 90%), and heart (if probability exceeded 60%). These ICs were removed by setting their weight to 0. Across all tasks on average 5.71 ± 2.45 (median = 6) ICs were removed for each data set, ranging from 1 to 16. The resulting weight matrix was saved.

#### Pre-processing for time-frequency analysis

In a second step, raw EEG data were pre-processed for analyzing time-frequency dynamics. Raw EEG data, segmented for the corresponding task, were high-pass filtered 0.1 Hz using a zero-phase Hamming windowed sinc FIR filter implemented in EEGLAB^58^. The same noisy channels, detected by the algorithm described above were removed, line noise was removed as described above, and data were re-referenced to average reference. To adjust the number of channels to the ICA weight matrix, the online reference Cz was restored before re-referencing and removed afterwards. The ICA matrix was imported, and EEG activity was re-calculated, with artefactual ICs removed. Finally, rejected channels were interpolated using a spherical spline interpolation. Epochs were extracted by segmenting −1750 to 2000 ms around cue onset for cue-related analyses and −2950 to 2500 ms around target onset for target-related analyses. The larger time window for target-related analyses was due to having a baseline epoch prior to cue-onset and including the full response period. Artefactual epochs were rejected automatically using the same methods and parameters as described above. Moreover, an intra-individual outlier analysis was conducted on reaction times. This analysis was based on all correct trials. Reaction times were classified as outliers if their log-transformed and *z*-standardized form fell outside a threshold of ±3SD from the mean reaction time of an individual^65,66^. These trials were rejected. Only trials with correct responses were kept for the time-frequency analyses. Overall, across switching tasks and cue- and target-related analyses, 20 ± 5% (range: 9–42%) of epochs were rejected: 5 ± 4% due to errors, 1 ± 1% due to reaction time outliers, and 15 ± 4% due to EEG artifacts. Rejection rates for trials were within the expected range for errors^67^, reaction time outliers^65^, and EEG artifacts^62^. Finally, to achieve a similar signal-to-noise ratio between the conditions (switch and repeat), the number of trials per condition was equalized individually for each subject by randomly selecting *n* trials from the condition with the higher number of trials (where *n* is the number of trials in the condition with the lower number of trials).

#### Time-frequency analysis

To obtain the time-frequency representation of EEG power we calculated complex Morlet wavelets, adapting code provided by Cohen et al. (chapter 13)^68^. We extracted 25 frequencies logarithmically spaced between 3 and 60 Hz. The number of wavelet cycles changed as a function of frequency and ranged between 3 and 6. To account for edge artifacts, 1000 ms at the beginning and end of each epoch were discarded after wavelet convolution. For cue-related analyses, this resulted in epochs from −750 to 1000 ms relative to cue onset, with activity from −750 to −250 ms serving as baseline. For target-related analyses, epochs ranged from −1950 to 1500 ms relative to target onset, with activity from −1950 to −1450 ms serving as the baseline period. The target-related baseline period preceded cue onset regardless of cue presentation duration and ISI. Baseline activity was calculated across both conditions. We then averaged the mean instantaneous power over trials independently for each condition. The resulting time-frequency power spectrum was normalized to the baseline using decibel conversion. Finally, we downsampled the time-frequency representation from the initial sampling rate of 1000 Hz to 50 Hz.

### Statistical analyses

All statistical analyses were conducted with R (v3.6.3)^69^ or MATLAB (R2021a, The MathWorks, Inc.), except otherwise specified. For mass-univariate analyses, we only included participants achieving a mean accuracy above or equal to 70% in the cognitive flexibility tasks. This led to the exclusion of data from three participants in the parity/magnitude task, from two participants in the global/local task, and three participants in the number/letter task. As one of these participants did not attend sessions 2 and 3, we had no viable data for that participant. This reduced the total sample size to 147. Additionally, we performed inter-individual outlier analyses for all manifest variables included in the structural equation models. Therefore, variables were *z*-transformed, after ensuring their approximate normal distribution^70^. A value was set to missing, if its absolute *z*-standardized form was larger than three. After the inter-individual outlier analysis, we *z*-standardized the variables again for further SEM analyses. We estimated the reliability of all manifest indicator variables in the structural equation models. We used Cronbach’s *α* for WMC and intelligence scores and Spearman-Brown corrected odd-even split correlations for the electrophysiological and behavioral data.

#### Mass-univariate analyses with cluster-based permutation testing

To examine how instantaneous power in response to the cue and target differed between conditions, we used mass-univariate analyses^41^. For each voxel (representing EEG power of one time point, frequency, and electrode), we performed a parametric repeated measures *t*-test with the factor condition (switch vs. repeat). Significance level was set to *p* = 0.005^71^. We corrected for multiple comparisons using cluster-based permutation tests^41,72^. Clusters are defined as significant voxels that are connected in time, frequency, and space. For this purpose, neighboring electrodes were defined as all electrodes that were directly adjacent to the seed electrode. Only voxels showing effects in the same direction were included in a cluster. The size of each empirical cluster was then compared to a distribution of cluster sizes derived from permuted data. Therefore, mass-univariate analyses were conducted on permuted data. In permuted data, the condition labels of trials have been randomly shuffled. For each permutation, the size of the largest cluster detected is extracted and contributes to the null distribution. We included 1000 permutations and determined cluster significance by the joint examination of *t*-mass (sum of all *t* values within a cluster) and cluster size (amount of significant voxels within a cluster). In practice, these two criteria consistently yielded the same outcomes in the current study. The permutation test reveals whether a cluster of a specific extent is likely to be present in data where the condition labels of trials have been randomly shuffled (permuted data). If the extent of a cluster found in the original data is highly unlikely (*p* < 0.05) in permuted data, the empirical cluster is interpreted as representing meaningful condition differences. All other significant voxels are interpreted as chance findings. Here, significance level was set to *p* = 0.05, in order to explore all potentially relevant clusters^71^. Importantly, cluster-based permutation tests neither reveal the exact time period when significant differences are present nor the exact frequency range or location of the detected effect, as they are based on probabilistic test statistics^73^.

#### Structural equation modeling

In a first step, we evaluated whether differences in event-related spectral perturbations (ERSP) between task conditions within a cluster do load onto a common flexibility factor across tasks. Therefore, ERSP values within the core region of significant clusters were averaged and served as manifest variables in the structural equation models. Core regions were defined because cluster are subject to uncertainties regarding the spatial, temporal, and spectral extent^73^. The core regions of the clusters were defined by the effect sizes ${\omega }_{p}^{2}$ of the voxels contributing to the cluster. For this purpose, the effect sizes of a cluster were first logarithmized and then the mean plus one standard deviation was calculated as a cut-off value. Only those voxels whose effect size was greater than the cut-off value contributed to the core region. If this restriction caused clusters to split into different parts, only the largest sub-cluster was considered.

We modeled flexibility using a latent change score model^74,75^, as it addresses the issue of poor reliability of manifest difference scores. In line with previous research^76,77^, the reliabilities of the cue-related manifest difference scores were found to be poor to unacceptable, with values ranging from 0.00 to 0.10 for theta and from 0.35 to 0.53 for alpha/beta in our data. Similarly, reliabilities of the target-related manifest difference scores reliabilities were found to be poor to unacceptable, with values ranging from 0.40 to 0.53 for alpha/beta. Because a latent change model captures the difference between two or more (repeated-measures) conditions on the latent level, it overcomes this critical limitation of manifest difference score. In the models we implemented, the two conditions of each task contribute to a switch and a repeat factor, respectively. Flexibility is modeled as the residual variance, when the switch factor is regressed on the repeat factor with a *β*-weight of 1. At the manifest level, a difference score is computed by directly subtracting one observed variable from another. The latent change score model is conceptually analogous in that it also captures the difference between two timepoints; however, it does so by modeling change as a latent variable, thereby partitioning observed variance into true score variance and measurement error. Critically, this means the latent change score estimates *true* change, disentangled from random measurement error, whereas the raw difference score conflates genuine change with error variance from both measurement occasions—amplifying unreliability rather than canceling it. This is a fundamental psychometric liability of manifest difference scores: because their reliability is bounded by, and typically lower than, the reliability of either constituent measure, they tend to attenuate correlations with external variables and inflate within-person variability spuriously. The latent change score framework circumvents these problems by imposing the measurement model directly, yielding estimates of change that are corrected for attenuation and more accurately reflect the underlying construct of interest (see refs. ^29^,^31^ for a more detailed explanation). Thereby, the latent difference overcomes the psychometric problems that are associated with manifest difference scores. We added *m* − 1 method factors, where *m* is the number of tasks. By including method factors, we account for task-specific associations between ERSPs. The use of *m* − 1 over *m* method factors is advantageous because it results in a globally identified model^78,79^. Here, the parity/magnitude task served as the comparison standard, while we specified one method factor loading on all ERSP values measured in the global/local task and a second method factor loading on all ERSP values measured in the number/letter task. The method factors are thus defined as residual factors common to all ERSP values measured within the same task. Because all manifest variables had been *z*-transformed, the intercepts were set to zero.

In a second step, we combined the measurement models of flexibility into one single model. In addition to flexibility we modeled intelligence by using the four operation-related components of intelligence (processing capacity [PC], processing speed [PS], memory [M], and creativity [C]). Furthermore, WMC was modeled using the memory updating task, the operation span task, the sentence span task, and the location-letter binding task. With this model, we investigated the latent correlations between cognitive flexibility, general cognitive abilities, and WMC.

All models were fitted with the R package *lavaan*^80^, using the maximum likelihood algorithm. We used full information maximum likelihood (FIML) to account for missing data. We evaluated the goodness of fit of the models using the comparative fit index (CFI)^81^ and the root mean square error of approximation (RMSEA)^82^. We considered a CFI of >0.90 and an RMSEA of <0.08 to indicate an acceptable fit of the model. A CFI >0.95 and an RMSEA < 0.06 indicated a good model fit^82,83^. The two-sided critical ratio test was used to assess the statistical significance of the model parameters. While we tested the path coefficients on the unstandardized scale, we report standardized coefficients (*β*) and 95% confidence intervals (95% CIs) as effect-size indices. We use Akaike weights^84^, which are based on the Akaike information criterion^85^, to compare model fits.

For the latent-change structural equation model (*df* = 162), with a sample size of *N* = 147, and an alpha error of *α* = 0.05, we can test the hypothesis of close fit (H0: *ε *≤ 0.05, H1: *ε* ≥ 0.08)^86^) with a power of 1−*β* = 0.96. However, for the measurement models estimated in the first step (*df* = 12), the hypothesis of close fit can only be tested with low statistical power (1 − *β* = 0.25).

#### Drift-diffusion modeling

We employed the drift-diffusion model (DDM)^87^ to obtain process-based measures of cognitive flexibility performance. The drift-diffusion model conceptualizes binary decisions as a stochastic evidence-accumulation (random-walk) process that continues until the accumulated evidence reaches one of two decision thresholds. At this point, the response is executed.

In previous work, we demonstrated problems with behavioral measures for both simple reaction time data and model-based drift rate *v*^5^. These measures are incapable of isolating executive functions from mere processing speed. However, in the present paper we argue that the latent change model is a different and promising way of isolating pure cognitive flexibility processes. Therefore, demonstrating whether this approach is capable of capturing cognitive flexibility with behavioral measures and examining its relation with neural measures might expand our understanding of cognitive flexibility.

Several parameters are estimated: the drift rate *v* indexes the average increase of evidence per time unit, the boundary separation *a* reflects the distance between the correct and alternative thresholds, the starting point *z* models response bias, and the non-decision time *t*_0_ captures the duration of processes that are not directly involved in decision making, such as perceptual encoding and motor execution. Parameters were estimated for each participant with *fast-dm-30*^88^ using the Kolmogorov–Smirnov criterion to fit the observed reaction time and accuracy distributions^89^. We estimated *v*, *a*, *t*_0_ and the inter-trial variability of *t*_0_ (*st*_0_), fixed all other parameters to zero, and centered *z* at 0.5, following standard recommendations^90^. The parameters *t*_0_ and *v* were estimated separately for the two experimental conditions, switch and repeat. All other parameters were estimated jointly across conditions. Here, we use the non-decision time *t*_0_ and drift rate *v* as manifest indicators in the structural equation models, as these have been proven to be relevant for explaining switch costs. Non-decision time has been associated with anticipatory task-set preparation, while drift rate has been associated with the strength of task-set activation from the previous trial^91,92^.

## Results

### Time-frequency dynamics in response to the cue

**Fig. 2:**
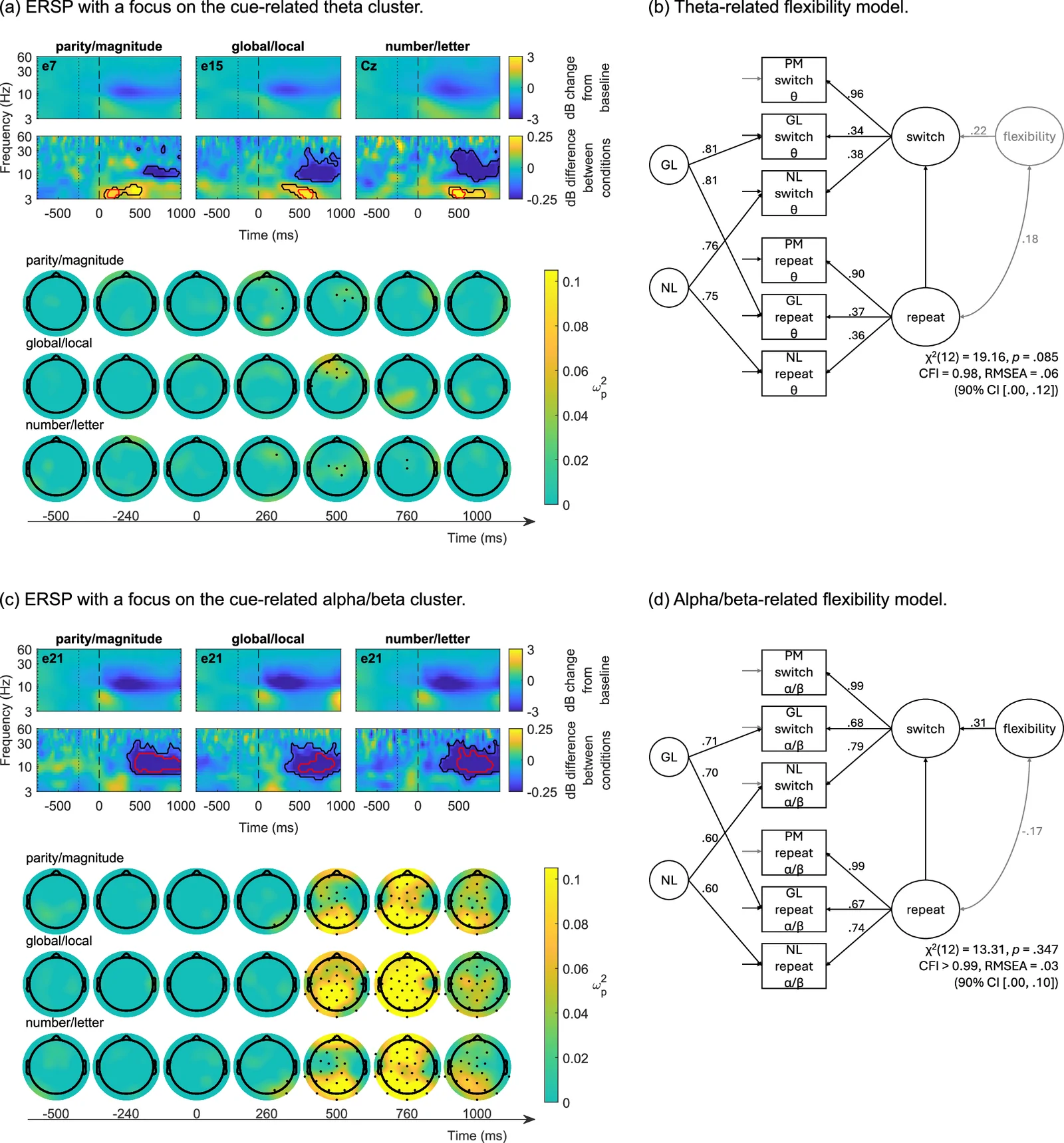
Event-related spectral perturbations in response to the cue (left columns) and latent-change models of cognitive flexibility (right columns). **a** ERSP with a focus on the cue-related theta cluster. **b** Theta-related flexibility model. **c** ERSP with a focus on the cue-related alpha/beta cluster. **d** Alpha/beta-related flexibility model. **a**, **c** The first rows show the dB change of power in relation to the baseline (−750 to −250 ms relative to cue onset) averaged across both conditions. The name of the depicted electrode is shown in the top-left corner. The second row displays the dB difference between the conditions (switch minus repeat) at the same electrodes. Positive values represent greater power in switch compared to repeat trials, while negative values represent lesser power. The black border surrounds voxels belonging to a cluster, and the red border indicates voxels belonging to the core region of that cluster. The lower part of (**a**) and (**c**) shows the topographic distribution of effect sizes ${\omega }_{p}^{2}$, derived from univariate tests contrasting the two conditions, at an exemplary frequencies of (**a**) 5 Hz for the theta cluster and (**c**) 10 Hz for the alpha/beta cluster. Additionally, electrodes contributing to the respective clusters are depicted. **b,** **d** Latent-change models of cognitive flexibility based on mean power of the core region of the (**b**) theta and (**d**) alpha/beta cluster. The standardized path weights and correlation coefficients are shown next to the paths. Non-significant estimators are grayed out. Included cases were *n* = 142 for parity/magnitude task, *n* = 144 for global/local task, and *n* = 134 for number/letter task. In total *N* = 147 participants were included in the structural equation models and we used full information maximum likelihood (FIML) to account for missing data.

We first describe the general spectral response to the cue, irrespective of condition. In all tasks and across conditions, the brain exhibited a general increase in theta power within the initial 400 ms following cue onset, most pronounced in the lateral occipital regions, and a general decrease in alpha/beta power beginning at 100 ms after cue onset (see first row of Fig. 2a and c for frontal and occipital electrodes, respectively). The alpha/beta power decrease persisted throughout the remainder of the analysis period. It was descriptively strongest in the lateral occipital cortices. However, it was also present in the broader frontal region.

In a second step, we then examined condition differences as a prerequisite to isolate flexibility-specific cognitive processes. We found two distinct clusters across all three tasks in the empirical data, representing differences between conditions in theta power increase and alpha/beta power decrease in response to the cue (see second row of Fig. 2a, c). Exact cluster statistics for each task can be found in Supplementary Table S1.

The first cluster reflected a significantly greater theta increase in switch trials compared to repeat trials. Over the three tasks, this effect emerged at fronto-central electrodes roughly 300–550 ms after cue onset and explained about 3% of the variance in theta power (lower part of Fig. 2a). Condition differences were most pronounced 100 to 200 ms after cue onset for the parity/magnitude task and 500–600 ms after cue onset for the other two tasks, explaining up to 7% of the variance (Table 1).

**Table 1:** Statistical description of the core regions of significant, cross-task generalizable clusters for cue- and target-related analyses

Task	${\omega }_{p}^{2}$-cutoff	Frequency range	Time range	*t*-mass	Cluster size	${\omega }_{p}^{2}$		Contributing electrodes
		*Hz*	*ms*	*sum*	#*voxel*	*mean* ± *SD*	*95% CI*	
Cue-related theta cluster								
Parity/magnitude	>0.036	3.4–4.4	110–230	98.91	27	0.042 ± 0.004	[0.040, 0.044]	6 7
Global/local	>0.045	3.0–4.9	470–650	229.48	56	0.052 ± 0.005	[0.050, 0.054]	6 7 15 25
Number/letter	>0.043	3.4–4.4	430–570	82.03	21	0.051 ± 0.006	[0.048, 0.054]	3 Cz
Cue-related alpha/beta cluster								
Parity/magnitude	>0.088	7.2–28.4	390–990	−6590.43	1047	0.121 ± 0.028	[0.119, 0.123]	3 4 5 10 11 12 13 14 15 20 21 22 25 28 29 30 Cz
Global/local	>0.108	6.3–22.1	390–930	−11,250.80	1640	0.138 ± 0.027	[0.136, 0.140]	all electrodes except 8 18 26 27
Number/letter	>0.091	8.1–25.0	310–910	−6252.66	1028	0.119 ± 0.025	[0.117, 0.121]	all electrodes except 9 17 18 23 26 27 Cz
Target-related alpha/beta cluster								
Parity/magnitude	>0.067	8.1–28.4	−310–490	−7020.33	1293	0.092 ± 0.023	[0.090, 0.094]	1 2 3 4 5 6 11 12 13 14 15 16 20 21 22 25 28 29 30 31 Cz
Global/local	>0.086	7.2–28.4	−270–310	−15,431.97	2432	0.121 ± 0.029	[0.119, 0.123]	all electrodes except 8 18 26 27
Number/letter	>0.072	7.2–25.0	−330–610	−8914.80	1641	0.097 ± 0.022	[0.095, 0.099]	1 2 3 4 5 6 7 8 10 11 12 13 14 15 16 20 21 22 25 28 29 30 31 Cz

A core region is defined as voxels with an effect size greater than the mean plus one standard deviation of the corresponding cluster’s effect sizes (${\omega }_{p}^{2}$-cutoff). Included cases were *n* = 142 for parity/magnitude task, *n* = 144 for global/local task, and *n* = 134 for number/letter task.

In addition, there was a cluster showing a significantly greater decrease in alpha/beta power in switch trials compared to repeat trials. This difference was present throughout the cortex, but was most pronounced at left lateral parietal sites (lower part of Fig. 2c). The difference was present approximately 100–200 ms after cue onset and persisted until the end of the extracted time period (1000 ms after cue onset). It explained about 6% of the variance in alpha/beta power. The differences in condition were most evident between 400 and 900 ms after cue onset, explaining up to 25% of the variance (Table 1).

That is, while we observed a general theta and alpha/beta response irrespective of condition, we additionally find differences in these spectral responses between conditions. These differences appear in similar frequency ranges, but they differ in their temporal and spatial characteristics from the general condition-independent response.

### Testing for a common flexibility factor in cue-related power modulations

To test whether these condition-specific power modulations index a common neural mechanism of cognitive flexibility that generalizes across tasks, we averaged the ERSP separately for each cluster’s core region, task, and condition. We then used these averaged power values as indicators for the latent factor flexibility in a structural equation model. See Table 1 for an overview of the core regions.

Supplementary Table S2 presents the reliabilities of the manifest indicator variables used in the structural equation models and their zero-order correlations. The reliabilities of the condition-specific ERSP values were found to be excellent for alpha/beta (≥0.95) and mostly acceptable or good for theta (0.65–0.86).

In a first step, we tested whether the detected condition-specific power changes load onto a common flexibility factor across tasks separately for each ERSP cluster. Specifically, we modeled flexibility as the latent difference between both task conditions using a latent change model to account for the unreliability of manifest difference scores. The latent change model with cue-related theta ERSP explained the data well, *χ*^2^(12) = 19.16, *p* = 0.085, CFI = 0.98, RMSEA = 0.06, 90% CI [0.00, 0.12], AIC = 1958.52 (Fig. 2b). However, flexibility could not be captured by this model, as the variance of the latent change factor was not significant (${\sigma }_{{{\rm{unstandardized}}}}^{2}$ = 0.04, 95% CI [−0.09, 0.18], *p* = .517). In order to interpret these null findings, we compared this model with a more constrained latent change model, where we fixed the variance of the flexibility factor and its correlation with the repeat factor to zero. If there is indeed no shared flexibility-specific variance, the constrained latent change model should fit the data as good or even better as the initial model. Indeed, model fit remained good, *χ*^2^(14) = 20.03, *p* = 0.129, CFI = 0.99, RMSEA = 0.05, 90% CI [0.00, 0.11], AIC = 1955.39. According to the Akaike weights (initial model: 0.17; constrained model: 0.83), the constrained model is 4.80 times more likely than the unconstrained model. Regarding the cue-related alpha/beta cluster, the latent change model provided a good explanation of the data, *χ*^2^(12) = 13.31, *p* = 0.347, CFI > 0.99, RMSEA = 0.03, 90% CI [0.00, 0.10] (Fig. 2d). Here, flexibility could be captured, as the variance of the latent change factor was significant (${\sigma }_{{{\rm{unstandardized}}}}^{2}$ = 0.09, 95% CI [0.05, 0.13], *p* < 0.001). Baseline alpha/beta power (i.e., in the repeat condition) was not associated with the magnitude of change (*r* = −0.17, *p* = 0.175, 95% CI [−0.40, 0.06]).

In conclusion, the detected condition-specific power changes did only reflect task-generalizable flexibility in case of alpha/beta power, but not in case of theta power.

### Time-frequency dynamics in response to the target

**Fig. 3:**
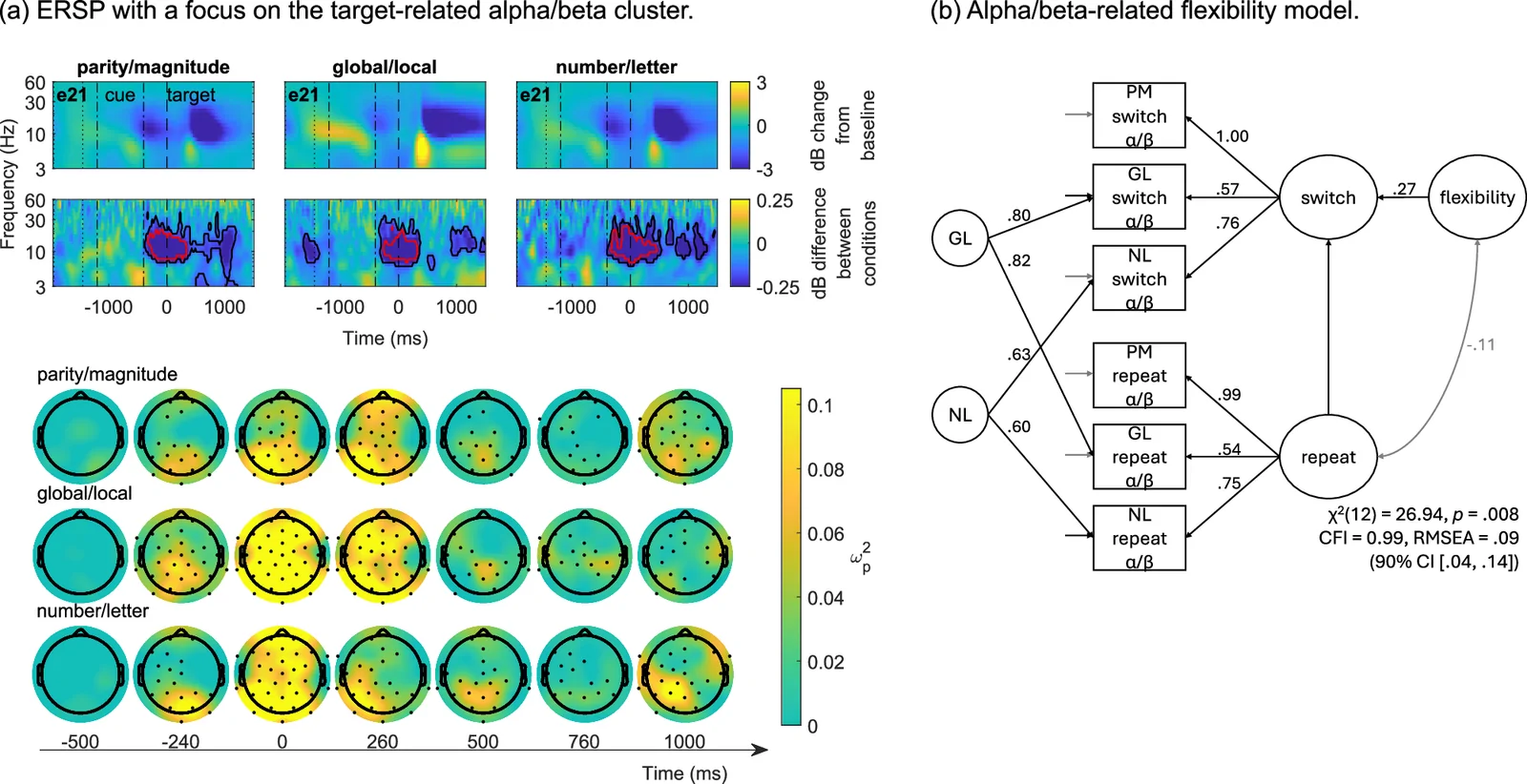
Event-related spectral perturbations in response to the target (left) and latent-change model of cognitive flexibility (right). **a** ERSP with a focus on the target-related alpha/beta cluster. **b** Alpha/beta-related flexibility model. **a** The first row shows the dB change of power in relation to the baseline (−1950 to −1450 ms relative to target onset) averaged across both conditions. The dashed-dotted lines mark the period during which the cue was presented. The name of the depicted electrode is shown in the top-left corner. The second row displays the dB difference between the conditions (switch minus repeat) at the same electrodes. Positive values represent greater power in switch compared to repeat trials, while negative values represent lesser power. The black border surrounds voxels belonging to a cluster, and the red border indicates voxels belonging to the core region of that cluster. The lower part of (**a**) shows the topographic distribution of effect sizes ${\omega }_{p}^{2}$, derived from univariate tests contrasting the two conditions, at an exemplary frequency of 10 Hz for the alpha/beta cluster. Additionally, electrodes contributing to the respective cluster are depicted. **b** Latent-change model of cognitive flexibility based on mean power of the core region of the alpha/beta cluster. The standardized path weights and correlation coefficients are shown next to the paths. Non-significant estimators are grayed out. Included cases were *n* = 142 for parity/magnitude task, *n* = 144 for global/local task, and *n* = 134 for number/letter task. In total *N* = 147 participants were included in the structural equation model and we used full information maximum likelihood (FIML) to account for missing data.

The following two sections correspond to the two preceding ones, which focused on cue-related oscillations, now focusing on neural dynamics triggered by the target stimulus. Similarly to the cue-related power changes, in all tasks and across conditions, the brain exhibited a general increase in theta power around 400 to 500 ms following target onset, most pronounced at left and right lateral occipital electrode sites, and subsequently a general decrease in alpha/beta power lasting until 1000 ms post target for the parity/magnitude and number/letter task (first row of Fig. 3a). For the global/local task, the alpha/beta decrease endured until 1500 ms post target. Not only was the alpha/beta decrease more persistent in the global/local task compared to both other tasks, also was the theta increase descriptively strongest in this task and visible throughout the cortex. The alpha/beta power decrease was descriptively strongest at lateral occipital electrode sites. However, it was also present in the broader frontal region. In summary, we observed a general, condition-independent, neural response to the target that resembled the response to the cue.

Turning attention to the condition-specific neuronal responses, cluster-based permutation tests revealed three distinct clusters in the empirical data, representing differences between the switch and repeat conditions. As in response to the cue, conditions differed regarding the alpha/beta power decrease. In addition, statistical analysis of the data revealed a condition difference in a target-related theta increase and beta decrease. However, only the alpha/beta cluster generalized across all three tasks, while the theta and beta cluster did only emerge in the parity/magnitude and number/letter task, but not in the global/local task. Exact cluster statistics for all three clusters can be found in Supplementary Table S1. The core region of the alpha/beta cluster is described in Table 1. The following paragraphs describe the detected clusters in more detail.

A significantly greater decrease in alpha/beta power occurred in switch trials compared to repeat trials in response to the target (second row of Fig. 3a). This difference was present throughout the cortex, but was most pronounced at left lateral parietal electrode sites (lower part of Fig. 3a). The difference emerged approximately 300 ms before target onset and persisted until the end of the extracted time period (1500 ms after cue onset). It explained about 6% of the variance in alpha/beta power. The difference was most pronounced −300 to 500 ms around target onset and explained up to 26% of the variance during this time window (Table 1). Alpha/beta effects are likely to be modulated by the tasks context prior to target onset, as participants prepare for the upcoming target after cue presentation and engage in preparatory processes up until the target can be processed.

The theta cluster manifested at right fronto-central sites, clearly before target onset for the number/letter task (−350 to 150 ms; core region: −270 to −70 ms) and with target onset for the parity/magnitude task (−210 to 490 ms: core region: 10–290 ms; Supplementary Fig. S2a). The clear temporal onset before the target in the number/letter task suggests a methodological artifact. Because the target epoch contains a pre-cue baseline and the cue presentation period, cue-related activity inevitably appears in the target window, rendering the early theta increase a methodological artifact rather than a genuine target-related effect. Since cue-related dynamics were already modeled separately, this cluster was omitted from further analyses of cognitive flexibility. Consequently, only for the parity/magnitude task, there is a significant target-related theta effect. Based on this pattern of findings, the target-related theta effect was not further considered as a general neural marker for cognitive flexibility.

The beta cluster, like the theta cluster, was only present in the parity/magnitude and number/letter task. A substantially larger decrease in beta power was observed in switch trials relative to repeat trials, indicating a significant relationship between target-related power changes and trial type. This difference was evident at right lateral central electrode sites between 700 and 1500 ms following target onset (Supplementary Fig. S2b). It accounted for approximately 4–5% of the variance in beta power. The difference was most pronounced 1050–1400 ms after target onset, explaining up to 12% of the variance during this time window. However, the fact that the mean latency of condition differences in the beta cluster exceeded participants’ mean reaction time suggests that this cluster is unlikely to reflect target-induced cognitive flexibility processes. Consequently, this cluster, like the theta cluster, was not further tested as a potential neural signature of cognitive flexibility.

Of all three detected frequencies, only the alpha/beta cluster showed consistent cross-task effects, suggesting it may reflect a neural mechanism of task switching. The early emergence and sustained nature of this alpha/beta effect might imply anticipatory control processes are engaged before target onset. The theta effect, on the other hand, appears task-specific and potentially confounded by cue-related activity, limiting its generalizability. Late beta effects likely reflect post-decision or motor-related processes rather than core flexibility mechanisms.

### Testing for a common flexibility factor in target-related power modulations

To determine whether target-related alpha/beta dynamics reflect a shared underlying flexibility construct across tasks, we tested whether these power modulations load on a common latent flexibility factor. Therefore, we averaged target-related power changes within the core region of the alpha/beta cluster. The mean was calculated independently for each task and condition. Comparable with the cue-related values, reliabilities of the averaged target-related condition-specific ERSP values (Supplementary Table S2) were found to be excellent for alpha/beta (≥0.96).

The target-related latent change model converged and fit well to the target-related alpha/beta ERSP data, *χ*^2^(12) = 26.94, *p* = 0.008, CFI = 0.99, RMSEA = 0.09, 90% CI [0.04, 0.14] (Fig. 3b). Flexibility was captured by this model, as the variance of the latent change factor was significant (${\sigma }_{{{\rm{unstandardized}}}}^{2}$ = 0.08, 95% CI [0.04, 0.11], *p* < 0.001). Again, baseline alpha/beta power (i.e., in the repeat condition) was not associated with the magnitude of change: participants with higher baseline alpha/beta did not show significantly larger or smaller decreases than those with lower baseline alpha/beta (*r* = −0.11, *p* = 0.390, 95% CI [−0.35, 0.14]). Therefore, the model supports a latent flexibility factor underlying target-related alpha/beta suppression, independent of baseline power levels.

### Association between cognitive flexibility, intelligence, and working memory capacity

**Fig. 4:**
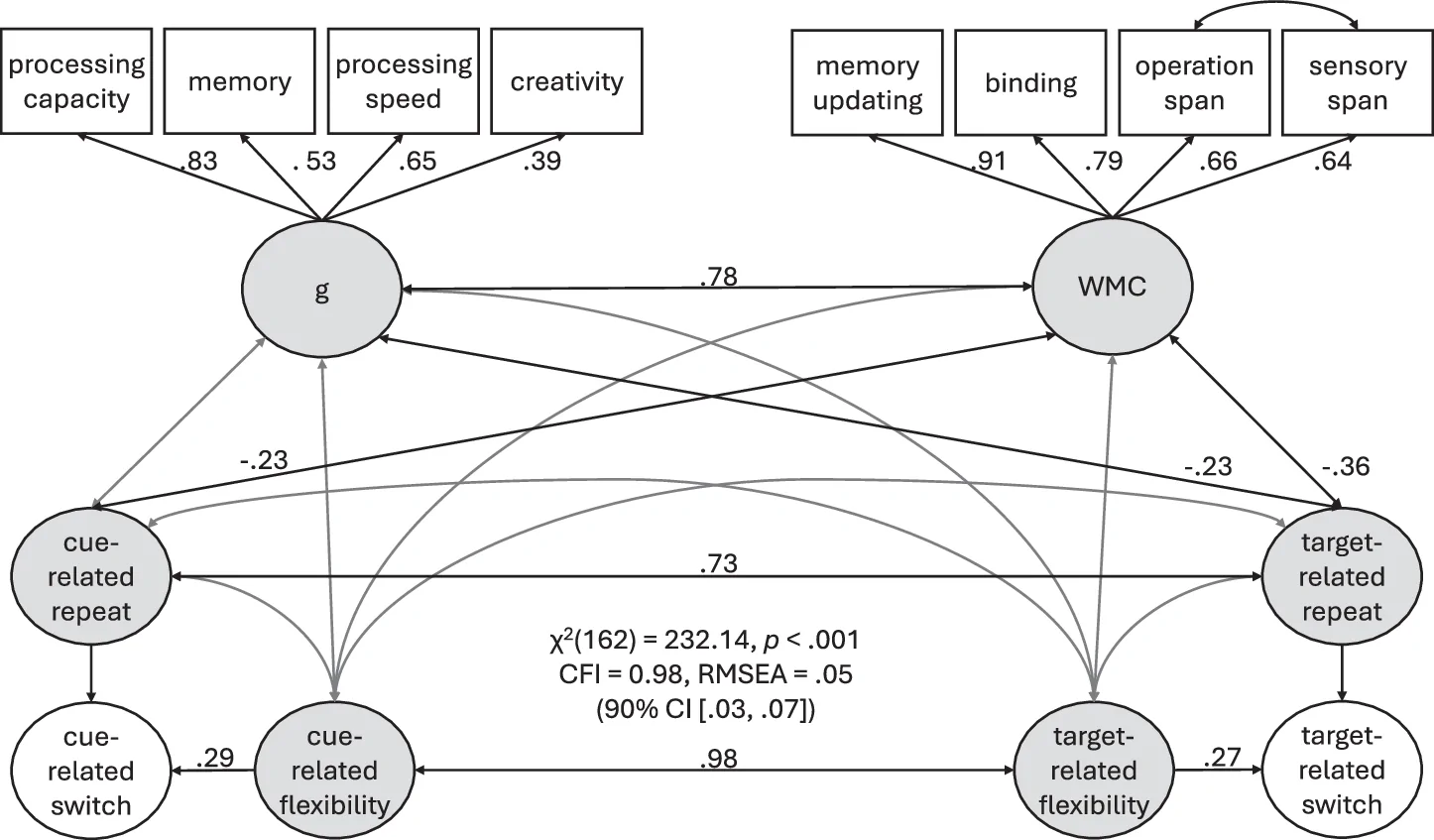
Combined latent-change models of cognitive flexibility based on alpha/beta power, including intelligence (g) and WMC. The standardized path weights and correlation coefficients are shown next to the paths. Non-significant estimators are grayed out. Included cases were *n* = 139 for memory updating and sentence span, *n* = 133 for binding, *n* = 135 for operation span, *n* = 140 for BIS, *n* = 142 for parity/magnitude task, *n* = 144 for global/local task, and *n* = 134 for number/letter task. In total *N* = 147 participants were included in the structural equation model and we used full information maximum likelihood (FIML) to account for missing data.

After determining suitable measurement models for flexibility, in a second step, we integrated the measurement models across clusters and explored their relation to general cognitive abilities and WMC. As evident from Supplementary Table S2, WMC could be measured with good reliability (>0.80), while for the BIS, with reliabilities ranging from 0.45–0.75, only processing capacity had a sufficient reliability. Note that this is typical for the short form of the BIS. This is why it is recommended to only interpret the general ability factor, as we do (see Fig. 4), rather than the operation-related manifest scores^51^.

We only included the two alpha/beta clusters in the integrated model, as these were capable of capturing common variance across the three flexibility tasks. In the integrated model, we allowed correlations between residual variances within condition-specific cue- and target-related activity (e.g., alpha/beta ERSP in the switch condition of the global/local task in response to the cue and the target). Furthermore, we allowed the residual variances of the sentence span and operation span tasks of the WMC battery to correlate, as these tasks are highly similar.

The model explained the data well, *χ*^2^(162) = 232.14, *p* < 0.001, CFI = 0.98, RMSEA = 0.05, 90% CI [0.03, 0.07] (Fig. 4). Cue-related and target-related flexibility could be captured by this model, as both latent change factors were significant (cue: ${\sigma }_{{{\rm{unstandardized}}}}^{2}$ = 0.08, 95% CI [0.05, 0.12], *p* < 0.001; target: ${\sigma }_{{{\rm{unstandardized}}}}^{2}$ = 0.07, 95% CI [0.04, 0.11], *p* < 0.001). That is, both cue- and target-related dynamics reflect meaningful, measurable flexibility constructs. The two latent flexibility factors were highly correlated (*r* = 0.98, *p* < 0.001, 95% CI [0.95, 1.00]). The same was true for cue- and target-related alpha/beta power in the repeat condition (*r* = 0.73, *p* < 0.001, 95% CI [0.65, 0.82]). Such a high overlap between cue- and target-related flexibility might suggest shared neural mechanisms. Furthermore, cue- and target-related repeat factors, reflecting basic stimulus processing, showed significant latent correlations with WMC (cue: *r* = −0.23, *p* = 0.016, 95% CI [−0.40, −0.05]; target: *r* = −0.36, *p* < 0.001, 95% CI [−0.53, −0.20]), indicating a weak to moderate negative relationship. Additionally, the target-related, but not the cue-related, repeat factor exhibited a weak negative association with general cognitive abilities (*r* = -0.23, *p* = 0.020, 95% CI [−0.42, −0.04]). Except for the substantial latent correlation between intelligence and WMC (*r* = 0.78, *p* < 0.001, 95% CI [0.66, 0.89]), none of the other correlations were significant (−0.17 ≤ *r* ≤ 0.16; cf. Supplementary Table S3 for full statistics).

### Convergence of neural and behavioral flexibility measures

Investigating behavioral measures in conjunction with neural measures can improve our understanding of a construct. Accordingly, we tested whether cognitive flexibility can also be isolated using non-decision time *t*_0_ and drift rate *v*. These process-based behavioral measures are derived from the drift-diffusion model^87^. Non-decision time *t*_0_ reflects the duration of processes unrelated to decision-making, including perceptual and motor response components, whereas drift rate *v* reflects the speed of information uptake^93^.

Reliabilities of the manifest indicator variables used in the structural equation models and their zero-order correlations can be found in Supplementary Table S4. The reliabilities of the non-decision time *t*_0_ and drift rate *v* were found to be good (≥0.84). The distribution of parameter estimates is shown in Supplementary Fig. S3.

The latent change measurement models indicated that cognitive flexibility could indeed be captured with non-decision time *t*_0_, *χ*^2^(12) = 20.71, *p* = 0.055, CFI ≥0.99, RMSEA = 0.07, 90% CI [0.00, 0.12], ${\sigma }_{{{\rm{unstandardized}}}}^{2}$ = 0.10, 95% CI [0.03, 0.17], *p* = 0.002, and the drift rate *v*, *χ*^2^(12) = 8.27, *p* = 0.763, CFI ≥0.99, RMSEA = 0.00, 90% CI [0.00, 0.06], ${\sigma }_{{{\rm{unstandardized}}}}^{2}$ = 0.09, 95% CI [0.02, 0.16], *p* = 0.004. The latent flexibility factors for both non-decision time and drift rate were significant, indicating meaningful flexibility-related individual differences in these model parameters.

Consequently, we integrated the DDM-based measurement models of flexibility with the two ERSP-based measurement models of flexibility that use alpha/beta power to isolate flexibility processes. We allowed correlations between all latent change residuals, between all latent factors representing basic cognitive processes (i.e., repeat factors), and between the latent change residual and the repeat factor within each domain.

The integrated model fitted the data adequately well, *χ*^2^(246) = 452.70, *p* < 0.001, CFI = 0.96, RMSEA = 0.08, 90% CI [0.06, 0.09]. The latent correlations revealed that flexibility measured via non-decision time was significantly associated with flexibility measured with cue-related alpha/beta power (*r* = 0.25, 95% CI [0.02, 0.49], *p* = 0.040). This association was weaker and not significant for target-related alpha/beta power (*r* = 0.17, 95% CI [−0.07, 0.41], *p* = 0.152). On the contrary, drift rate did not share a substantial amount of variance with flexibility measured with cue-related (*r* = 0.15, 95% CI [−0.10, 0.40], *p* = 0.227) nor target-related alpha/beta power (*r* = 0.17, 95% CI [−0.07, 0.42], *p* = 0.171). In contrast, the repeat factor derived from drift rates correlated both with cue-related (*r* = −0.21, 95% CI [−0.38, −0.05], *p* = 0.016) and target-related (*r* = −0.39, 95% CI [−0.53, −0.24], *p* < 0.001) repeat factors, supporting the notion that oscillatory activity and evidence accumulation are related, just not when reflecting flexibility specifically.

As had already been shown in the main analyses, cue- and target-related flexibility were highly correlated, while shared variance between the cue-related and target-related repeat factor was substantially smaller, but still large. Conversely, neither *t*_0_- and *v*-related flexibility nor *t*_0_- and *v*-related repeat measures correlated significantly with each other. As in our main analyses, we found that neuronally based flexibility and repeat factors were not substantially correlated. In contrast, for behavioral flexibility we found that participants with longer non-decision time and slower drift rate in the repeat condition tended to show smaller switch costs (*t*_0_: *r* = −0.42, 95% CI [−0.76, −0.08], *p* = 0.060; *v*: *r* = −0.22, 95% CI [−0.44, −0.00], *p* = 0.053).

Full statistics for all latent correlations are reported in Supplementary Table S5.

## Discussion

Our main goal was to identify oscillatory mechanisms underlying cognitive flexibility and assess their generalizability across tasks. We replicated the robust finding that cognitive flexibility engages distinct neural oscillatory patterns: frontal theta increases reflect task-specific control processes, while parietal alpha/beta suppression represents domain-general attentional mechanisms. Critically, we show that only alpha/beta effects generalized across tasks, supporting their role as neural markers of trait-like flexibility. These findings advance our theoretical understanding of flexibility by demonstrating that it involves sustained attentional processes spanning from cue presentation through target processing.

We first discuss the general oscillatory response to stimuli in cued switching tasks. Because these general response cannot disentangle basal from flexibility-specific processes, we then examine the condition-specific effects reflecting pure cognitive flexibility processes, and discuss their generalizability across tasks. Finally, we evaluate whether oscillatory responses can distinguish between cue- and target-related cognitive flexibility processes.

### General oscillatory response to task demands

Across all three cued task-switching tasks, we found well-known spectral perturbations in response to the cue and target: a transient theta increase followed by a sustained alpha/beta suppression. This pattern was present in both the lower-demand repeat condition and the higher-demand switch condition. The observed spectral perturbations were most pronounced in the occipital cortex, suggesting a visual cortical origin, consistent with the processing of visual stimuli.

Theta, alpha, and beta oscillations are not tied to single cognitive functions, but rather support a range of processes depending on their spectral, spatial, and temporal characteristics^94,95^. In case of visual processing, it has been shown that theta, alpha/beta, and gamma frequency bands function as channels, which route information between visual areas^96,97^. These studies find the same time-frequency patterns as we have observed here and have furthermore shown that theta and gamma frequencies are associated with feedforward and alpha/beta frequencies are associated with feedback influences. Activity within these frequencies may reflect the reallocation of attention by feedforward and feedback loops, possibly ensuring that the task can be completed successfully^98^.

### Condition-specific effects: flexibility-related neural modulations

To isolate flexibility-specific processes, we contrasted switch versus repeat trials. This revealed two distinct oscillatory signatures in the theta and alpha/beta frequency ranges. These signatures differed from the general oscillatory response in terms of their spatial and temporal characteristics, and their generalizability across tasks.

#### Theta as task-specific cognitive control

This contrast revealed a significantly stronger theta power increase in frontal regions during switch trials in response to the cue. The frontal theta enhancement can be linked to a large body of evidence associating mid-frontal theta activity with cognitive control processes, such as conflict monitoring, task-set updating, and rule switching^23,99^. Our findings thus replicate previous findings (e.g., refs. ^22^,^20^) and support the notion that cognitive control is exerted to a greater extent in switch trials than in repeat trials.

However, we should emphasize that we did not observe strictly mid-frontal theta effects. In our switching tasks, we observed effects that were more right-frontal than mid-frontal. Notably, the temporal and spatial maximums of these effects differed between tasks. Nevertheless, the stronger cue-related theta increase in switch trials compared to repeat trials was present in all three shifting tasks. While mid-frontal theta activity is typically associated with cognitive control processes and is thought to originate primarily from midcingulate cortex^99^, the right lateral prefrontal cortex has also been strongly implicated in cognitive control, particularly in response inhibition^100^. According to Ridderinkhof et al.^101^, mid-frontal control signals are translated into action plans in the lateral prefrontal regions. Supporting this view, Verbruggen et al.^102^ experimentally showed that the right ventral inferior frontal cortex is associated with updating action plans. Further evidence for the involvement of lateral prefrontal regions in executive functions comes from a meta-analysis on working memory tasks, requiring some sort of executive control. This analysis revealed that mental manipulations, such as updating or reordering information, are consistently associated with increased activation in right lateral prefrontal cortex ^103^. However, there is also meta-analytic evidence questioning the specific involvement of the lateral frontal cortex in switching, linking this region primarily with executive working memory^104^.

Regarding the target, we found a greater theta increase in switch trials only for the parity/magnitude task. The task-specific nature of theta effects likely reflects the distinct cognitive demands across switching tasks. The number/letter and global/local tasks allow preparatory filtering of relevant information^105^. In contrast, the parity/magnitude task requires reactive control to resolve conflicts arising from the automatic processing of both stimulus categories. This explains why only the parity/magnitude task showed significant target-related theta effects: Interference must be resolved when the target appears, necessitating continued cognitive control.

The lack of cross-task generalizability may also suggest that other parameters than power—for example theta connectivity—underlie cognitive flexibility. This is supported by McKewen et al.^106^, who showed that trial-specific theta connectivity patterns within the fronto-parietal network prepare the cognitive system for the impending need to switch or repeat. Moreover, our own work on this dataset confirms that fronto-parietal theta connectivity robustly indexes flexibility across tasks^32^.

#### Alpha/beta as domain-general attention

Switch trials also showed stronger alpha/beta suppression in comparison to repeat trials, maximal in the left parietal association cortex. This suppression occurred after the initial occipital response and persisted from cue through target processing. Alpha/beta suppression indicates attentional resource allocation^25,26^.

The left parietal focus of this condition-specific alpha/beta effect underscores its role in attentional control and selective attention^107,108^. Notably, this top-down attentional modulation was not limited to the initial cue processing, as around target onset, stronger left parietal alpha/beta suppression was observed in switch trials compared to repeat trials, mirroring the effect seen for the cue. Given that condition differences emerged several hundred milliseconds before target onset and persisted for an equivalent duration after onset, it is reasonable to assume that subjects maintain attention for a prolonged time after the cue is presented. This suggests that they still require more attentional resources to process the target stimulus when the rule-set changes between trials.

These dynamics are consistent with the notion that reallocation of attention, elevated working memory demands, and the goal-directed regulation of cognition and behavior engage distributed control systems, including medial frontal and lateral parietal areas^98,104^. Together, these theta and alpha/beta effects support the engagement of fronto-parietal control networks during task switching^109^, with theta reflecting transient control signals and alpha/beta reflecting sustained attentional adjustments.

We have focused our interpretation of theta and alpha/beta oscillations on cognitive control and attentional processes, respectively. While there is substantial evidence to support this interpretation, there are also alternative interpretations^110^. For example, the widespread alpha/beta effect, may reflect brain-wide reconfiguration processes of neuronal networks, in order to meet the task-demands^111^. Alpha suppression, in particular, is indicative of an activation of the respective region^112^. However, alpha oscillations are not merely a marker of cortical idling. Instead, they actively shape cognition by modulating the excitability of specific neuronal populations^113^, thereby selectively activating or inhibiting particular cortical regions^98^. While alternative interpretations of oscillatory dynamics exist, they converge on the necessity of neural reconfiguration to meet task demands. In the case of cued task-switching, this implies the reallocation of attentional weights and the maintenance of task-relevant neural networks from cue onset through target processing^39^.

### Cross-task generalizability of neural flexibility markers

A main goal of our analyses was to establish whether individual differences in the observed condition-specific effects reflect cognitive flexibility beyond task-specific patterns. We addressed this using structural equation modeling to extract latent factors representing shared variance across switching tasks.

Frontal theta effects, despite being present in all tasks, showed no significant shared variance across tasks. The comparison with a constrained model, were the variance of flexibility was fixed to zero, provided moderate evidence that while cognitive control is engaged universally, the specific control mechanisms are tailored to each task’s demands. Each switching task requires different forms of attention shifting–between stimulus dimensions (parity/magnitude), between perceptual levels (global/local), or between stimuli (number/letter).

In contrast, alpha/beta suppression exhibited significant common variance across all tasks for both cue- and target-related activity. This provides evidence for cognitive flexibility as a latent construct that generalizes beyond task-specific demands. The emergence of this common factor validates the convergent validity of these tasks as measures of cognitive flexibility.

In a second step, we combined the two models of cue- and target-related flexibility, both of which were based on oscillatory alpha/beta band activity, into a single model. In addition, we added two other broad performance constructs, namely, intelligence and WMC, in order to relate cognitive flexibility to these. The analysis revealed that cue- and target-related cognitive flexibility reflect a highly overlapping process, sharing 96% of their variance, suggesting they index the same underlying construct. In addition, alpha/beta suppression in the baseline (repeat) condition was positively correlated across cue- and target-related measures, sharing 53% of the variance.

However, neither flexibility factor correlated significantly with intelligence or WMC. This is consistent with previous findings of a dissociation between flexibility and general cognitive ability^15,114^. Nevertheless, empirical findings on the relationship between flexibility, intelligence, and WMC are inconsistent and may furthermore depend on the specific neuronal or behavioral indicators used^75^. Importantly, we have not explicitly tested the null hypothesis of an independence between the latent constructs. The confidence intervals of the estimated correlations do not exclude the existence of small or even medium effects (cf. Supplementary Table S3).

Baseline alpha/beta suppression (repeat condition) showed weak negative correlations with intelligence and WMC, consistent with the broader literature on alpha suppression and cognitive abilities^115^. Importantly, flexibility measures were independent of baseline suppression, indicating that individual differences in flexibility are distinct from general processing efficiency.

We further showed that cognitive flexibility can be captured via process-based behavioral indicators. Yet, these and ERSP-derived neural markers reflect distinct facets of cognitive flexibility. One interpretation is that alpha/beta suppression captures preparatory attentional reconfiguration, which enables but does not fully determine behavioral performance. Crucially, at the inter-individual level, the magnitude of alpha/beta suppression in response to the cue was linked to switch costs in non-decision time: the stronger the suppression, the less strong the prolongation of the non-decision interval in switch vs. repeat trials. This suggests that the effectiveness of task preparation in switch trials may depend on the degree of alpha/beta suppression elicited by the cue^91,92^. The drift rate, by contrast, reflects the cumulative efficiency of evidence accumulation during target classification, a process shaped by additional factors such as stimulus–response retrieval and response selection that are not uniquely engaged by switching. This dissociation maps onto the diffusion-model decomposition of task-switching costs, in which non-decision time has been tied to anticipatory task-set reconfiguration and drift rate to carry-over from the previously active task set^91,116^. That a preparatory neural marker, cue-locked alpha/beta suppression, tracked the non-decision-time component of flexibility but not the drift-rate component is consistent with this parameter-specific division of labor. We note, however, that this parameter mapping was established through experimental manipulation at the group level, whereas the present association concerns individual differences; the two levels need not converge, so our *t*_0_-specific result should be read as convergent rather than confirmatory. Alternatively, the dissociation may reflect differences in measurement granularity: oscillatory dynamics index moment-to-moment neural states with high temporal precision, whereas drift rates aggregate over the entire decision process, potentially diluting flexibility-specific variance. A similar pattern has been observed for theta connectivity in this dataset, which correlated with general cognitive abilities but not with behavioral switch costs ^32^, reinforcing the broader conclusion that neural signatures of cognitive control are not merely neural translations of behavioral effects. These findings caution against treating neural and behavioral flexibility as interchangeable operationalizations of the same construct. Instead, they point toward a multi-component architecture in which cognitive flexibility emerges from dissociable processes–some reflected in oscillatory dynamics, others in behavioral output–that may jointly but not redundantly contribute to adaptive behavior.

Task switching is theorized to involve an ongoing, adaptive interplay between maintaining task stability and allowing for flexibility^117^. Drift rate has been associated with task-set inertia, i.e., the strength of the activation of the task-set in the previous trial^91,118^. Notably, this association is driven by the repeat trials. Our findings of correlations between alpha/beta suppression and drift rate in the repeat trials are consistent with this interpretation. Since alpha/beta suppression begins as soon as the cue is presented, and target-related alpha/beta suppression occurs even before the target appears, it stands to reason that alpha/beta suppression is temporally positioned to support more effective evidence accumulation. Critically, alternative explanations to task-set inertia cannot be discounted and require explicit experimental testing (e.g., by varying the response-cue interval). Generally, interpretations of these correlations are difficult as long as no measures have been taken to account for the basic cognitive processes, that are also present in the repeat trials^5^.

As in our main analyses, we find that neuronally based flexibility and repeat factors are not substantially correlated. In contrast, for behavioral flexibility we find that participants with a longer non-decision interval and slower drift rate in the repeat condition show smaller switch costs. This could reflect a genuine trade-off between speed and flexibility, indicating that individuals with an overall more controlled and cautious approach demonstrate improved cognitive flexibility at the cost of a general behavioral slowing. This trade-off has been found in mammals^119^, but has not been studied in humans much to date. However, other explanations are also possible, e.g., proportional effects^120^. Although such effects are minimized by the latent change model, they are not eliminated completely.

Taken together, we find preliminary evidence that alpha/beta suppression is attributable to a general flexibility factor that appears to be independent from other performance measures, such as intelligence and WMC. A central contribution of the present study lies in the extraction of common variance across three cued switching tasks, enabling us to identify whether a shared latent process underlies the observed spectral perturbation patterns. While a considerable portion of variance in the neural data is accounted for by task-specific effects, our results provide evidence that cognitive flexibility can be modeled as a latent process that generalizes across distinct tasks.

### Disentangling cue- and target-related processes

The high correlation between cue- and target-related flexibility factors raises questions about whether they represent distinct processes. Several lines of evidence suggest they reflect a single, sustained attentional process rather than separate mechanisms.

Both effects occurred in the same frequency range and location. Critically, cue-related suppression persisted until analysis epoch end, while target-related effects began several hundred milliseconds before target onset. This temporal overlap indicates that attention reallocation initiated by the cue continues through target processing, rather than representing separate preparatory and execution phases.

This pattern supports two-stage theories of task switching^39^, which propose that cues trigger goal-shifting and rule retrieval, while targets require stimulus-response mapping. Our findings suggest that attentional processes initiated during goal-shifting must be maintained through target processing, explaining why residual switch costs persist even with preparation time.

From a neural network perspective, this sustained attention likely reflects the need to maintain activation of task-relevant networks while suppressing competing rule sets^121^. Repeat trials benefit from pre-activated neural patterns representing the current rule set, while switch trials require active reconfiguration of network states, which is a process that cannot be completed before target appearance^39,122^.

Conversely, switch and repeat trials do not differ in terms of the need to activate the correct stimulus-response mapping and retrieve the appropriate motor plan. However, the ease with which this happens should depend on the pre-activation of rule sets and stimulus-response mappings, and therefore on trial type^122,123^. In line with this, we find no evidence that the categorization of the target activates different processes in case of repeating or switching.

In summary, our findings suggest that upon presentation of the cue, more cognitive resources are needed when participants have to switch the current rule-set in comparison when repeating it. This occurs due to stronger alpha/beta suppression in switch trials. The increased activation continued until the presentation of the target and beyond. This indicates that attention allocation and the adaption of attentional weights of relevant networks was started by the cue and endured until the target occurred^67^.

### Limitations

While the present study advances our understanding of the neural underpinnings of cognitive flexibility, it also has some limitations. Our aim of examining the spectral, spatial, and temporal specificity of the oscillatory effects underlying cognitive flexibility was partially compromised by our focus on the 3–60 Hz frequency range, excluding higher gamma frequencies (>60 Hz). Gamma activity, in particular, reflects localized neural network activations^94,124^ and may benefit from high-density EEG recordings. With only 32 electrodes, our study lacks the spatial resolution necessary to precisely localize effects, especially for task-specific phenomena like the cue-related theta ERSP. Future research should employ high-density recordings optimized for gamma analysis and a high spatial resolution. Despite these limitations, our data-driven approach confirmed previous findings and emphasizes the importance of theta and alpha/beta, underscoring the robustness of these bands in cognitive flexibility.

Our task design, which uses central stimuli, and our analysis approach, which allows for whole-brain clusters, is not well suited to capturing fine-grained, lateralized neuronal patterns. Since the left and right hemispheres support different cognitive processes, examining lateralized effects could provide further insight into the attentional processes underlying cognitive flexibility^125^. These effects may be particularly relevant in research on individual differences, since people may vary in their hemispheric specialization. Our approach also overlooks individual differences in spectral and temporal oscillatory patterns. For example the peak frequency of theta or alpha varies between individuals (e.g., ref. ^126^). Examining these variations could reveal neuronal signatures that are missed by mass-univariate cluster tests.

Cluster-based permutation testing, while controlling false positives, may reduce sensitivity to smaller effects^127^. Complementary hypothesis-driven analyses and advanced methods like threshold-free cluster enhancement^128^ could increase sensitivity while maintaining statistical rigor. Furthermore, it is essential to keep in mind that cluster-based permutation tests are based on probabilistic test statistics (here *t*-tests). Consequently, the precise localization of temporal, spectral, and spatial cluster extent is not feasible^73^.

Furthermore, although we employed three distinct tasks to assess cognitive flexibility and are able to generalize from task-specific demands to the abstract construct of flexibility, future work should investigate whether these findings are specific for flexibility. Previous research has shown that an oscillatory response in the form of a transient theta increase and sustained alpha/beta suppression is not unique to flexibility tasks, but rather is involved in general conscious, goal-directed processing (e.g., ref. ^129^).

Additionally, our assessment of cognitive flexibility was limited to the task-switching paradigm with 50% switch probability. While this operationalization is well-established in within executive function models^130^, it may not fully encompass broader or more ecologically valid forms of cognitive flexibility. In clinical research and practice, for example, cognitive flexibility is primarily assessed via the Wisconsin Card Sorting Test^131^. Thus, our operationalization reflects the ability to perform goal-directed set shifting rather than flexibility as a general, multifaceted executive function.

### Conclusion

This study advances our understanding of cognitive flexibility’s neural foundations through several key contributions. We identified and confirmed oscillatory patterns linked to cognitive flexibility. We identified alpha/beta suppression as a promising candidate for a neural marker of domain-general flexibility awaiting further validation, while demonstrating that theta effects reflect task-specific control processes. The methodological framework employed, combining data-driven time-frequency analyses with structural equation modeling, not only increases confidence in the robustness of previous findings but also refines them. Building on the assumption that neuronal communication relies on frequency-specific channels, which are influenced by structural and spatial network properties, we found support for the notion that lower frequencies are associated with top-down attention control, highlighting their importance for flexible, goal-directed behavior. The sustained nature of flexibility-related alpha/beta suppression provides further evidence for two-stage models of task switching, showing that attentional processes must remain active from cue through target processing.

These insights have profound implications for understanding individual differences in cognitive flexibility and its independence from general intelligence. By identifying alpha/beta suppression as a potential neural signature of domain-general flexibility, this work provides a neurobiological foundation for cognitive flexibility as a distinct construct. This framework facilitates the assessment of flexibility capacity, the development of targeted interventions, and the understanding of why some individuals excel at adapting to changing demands while others struggle. Most importantly, our findings demonstrate that cognitive flexibility-a capacity fundamental to learning, problem-solving, and adaptive behavior-can be measured and understood at the neural level. This represents a crucial step toward bridging the gap between cognitive theory and neurobiology, establishing that the brain’s oscillatory dynamics provide a window into one of our most essential cognitive abilities.

Future studies should extend these findings by using broader cognitive batteries to determine if alpha/beta suppression, as a domain-general attentional process, generalizes beyond cued task-switching. Additionally, replication studies should address the limitations of these analyses to determine if alpha/beta dynamics can serve as a robust neural marker of cognitive flexibility across populations and paradigms.

## Supplementary information

Transparent Peer Review file

Supplementary Information

## Data Availability

Raw data are available via OSF (https://osf.io/4pvz3) and pre-processed data via OSF (https://osf.io/aqeph/) or via Github (https://github.com/mhuelsemann/flexibility_TFA).

## Code availability

Analysis code is accessible via OSF (https://osf.io/aqeph/) or via Github (https://github.com/mhuelsemann/flexibility_TFA).
