# Supplementary material for: Task-specific theta enhancement and domain-general alpha/beta suppression as oscillatory signatures of cognitive flexibility
Source: Commun Psychol. 2026 Oct 15;4:140. doi: 10.1038/s44271-026-00521-9 (PMC13672302; doi:10.1038/s44271-026-00521-9)
Supplement: Supplementary file 1 [file 44271_2026_521_MOESM1_ESM.pdf]

nature portfolio

Peer Review File

Task-specific theta enhancement and domain-general alpha/beta suppression as oscillatory signatures of cognitive flexibility

Corresponding Author: Dr Mareike Hülsemann

This file contains all editorial decision letters in order by version, followed by all author rebuttals in order by version.

Version 0:

Decision Letter:

** Please ensure you delete the link to your author homepage in this e-mail if you wish to forward it to your coauthors **

Dear Dr Hülsemann,

Thank you for your patience during the peer-review process. Your manuscript titled "Task-specific theta enhancement and domain-general alpha/beta suppression as oscillatory signatures of individual differences in cognitive flexibility" has now been seen by 3 reviewers, and I include their comments at the end of this message. They find your work of interest but raised some important points. We are interested in the possibility of publishing your study in Communications Psychology, but would like to consider your responses to these concerns and assess a revised manuscript before we make a final decision on publication.

We therefore invite you to revise and resubmit your manuscript, along with a point-by-point response to the reviewers. Please highlight all changes in the manuscript text file.

Editorially, we consider it crucial that you clarify how your research confirms, contradicts, or extends the existing literature and the previous studies using the same dataset. Please also the construct cognitive flexibility is clearly described, and that the methodological details are clarified in the revised manuscript.

Please ensure you follow our statistical guidelines when reporting statistics (https://www.nature.com/commspsychol/submit/submission-guidelines#statistical-guidelines). Please note in particular our requirements for the reporting and interpretation of null-results. Non-significant findings derived from null-hypotheses significance tests should be reported in full, but may not be interpreted. Where you interpret null results, this interpretation must be based on Bayes Factors or equivalence tests.

As you revise the manuscript in response to these issues, please also implement all requests in the attached Mandatory Revision Requests document. All requirements listed in this document need to be fully met, or the work will be returned to you for further revisions without peer review. This workflow is in place to increase the likelihood that the paper will be accepted for publication. It reduces the number of rounds of revision (and review) and ensures that the reviewers vet a version of the article that is compliant with journal policies. If you have any questions regarding the required revisions, please contact the journal prior to resubmission to avoid a negative outcome.

We are committed to providing a fair and constructive peer-review process. Please don't hesitate to contact us if you wish to discuss the revision in more detail.

Please submit the following items:

- Revised manuscript
- Point-by-point response to the referees' comments

- Mandatory Revision Requests Table (attached).
- Cover letter (as a separate document)

via this link: Link Redacted .

** This url links to your confidential home page and associated information about manuscripts you may have submitted or are reviewing for us. If you wish to forward this email to co-authors, please delete the link to your homepage first **

We hope to receive your revised paper within 8 weeks; please let us know if you aren't able to submit it within this time so that we can discuss how best to proceed. If we don't hear from you, and the revision process takes significantly longer, we may close your file. In this event, we will still be happy to reconsider your paper at a later date, provided it still presents a significant contribution to the literature at that stage.

We look forward to seeing the revised manuscript and thank you for the opportunity to review your work.

Best regards,

Troby Lui

Troby Lui, PhD
Associate Editor
Communications Psychology

REVIEWER EXPERTISE:
Reviewer #1: cognitive flexibility, EEG
Reviewer #2: individual differences, cognitive flexibility, EEG
Reviewer #3: individual differences, SEM

REVIEWER REPORTS:

Reviewer #1 (Remarks to the Author):

In this report, the authors use a large sample of participants who completed EEG assessment during a suite of task switching tasks used to assess cognitive flexibility. The authors describe how theta was associated with task-specific control processes (echoing a large literature) and alpha/beta was domain general and attention-related. Some trait-like individual differences were correlated with some of the alpha/beta effects, but these were not related to the derived 'flexibility' measure. In total, while many aspects of the current study were well executed, there questions remain about the nature of some of these cognitive constructs, the interpretations of physiological findings in a data-driven study, and the overall contribution of these findings to the field.

Major Points

1) The construct of cognitive flexibility is widely referred to but under-described. It appears to be the residual variance in EEG data after assessing the slope of the switch vs. stay conditions (pg. 9 lines 634-637). So what is this measure, and why is it particularly appropriate for assessing individual differences in cognitive flexibility?

2) This is a data-driven study, which was indeed executed well. However there is a lack of theoretical insight throughout, which fails to integrate this paper within the broader literature and previous findings relevant to the topic. These are glaring missed opportunities. For some brief examples:
a) Pg. 2 lines 83-87: there are many papers detailing the predictive power of frontal theta on behavioral adaptation
b) Pg. 12 lines 814-816: post hoc reasoning for this decision, which may be theoretically predicted if the signal of the need for cognitive control was effective on the cue and thus does not need to be implemented on the target.
c) Pg. 13 lines 908-910: does this description of theta and alpha/beta effects offer anything new to the field that isn't already well described in theory or in practice?
d) In general, the approach used here is unable to explain non-standard effects (e.g. lateralized effects of theta and alpha), which again diminishes the utility and impact of these findings.
e) How do the authors interpret theta as control and alpha/beta as attention? These again seem to be leveraging the minimum amount of theoretical insight simply for post-hoc explanation, which diminishes the ability to infer the functions of observed physiological processes.

3) Although three tasks provide considerable within-domain assessment of reliability, the specific construct of cognitive flexibility is assessed by the single cognitive function of task switching (all on 50% switch probability tasks). This is less

generalizable to broader executive function than implied throughout the manuscript.

Minor points

1) Fig 2 topos are too small and too monochromatic to observe differences

2) Pg 10 lines 758-766: why is theta described here but not statistically tested? Is this simply a description of non-significant patterns? This is fine, but it should be explicit.

Reviewer #2 (Remarks to the Author):

This paper presents a thorough investigation of which oscillatory markers of task switching can be considered task-specific and which task-general, as well as which can be used for meaningful individual difference research. I reviewed this paper together with a senior grad student in the lab. We both thought this was an interesting study that will be of value to the field. The paper seems methodologically sound, and the results are clear. However, we have two broader, somewhat interrelated concerns that it is unclear how (1) the current findings relate to the several previous papers on this same dataset, and (2) these findings relate to behavior. In more detail:

While we thought the study was surely interesting, and introduced well, we were surprised to read in the method section that the authors already analyzed this dataset in nine previous publications. Critically, several of these seem very relevant, but their findings are not discussed in the paper. Given that the current study is part of an extensive program using this dataset, we believe readers need a clearer map showing how the present findings align or depart from earlier conclusions. For example, previous papers on this same dataset that are clearly relevant are:

Schubert, A. L., Löffler, C., Jungeblut, H. M., & Hülsemann, M. J. (2025). Trait characteristics of midfrontal theta connectivity as a neurocognitive measure of cognitive control and its relation to general cognitive abilities. Journal of Experimental Psychology: General.

Löffler, C., Sadus, K., Frischkorn, G. T., Hagemann, D., & Schubert, A. L. (2025). The factor structure of executive functions measured with electrophysiological correlates: An event-related potential analysis. Journal of Experimental Psychology: Learning, Memory, and Cognition.

Schubert, A. L., Löffler, C., & Hagemann, D. (2022). A neurocognitive psychometrics account of individual differences in attentional control. Journal of Experimental Psychology: General, 151(9), 2060.

Schubert, A. L., Löffler, C., Hagemann, D., & Sadus, K. (2023). How robust is the relationship between neural processing speed and cognitive abilities?. Psychophysiology, 60(2), e14165.

Löffler, C., Frischkorn, G. T., Hagemann, D., Sadus, K., & Schubert, A. L. (2024). The common factor of executive functions measures nothing but speed of information uptake. Psychological research, 88(4), 1092-1114.

For example, in their opening paragraph, the current paper takes as a starting point the three-factor structure by Miyake and colleagues (2000), without raising any caveats to this model. However, the authors of this manuscript previously critically and extensively discussed this model by Miyake and colleagues in Löffler et al. (2024), further raising the many different criticisms that people have brought up against it in other papers, too, and they, themselves, concluded that: “We therefore suggest refraining from using typical executive function tasks to study substantial research questions, as these tasks are not valid for measuring individual differences in executive functions.” in the last sentence of their abstract (Löffler et al., 2024).

As another example, this paper now concludes that in contrast to alpha/beta suppression, “Frontal theta reflected task-specific control processes” (abstract). However, this same year and on the same dataset, Schubert et al. (2025) concluded that “Our findings reveal that midfrontal theta connectivity can be modeled as a trait-like latent variable, indicating its consistency across tasks and stability over time.” (abstract, Schubert et al., 2025).

Of course, these conclusions may ultimately be compatible, and as mentioned above we believe the data analyses and results of the current paper are convincing, but it is clear that these other findings are also relevant to the current study, *especially* because they are based on the same dataset. Therefore, we consider it important to understand the current findings in the broader context of the authors’ other, previous conclusions on both the literature as a whole and this very same dataset.

This brings us to our second point. While these markers seem of use to the field, and the task-specificity of theta versus generality of alpha/beta is an interesting standalone finding, their interpretability would benefit greatly from a clearer relationship to behavior. The current paper now only briefly mentions the standard switch costs in reaction time and accuracy, but does not explore (1) underlying factor structures based on those, (2) relations between the oscillatory signatures and these behavioral markers, or (3) relation with previous DDM markers the authors developed and proposed in a previous publication on this dataset. Such behavioral analyses would be of relevance to several of the questions the authors, themselves, raise in the discussion now (e.g., the relation with transient versus sustained changes in behavior, the

relation with switching versus working memory, the uniqueness to flexibility tasks?).

Given the number of prior papers on this dataset, we would strongly encourage the authors to integrate these behavioral dimensions here rather than postponing them to another manuscript. At this stage of the research program, additional fragmentation of analyses risks obscuring the broader picture, and incorporating these behavioral relations here would substantially improve coherence.

Minor:

We would consider dropping the “of individual differences” in the title. The title right now is a bit on the long side, and while this study sure has important implications for the study of individual differences, we found its implications to extend beyond that.

The results section was now written relatively descriptively. We thought it would be very helpful if the authors could provide a little bit more of a narrative in the results section, interim conclusions, or small summaries.

Line 621, please provide references for the sentence that starts with “In line with previous research, the reliabilities of the …”.

Other relevant papers may be:
Angelidis, A., van der Does, W., Schakel, L., & Putman, P. (2016). Frontal EEG theta/beta ratio as an electrophysiological marker for attentional control and its test-retest reliability. Biological psychology, 121, 49-52.
McKewen, M., Cooper, P. S., Skippen, P., Wong, A. S., Michie, P. T., & Karayanidis, F. (2021). Dissociable theta networks underlie the switch and mixing costs during task switching. Human Brain Mapping, 42(14), 4643-4657.

Reviewer #3 (Remarks to the Author):

I commend the authors for addressing an important issue in the field and adding to pre-existing literature in this vein of work. I have several points of improvement that the authors should address before this piece is ready for publication.

Introduction
- Strengthen the justification for why flexibility should or should not relate to general cognitive abilities by explicitly engaging models such as Process Overlap Theory (Kovacs & Conway, 2016), hierarchical control accounts, and resource-based frameworks.
- Incorporate alternative interpretations of theta and alpha/beta oscillatory functions and clarify why the chosen interpretations best fit the present findings.
- Clarify the theoretical status of alpha/beta suppression— does it index attentional reallocation, cognitive control, or more domain-general feedback processes? Explain how these possibilities relate to flexibility specifically.

Methodology
- Address the impact of relatively high trial/epoch rejection rates on SNR and discuss whether this could bias individual differences estimates.
- Provide justification for relying on latent-change modeling when manifest reliability is near-zero for some indicators; clarify whether the latent construct contains sufficient signal to support trait-level interpretation.
- Discuss whether the lack of generalizability for the theta and beta clusters truly reflects task specificity or could reflect lower cross-task variance or insufficient statistical power?

Statistical Modeling and Interpretation
- Clarify the interpretational implications of the target-related alpha/beta suppression beginning prior to target onset—does this reflect anticipatory processing rather than target-driven flexibility?
- More clearly articulate limitations of the mass-univariate approach, particularly that cluster extent cannot be precisely localized in space or frequency.

Conclusions and Claims
- Reframe claims that flexibility is 'distinct' from intelligence or WMC as preliminary given measurement constraints, range restriction, and modest reliability of some indicators. This conclusion should be qualified at the minimum.
- Temper language suggesting definitive neural markers; emphasize instead that alpha/beta suppression is a promising and replicable candidate but requires further validation.
- Discuss future work explicitly—including improving neural reliability, testing broader cognitive batteries, and conducting replication studies—to position conclusions within a developmental trajectory rather than a definitive endpoint. Just suggestions.

* TRANSPARENT PEER REVIEW: Communications Psychology uses a transparent peer review system. This means that we publish the editorial decision letters including Reviewers' comments to the authors and the author rebuttal letters online

as a supplementary peer review file. However, on author request, confidential information and data can be removed from the published reviewer reports and rebuttal letters prior to publication. If your manuscript has been previously reviewed at another journal, those Reviewers' comments would not form part of the published peer review file.

** Visit Nature Research's author and referees' website at www.nature.com/authors for information about policies, services and author benefits**

Communications Psychology is committed to improving transparency in authorship. As part of our efforts in this direction, we are now requesting that all authors identified as ‘corresponding author’ create and link their Open Researcher and Contributor Identifier (ORCID) with their account on the Manuscript Tracking System prior to acceptance. ORCID helps the scientific community achieve unambiguous attribution of all scholarly contributions. You can create and link your ORCID from the home page of the Manuscript Tracking System by clicking on ‘Modify my Springer Nature account’ and following the instructions in the link below. Please also inform all co-authors that they can add their ORCIDs to their accounts and that they must do so prior to acceptance.
https://www.springernature.com/gp/researchers/orcid/orcid-for-nature-research

For more information please visit http://www.springernature.com/orcid

If you experience problems in linking your ORCID, please contact the Platform Support Helpdesk.

Version 1:

Decision Letter:

** Please ensure you delete the link to your author homepage in this e-mail if you wish to forward it to your coauthors **

Dear Dr Hülsemann,

Thank you for your patience during the peer-review process. Your manuscript titled "Task-specific theta enhancement and domain-general alpha/beta suppression as oscillatory signatures of cognitive flexibility" has now been seen by 4 reviewers, and I include their comments at the end of this message. They find your work of interest but raised some important points. We are interested in the possibility of publishing your study in Communications Psychology, but would like to consider your responses to these concerns and assess a revised manuscript before we make a final decision on publication.

We therefore invite you to revise and resubmit your manuscript, along with a point-by-point response to the reviewers. Please highlight all changes in the manuscript text file.

Editorially, we consider it crucial that reviewer #2’s remaining methodological and theoretical concerns regarding the drift rate analysis are addressed in the revised manuscript.

We are committed to providing a fair and constructive peer-review process. Please don't hesitate to contact us if you wish to discuss the revision in more detail.

Please submit the following items:

- Revised manuscript
- Point-by-point response to the referees’ comments
- Mandatory Revision Requests Table (attached).
- Cover letter (as a separate document)

via this link: Link Redacted .

** This url links to your confidential home page and associated information about manuscripts you may have submitted or are reviewing for us. If you wish to forward this email to co-authors, please delete the link to your homepage first **

We hope to receive your revised paper within 8 weeks; please let us know if you aren’t able to submit it within this time so that we can discuss how best to proceed. If we don’t hear from you, and the revision process takes significantly longer, we may close your file. In this event, we will still be happy to reconsider your paper at a later date, provided it still presents a significant contribution to the literature at that stage.

We look forward to seeing the revised manuscript and thank you for the opportunity to review your work.

Best regards,

Troby Lui

Troby Lui, PhD
Associate Editor
Communications Psychology

REVIEWER REPORTS:

Reviewer #1 (Remarks to the Author):

The authors have been responsive in this revision: both to my previous concerns as well as to issues raised by other reviewers. Yet, notably, most of my concerns were not procedural or identifying issues with specific choices: they were examples of cases where this data-driven analysis and post-hoc interpretation failed to lead to novel theoretical conclusions. This manuscript remains a technical tour-de-force, but I think it will have only a minor impact on the field due to raising more questions than it answers.

Reviewer #2 (Remarks to the Author):

We would like to thank the authors for responding to our comments, providing extra analyses, and revising their manuscript. We believe the manuscript has certainly improved. However, one of the new additions was to show a relation with behavior for which the authors now provide analyses that link drift diffusion measures to their oscillatory measures of EEG activity. We thought those new analyses were interesting, but believe some important analysis details are missing and the results could be better related to broader theories of task switching. As this already concerns a revised manuscript, we tried to keep our comments targeted:

First, we were wondering how different parameters were estimated. Specifically, for which experimental conditions were the individual parameters estimated separately versus shared? The paper refers to Löffler et al. for further details, but we could not immediately find those details there either, and believe these should be repeated in the main manuscript. From the results section, we inferred that the authors must have estimated separate drift rates for task repetitions and task switches? However, did they also do this for non-decision times? The latter would be important as previous studies using DDM in task switching usually rely on the distinction between those two, with differences in drift rates been argued to reflect carry-over effects of the previous task and non-decision time a reconfiguration process (e.g., see Hartanto & Yang, 2016; Imburgio & Orr, 2021; Schmitz & Voss, 2012; 2014). Therefore, we would be interested in seeing the results for relations with switch- versus repetition-related non-decision times, too.

Second, the authors seem to emphasize that their relations with behavior are not related to task switching, and reflect distinct dimensions, because the brain-behavior correlations are driven by task repetition trials. However, classic and contemporary models of cognitive control suggest that task switching is a dynamic negotiation between task stability and flexibility (Allport et al., 1994; Gilbert & Shallice, 2002; Xu, Verguts, & Braem, 2026). In these frameworks, the drift rate on repetitions is a direct proxy for the "strength" or "inertia" of a task set. If the authors' oscillatory measure predicts drift rates on repetitions, it may be capturing the neural signature of task-set persistence, the very state that must be overcome during a switch? Therefore, the authors could consider discussing their findings in the context of models where flexibility is the emergent product of both inertia and reconfiguration?

Reviewer #3 (Remarks to the Author):

The paper is now adequate for publication. Thank you to the authors for addressing the comments provided.

Reviewer #4 (Remarks to the Author):

I co-reviewed this manuscript with one of the reviewers who provided the listed reports. This is part of the Communications Psychology initiative to facilitate training in peer review and to provide appropriate recognition for Early Career Researchers who co-review manuscripts.

* TRANSPARENT PEER REVIEW: Communications Psychology uses a transparent peer review system. This means that

we publish the editorial decision letters including Reviewers' comments to the authors and the author rebuttal letters online as a supplementary peer review file. However, on author request, confidential information and data can be removed from the published reviewer reports and rebuttal letters prior to publication. If your manuscript has been previously reviewed at another journal, those Reviewers' comments would not form part of the published peer review file.

** Visit Nature Research's author and referees' website at www.nature.com/authors for information about policies, services and author benefits**

Communications Psychology is committed to improving transparency in authorship. As part of our efforts in this direction, we are now requesting that all authors identified as 'corresponding author' create and link their Open Researcher and Contributor Identifier (ORCID) with their account on the Manuscript Tracking System prior to acceptance. ORCID helps the scientific community achieve unambiguous attribution of all scholarly contributions. You can create and link your ORCID from the home page of the Manuscript Tracking System by clicking on 'Modify my Springer Nature account' and following the instructions in the link below. Please also inform all co-authors that they can add their ORCIDs to their accounts and that they must do so prior to acceptance.
https://www.springernature.com/gp/researchers/orcid/orcid-for-nature-research

For more information please visit http://www.springernature.com/orcid

If you experience problems in linking your ORCID, please contact the Platform Support Helpdesk.

Version 2:

Decision Letter:

** Please ensure you delete the link to your author homepage in this e-mail if you wish to forward it to your coauthors **

Dear Dr Hülsemann,

Your manuscript titled "Task-specific theta enhancement and domain-general alpha/beta suppression as oscillatory signatures of cognitive flexibility" has now been seen by our reviewers, whose comments appear below. In light of their advice I am delighted to say that we are happy, in principle, to publish a suitably revised version in Communications Psychology.

We therefore invite you to revise your paper one last time to address the remaining concerns of our reviewers and a list of editorial requests. At the same time we ask that you edit your manuscript to comply with our format requirements and to maximise the accessibility and therefore the impact of your work.

EDITORIAL REQUESTS:

Please review our specific editorial comments and requests regarding your manuscript in the attached "Editorial Requests Table". Please outline your response to each request in the right hand column. Please upload the completed table with your manuscript files as a Related Manuscript file.

If you have any questions or concerns about any of our requests, please do not hesitate to contact me.

SUBMISSION INFORMATION:

In order to accept your paper, we require the files listed here https://www.nature.com/documents/commsj-file-checklist.pdf .

OPEN ACCESS:

Communications Psychology is a fully open access journal. Articles are made freely accessible on publication. For further information about article processing charges, open access funding, and advice and support from Nature Research, please visit https://www.nature.com/commspsychol/open-access

At acceptance, you will be provided with instructions for completing the open access licence agreement on behalf of all authors. This grants us the necessary permissions to publish your paper. Additionally, you will be asked to declare that all required third party permissions have been obtained, and to provide billing information in order to pay the article-processing charge (APC).

* TRANSPARENT PEER REVIEW: Communications Psychology uses a transparent peer review system. On author request, confidential information and data can be removed from the published reviewer reports and rebuttal letters prior to publication. If you are concerned about the release of confidential data, please let us know specifically what information you would like to have removed. Please note that we cannot incorporate redactions for any other reasons.

* CODE AVAILABILITY: All Communications Psychology manuscripts must include a section titled "Code Availability" at the end of the methods section. We require that the custom analysis code supporting your conclusions is made available in a publicly accessible repository at this stage; please choose a repository that generates a digital object identifier (DOI) for the code; the link to the repository and the DOI must be included in the Code Availability statement. Publication as Supplementary Information will not suffice.

* DATA AVAILABILITY:
All Communications Psychology manuscripts must include a section titled "Data Availability" at the end of the Methods section. More information on this policy, is available in the Editorial Requests Table and at http://www.nature.com/authors/policies/data/data-availability-statements-data-citations.pdf.

Please use the following link to submit the above items:
Link Redacted
** This url links to your confidential home page and associated information about manuscripts you may have submitted or be reviewing for us. If you wish to forward this email to co-authors, please delete the link to your homepage first **

We hope to hear from you within two weeks; please let us know if you need more time.

Best regards,

Troby Lui

Troby Lui, PhD
Associate Editor
Communications Psychology

REVIEWERS' COMMENTS:

Reviewer #2 (Remarks to the Author):

I'd like to thank the authors for considering our comments and suggestions. I think this manuscript has much improved, and believe it is a great contribution to the literature. I have no further suggestions.

Reviewer #4 (Remarks to the Author):

I co-reviewed this manuscript with one of the reviewers who provided the listed reports. This is part of the Communications Psychology initiative to facilitate training in peer review and to provide appropriate recognition for Early Career Researchers who co-review manuscripts.

** Visit Nature Research's author and referees' website at www.nature.com/authors for information about policies, services and author benefits**

Open Access This Peer Review File is licensed under a Creative Commons Attribution 4.0 International License, which permits use, sharing, adaptation, distribution and reproduction in any medium or format, as long as you give appropriate credit to the original author(s) and the source, provide a link to the Creative Commons license, and indicate if changes were made.
In cases where reviewers are anonymous, credit should be given to 'Anonymous Referee' and the source.
The images or other third party material in this Peer Review File are included in the article's Creative Commons license, unless indicated otherwise in a credit line to the material. If material is not included in the article's Creative Commons license and your intended use is not permitted by statutory regulation or exceeds the permitted use, you will need to obtain permission directly from the copyright holder.
To view a copy of this license, visit https://creativecommons.org/licenses/by/4.0/

Response Letter

Reviewer #1 (Remarks to the Author):

In this report, the authors use a large sample of participants who completed EEG assessment during a suite of task switching tasks used to assess cognitive flexibility. The authors describe how theta was associated with task-specific control processes (echoing a large literature) and alpha/beta was domain general and attention-related. Some trait-like individual differences were correlated with some of the alpha/beta effects, but these were not related to the derived 'flexibility' measure. In total, while many aspects of the current study were well executed, there questions remain about the nature of some of these cognitive constructs, the interpretations of physiological findings in a data-driven study, and the overall contribution of these findings to the field.

We thank the Reviewer for the positive evaluation of our work and for pointing out relevant aspects for improving our manuscript. We revised our manuscript to better situate our empirical findings within the existing theoretical context, improve the interpretation of the detected oscillatory responses, and highlight how our results advance previous research. We detail our changes below.

Major Points

1) The construct of cognitive flexibility is widely referred to but under-described. It appears to be the residual variance in EEG data after assessing the slope of the switch vs. stay conditions (pg. 9 lines 634-637). So what is this measure, and why is it particularly appropriate for assessing individual differences in cognitive flexibility?

In the context of the task-switching paradigm, cognitive flexibility is defined as additional cognitive process required during switch trials compared to repeat trials. Traditionally, researchers have measured this by simply subtracting repeat-trial performance from switch-trial performance to obtain a "manifest difference score". But these raw differences are often unreliable — and our data confirm that, too. To overcome this problem, we use a latent change model. The latent change score is conceptually the same as a manifest difference score, but it has the advantage of overcoming the reliability problem of manifest difference scores. The latent change model is particularly appropriate for assessing individual differences in cognitive flexibility, as it estimates true change by combining information from multiple related measurements, rather than directly comparing two unreliable scores. This makes the results more stable, reliable, and meaningful. We now explain this more explicitly:

> *At the manifest level, a difference score is computed by directly subtracting one observed variable from another. The latent change score model is conceptually analogous in that it also captures the difference between two timepoints; however, it does so by modeling change as a latent variable, thereby partitioning observed variance into true score variance and measurement error. Critically, this means the latent change score estimates true change, disentangled from random measurement error, whereas the raw difference score conflates genuine change*

> *with error variance from both measurement occasions — amplifying unreliability rather than canceling it. This is a fundamental psychometric liability of manifest difference scores: because their reliability is bounded by, and typically lower than, the reliability of either constituent measure, they tend to attenuate correlations with external variables and inflate within-person variability spuriously. The latent change score framework circumvents these problems by imposing the measurement model directly, yielding estimates of change that are corrected for attenuation and more accurately reflect the underlying construct of interestn (see Schubert, Löffler, & Hagemann, 2022; Löffler, Sadus, Frischkorn, Hagemann, & Schubert, 2025, for a more detailed explanation). Thereby, the latent difference overcomes the psychometric problems that are associated with manifest difference scores.* (pages 9-10, lines 697-723)

2) This is a data-driven study, which was indeed executed well. However there is a lack of theoretical insight throughout, which fails to integrate this paper within the broader literature and previous findings relevant to the topic. These are glaring missed opportunities. For some brief examples:

We thank the reviewer for the positive assessment of the data-driven aspects of our work and, most importantly, for drawing our attention to the need for a stronger theoretical framing. We revised our manuscript to include and discuss more relevant literature. We believe that we have now included clear statements detailing how the presented analyses confirm, contradict, and extend previous relevant findings. The changes we made are detailed below.

a) Pg. 2 lines 83-87: there are many papers detailing the predictive power of frontal theta on behavioral adaptation

Our statement on p. 2 aimed to draw attention to a gap in the literature: despite substantial evidence supporting the predictive power of frontal theta on goal-directed behavior, little research has examined how individual differences in theta power relate to cognitive flexibility specifically. However, to acknowledge previous research more prominently, we now included two well-executed exemplary studies that demonstrate how frontal theta power predicts goal-directed behavior in the context of rule-switching and response conflict:

> *Although experimental work shows that oscillatory dynamics, such as theta power, are causally involved in flexible rule-switching (Zhang, Deng, Liao, Verguts, & Chen, 2026) and can predict individual differences in goal-directed behavior (e.g., response conflict: Vissers, Ridderinkhof, Cohen, & Slagter, 2018), the extent to which they reliably index trait-level cognitive-flexibility has not yet been demonstrated.* (page 3, lines 125-133)

b) Pg. 12 lines 814-816: post hoc reasoning for this decision, which may be theoretically predicted if the signal of the need for cognitive control was effective on the cue and thus does not need to be implemented on the target.

We understand the concern of the reviewer and agree that the appearance of this cluster could have been predicted from the findings of the cue-related activity. The exclusion of the early cluster is a direct consequence of our a priori analysis design: In our design, the baseline epoch precedes the target by a large interval during which the cue is presented and processed. Consequently, cue-related activity inevitably appears in the target epoch. However, since we separately analyzed cue-related dynamics using cue-aligned epochs, the observed cluster emerging before target onset is best interpreted as a methodological artifact of the extended analysis window, not a genuine target-related effect. It was necessary to choose such a large preceding interval, to obtain a pre-target baseline that is expected to be largely free of task-related neural activity. In order to make the reasoning for not interpreting these clusters more transparent, we added the following explanation:

> The clear temporal onset before the target in the number/letter task suggests a methodological artifact. *Because the target epoch contains a pre-cue baseline and the cue presentation period, cue-related activity inevitably appears in the target window, rendering the early theta increase a methodological artifact rather than a genuine target-related effect. Since cue-related dynamics were already modeled separately, this cluster was omitted from further analyses of cognitive flexibility.* (p. 14 lines 941-947)

c) Pg. 13 lines 908-910: does this description of theta and alpha/beta effects offer anything new to the field that isn't already well described in theory or in practice?

The finding of an enhanced theta increase and an enhanced alpha/beta suppression in switch vs. repeat trials is a robust finding across laboratories and tasks. We replicate these findings and extend them by showing that only alpha/beta suppression, but not theta increases, generalize across different flexibility tasks. We reworded the respective paragraph, to clarify which of our findings are replications and which are new insights:

> Our main goal was to identify oscillatory mechanisms underlying cognitive flexibility and assess their generalizability across tasks. We *replicated the robust finding* that cognitive flexibility engages distinct neural oscillatory patterns: frontal theta increases reflect task-specific control processes, while parietal alpha/beta suppression represents domain-general attentional mechanisms. Critically, *we show that* only alpha/beta effects generalized across tasks, supporting their role as neural markers of trait-like flexibility. These findings advance our theoretical understanding of flexibility by demonstrating that it involves sustained attentional processes spanning from cue presentation through target processing. (p. 16, lines 1107-1108 and 1112)

d) In general, the approach used here is unable to explain non-standard effects (e.g. lateralized effects of theta and alpha), which again diminishes the utility and impact of these findings.

Indeed, in the present analysis we focus specifically on event–related power modulations in a broad frequency range of 3 to 60 Hz. As such, it is not designed to capture or explain

lateralized or other non-standard effects. We fully agree that this represents a limitation of the current study, and we now explicitly acknowledge this in the Discussion section to provide a more balanced interpretation of our findings:

> *Our task design, which uses central stimuli, and our analysis approach, which allows for whole-brain clusters, is not well suited to capturing fine-grained, lateralized neuronal patterns. Since the left and right hemispheres support different cognitive processes, examining lateralized effects could provide further insight into the attentional processes underlying cognitive flexibility (Harris, Dux, Jones, & Mattingley, 2017). These effects may be particularly relevant in research on individual differences, since people may vary in their hemispheric specialization. Our approach also overlooks individual differences in spectral and temporal oscillatory patterns. For example the peak frequency of theta or alpha varies between individuals (e.g. Haegens, Cousijn, Wallis, Harrison, & Nobre, 2014). Examining these variations could reveal neuronal signatures that are missed by mass-univariate cluster tests.* (p. 21, lines 1466-1482)

e) How do the authors interpret theta as control and alpha/beta as attention? These again seem to be leveraging the minimum amount of theoretical insight simply for post-hoc explanation, which diminishes the ability to infer the functions of observed physiological processes.

Having conducted a data-driven approach, our manuscript indeed relies more strongly on post-hoc explanations for the functional interpretation of the detected frequency bands than we would have in a hypothesis-driven approach. The present approach did not aim so much at inferring the functions of observed physiological processes, but rather focused on whether detected physiological processes are generalizable and differentially related to the construct under investigation. We understand that this is an limitation of our work, which we should address more clearly. We rely strongly on the large literature of frontal theta as marker for cognitive control (Cavanagh & Frank, 2014; Cavanagh & Shackman, 2015). Regarding the alpha/beta oscillations, the interpretation is not so straight-forward. It is important to acknowledge the difficulty of establishing a one-to-one mapping between specific frequency bands and cognitive processes. This is due to the fact that the number of identifiable cognitive functions far exceeds the number of frequency bands. Instead, it is more plausible to assume that neuronal communication is organized via frequency-specific channels, where the exact frequency used depends on the structural and spatial properties of the interacting neural networks (Buzsáki, 2006; Vezoli et al., 2021). We discuss this aspect briefly, at the beginning of our Discussion (page 17, lines 1138-1154) and have further added the following paragraph to our Discussion, to better ground our interpretations of theta as cognitive control marker and alpha/beta as attention marker:

> *We have focused our interpretation of theta and alpha/beta oscillations on cognitive control and attentional processes, respectively. While there is substantial evidence to support this interpretation, there are also alternative interpretations (Keitel et al., 2025). For example, the widespread alpha/beta effect, may reflect brain-wide reconfiguration processes of neuronal networks, in order to meet the*

> *task-demands (Gratton, 2018). Alpha suppression, in particular, is indicative of an activation of the respective region (Haegens, Nácher, Luna, Romo, & Jensen, 2011). However, alpha oscillations are not merely a marker of cortical idling. Instead, they actively shape cognition by modulating the excitability of specific neuronal populations (Lange, Oostenveld, & Fries, 2013), thereby selectively activating or inhibiting particular cortical regions (Sadaghiani & Kleinschmidt, 2016). While alternative interpretations of oscillatory dynamics exist, they converge on the necessity of neural reconfiguration to meet task demands. In the case of cued task-switching, this implies the reallocation of attentional weights and the maintenance of task-relevant neural networks from cue onset through target processing (Kiesel et al., 2010).* (pages 18-19, lines 1266-1288)

We would like to mention that this paragraph was not only added to meet this comment, but also to meet a distinct, but related comment of Reviewer #3.

3) Although three tasks provide considerable within-domain assessment of reliability, the specific construct of cognitive flexibility is assessed by the single cognitive function of task switching (all on 50% switch probability tasks). This is less generalizable to broader executive function than implied throughout the manuscript.

We agree that this is a relevant limitation of our study. We incorporate this important restriction to the Limitations section in our Discussion:

> *Additionally, our assessment of cognitive flexibility was limited to the task-switching paradigm with 50 % switch probability. While this operationalization is well-established in within executive function models (Karr et al., 2018), it may not fully encompass broader or more ecologically valid forms of cognitive flexibility. In clinical research and practice, for example, cognitive flexibility is primarily assessed via the Wisconsin Card Sorting Test (Miles et al., 2021). Thus, our operationalization reflects the ability to perform goal-directed set shifting rather than flexibility as a general, multifaceted executive function.*

Minor points

1) Fig 2 topos are too small and too monochromatic to observe differences

We thank the reviewer for pointing this out. We reduced the amount of topoplots to increase their size and changed the monochromatic colormap to the upper half of parula in order to emphasize effect size differences.

2) Pg 10 lines 758-766: why is theta described here but not statistically tested? Is this simply a description of non-significant patterns? This is fine, but it should be explicit.

For cue- and target-related analysis, we first describe the condition-independent spectral response to the respective stimulus (cue, target). The condition-independent general spectral response to cue and target was not statistically tested (e.g. difference to baseline),

as our research question rather concerned condition difference in the spectral response. To make these different analysis steps clearer, we added narrative explanatory sentences in the Results section, as suggested by Reviewer #2. The changes are marked throughout the revised manuscripts (lines 775-776, 787-789, 796, 806, 818-824, 827-829, 831-833, 875-877, 879-882, 898-902, 915-916, 953-954, 971-981, 984-986, 988, 1006-1009, 1047-1049)

Reviewer #2 (Remarks to the Author):

This paper presents a thorough investigation of which oscillatory markers of task switching can be considered task-specific and which task-general, as well as which can be used for meaningful individual difference research. I reviewed this paper together with a senior grad student in the lab. We both thought this was an interesting study that will be of value to the field. The paper seems methodologically sound, and the results are clear. However, we have two broader, somewhat interrelated concerns that it is unclear how (1) the current findings relate to the several previous papers on this same dataset, and (2) these findings relate to behavior. In more detail:

While we thought the study was surely interesting, and introduced well, we were surprised to read in the method section that the authors already analyzed this dataset in nine previous publications. Critically, several of these seem very relevant, but their findings are not discussed in the paper. Given that the current study is part of an extensive program using this dataset, we believe readers need a clearer map showing how the present findings align or depart from earlier conclusions. For example, previous papers on this same dataset that are clearly relevant are:

- Schubert, A. L., Löffler, C., Jungeblut, H. M., & Hülsemann, M. J. (2025). Trait characteristics of midfrontal theta connectivity as a neurocognitive measure of cognitive control and its relation to general cognitive abilities. Journal of Experimental Psychology: General.

- Löffler, C., Sadus, K., Frischkorn, G. T., Hagemann, D., & Schubert, A. L. (2025). The factor structure of executive functions measured with electrophysiological correlates: An event-related potential analysis. Journal of Experimental Psychology: Learning, Memory, and Cognition.

- Schubert, A. L., Löffler, C., & Hagemann, D. (2022). A neurocognitive psychometrics account of individual differences in attentional control. Journal of Experimental Psychology: General, 151(9), 2060.

- Schubert, A. L., Löffler, C., Hagemann, D., & Sadus, K. (2023). How robust is the relationship between neural processing speed and cognitive abilities?. Psychophysiology, 60(2), e14165.

- Löffler, C., Frischkorn, G. T., Hagemann, D., Sadus, K., & Schubert, A. L. (2024). The common factor of executive functions measures nothing but speed of information uptake. Psychological research, 88(4), 1092-1114.

For example, in their opening paragraph, the current paper takes as a starting point the three-factor structure by Miyake and colleagues (2000), without raising any caveats to

this model. However, the authors of this manuscript previously critically and extensively discussed this model by Miyake and colleagues in Löffler et al. (2024), further raising the many different criticisms that people have brought up against it in other papers, too, and they, themselves, concluded that: "We therefore suggest refraining from using typical executive function tasks to study substantial research questions, as these tasks are not valid for measuring individual differences in executive functions." in the last sentence of their abstract (Löffler et al., 2024).

As another example, this paper now concludes that in contrast to alpha/beta suppression, "Frontal theta reflected task-specific control processes" (abstract). However, this same year and on the same dataset, Schubert et al. (2025) concluded that "Our findings reveal that midfrontal theta connectivity can be modeled as a trait-like latent variable, indicating its consistency across tasks and stability over time." (abstract, Schubert et al., 2025).

Of course, these conclusions may ultimately be compatible, and as mentioned above we believe the data analyses and results of the current paper are convincing, but it is clear that these other findings are also relevant to the current study, *especially* because they are based on the same dataset. Therefore, we consider it important to understand the current findings in the broader context of the authors' other, previous conclusions on both the literature as a whole and this very same dataset.

We thank the reviewers for emphasizing the relevance of our group's previous papers and for pointing out potential contradictions between the current analyses and those previous analyses of the same dataset. We hope to clarify these aspects by integrating our previous findings in the revised version of the manuscript. In previous work, we could show that executive functions cannot be captured by behavioral measures nor by the power of even-related potential components (Löffler, Frischkorn, Hagemann, Sadus, & Schubert, 2024; Löffler et al., 2025). However, the inability of established measures to effectively isolate executive functions from classic executive function tasks does not preclude the possibility of measuring executive functions through other indicators in these same tasks. For example, Schubert, Löffler, Jungeblut, and Hülsemann (2025) could show that mid-frontal global theta connectivity is a stable trait-like measure of cognitive flexibility. In the present paper we diverge from previous analyses in two aspects. First, we model cognitive flexibility with a latent change model, which is a markedly different approach to the structural equation models used by our previous analyses. Second, we explore an indepentdent electrophysiological marker, namely event-related spectral perturbations. The latent change approach in combination with an well-established electrophysiological marker in group-level analysis opens the possibility to even find evidence for one of the most influential executive function theories (Miyake et al., 2000). In the present paper, we aim to establish, whether event-related spectral perturbations are suitable to isolate cognitive flexibility from basic cognitive processes and are furthermore a generalizable flexibility measure across different tasks.

We have changed the following sentence in our introduction in order to not leave the criticism expressed about the Miyake model uncommented:

As a core component of *an influential—though contested—executive function*

> *model (Miyake et al., 2000; Löffler et al., 2024)*, alongside inhibition and updating, cognitive flexibility is theorized to underlie more complex goal-directed processes such as planning and problem-solving. (p. 1, lines 11-12)

After careful consideration, we concluded that Schubert et al. (2022) and Schubert, Löffler, Hagemann, and Sadus (2023) address research questions that are largely unrelated to the aims and methodology of the present manuscript. Both suggested papers are valuable within their own domains, but they address different constructs (inhibition vs. flexibility; processing speed vs. flexibility) and employ different neural measures (ERP components (P2, N2, P3, LRP) vs. ERSP). Therefore we think that these papers do not map onto the hypotheses, experimental design, and analytic strategy of the current study. Nevertheless, to establish a clearer link to previous findings from this data set, we acknowledge these papers in the introduction:

> *This dataset has already been used to investigate several cognitive functions. Schubert et al. (2022) showed that individual differences in attentional control can be captured with a neurocognitive psychometric approach, whereas Schubert, Löffler, Hagemann, and Sadus (2023) demonstrated that neural processing-speed measures robustly predict general cognitive ability.* (p. 2, lines 91-98)

Furthermore, we added the following paragraph to our introduction, in order to integrate our previous findings:

> *Critically, recent evidence from the dataset used in the present analyses suggests that traditional behavioral indices and the amplitudes of ERP components fail to capture executive function abilities, but merely reflect processing speed (Löffler et al., 2024, 2025). However, the failure of established indicators to isolate executive functions using traditional tasks does not preclude measuring them via other indicators within those tasks. For example, Schubert et al. (2025) found in the same dataset that mid-frontal global theta connectivity is a stable, trait-like neural signature of cognitive control that strongly correlates with general cognitive ability. Therefore, in the present analyses, we further explore, whether oscillatory dynamics can serve as measures of individual differences in flexibility. In particular, we focus on measuring flexibility in isolation from basic cognitive processes using latent change modeling.* (p. 2, lines 98-116)

This brings us to our second point. While these markers seem of use to the field, and the task-specificity of theta versus generality of alpha/beta is an interesting standalone finding, their interpretability would benefit greatly from a clearer relationship to behavior. The current paper now only briefly mentions the standard switch costs in reaction time and accuracy, but does not explore (1) underlying factor structures based on those, (2) relations between the oscillatory signatures and these behavioral markers, or (3) relation with previous DDM markers the authors developed and proposed in a previous publication on this dataset. Such behavioral analyses would be of relevance to several of the questions the authors, themselves, raise in the discussion now (e.g., the relation with transient versus

sustained changes in behavior, the relation with switching versus working memory, the uniqueness to flexibility tasks?).

Given the number of prior papers on this dataset, we would strongly encourage the authors to integrate these behavioral dimensions here rather than postponing them to another manuscript. At this stage of the research program, additional fragmentation of analyses risks obscuring the broader picture, and incorporating these behavioral relations here would substantially improve coherence.

Our decision to not include behavioral measures in the analysis was based on the fact that, in previous analyses, we had demonstrated problems with behavioral measures for both simple reaction time data and model-based drift parameters (Löffler et al., 2024). These measures are incapable of isolating executive functions from mere processing speed. However, in the present paper, we argue that the latent change model is a different and promising way of isolating pure flexibility processes. In addition, we miss the opportunity of demonstrating whether this approach is capable of capturing cognitive flexibility with behavioral measures. At the same time, we believe it is important to note that the validity of neural measures is not contingent on the relationship between neurophysiological and behavioral measures. In our manuscript we argue that neural measures have the potential to identify mechanisms that are not discernible by behavioral responses (p. 2, lines 53-56). Nevertheless, we agree that including behavioral measures and relating them to the neural markers will be insightful. We report the results of these additional analyses briefly in the main manuscript:

> *Investigating behavioral measures in conjunction with neural measures can improve our understanding of a construct. Accordingly, we tested whether cognitive flexibility can also be isolated using the drift rate v. This process-based behavioral measure is derived from the drift-diffusion model (DDM; Ratcliff, 1978) and reflects the speed of information uptake. A more detailed rationale behind these analyses and more details on the DDM can be found in the Supplement. The latent change measurement model indicated that cognitive flexibility could indeed be captured with the drift rate v, $\chi^2(12) = 12.88$, $p = .378$, CFI $\geq .99$, RMSEA $= .02$, 90% CI [.00, .09]; $\sigma^2_{unstandardized} = .11$, 95% CI [.05, .17], $p < .001$.*
>
> *Consequently, we integrated the drift-based measurement model of flexibility with the two ERSP-based measurement models of flexibility that use alpha/beta power to isolate flexibility processes. The integrated model fitted the data well, $\chi^2(132) = 201.82$, $p < .001$, CFI $= .98$, RMSEA $= .06$, 90% CI [.04, .08]. The latent correlations revealed that flexibility measured via drift rate did not share a substantial amount of variance with flexibility measured with cue-related ($r = -.14$, 95% CI [-.40, .13], $p = .301$) nor target-related alpha/beta power ($r = -.11$, 95% CI [-.37, .16], $p = .415$). In contrast, the repeat factor derived from drift rates correlated both with cue-related ($r = -.22$, 95% CI [-.38, -.06], $p = .011$) and target-related ($r = -.36$, 95% CI [-.51, -.21], $p < .001$) repeat factors, supporting the notion that oscillatory activity and cognitive measures of information processing are related, just not when reflecting flexibility specifically.*

> *As in our main analyses we found that neuronally based flexibility and repeat factors were not substantially correlated. In contrast, for behavioral flexibility we found that participants with greater drift rate in the repeat condition showed smaller switch costs (r = -.31, 95% CI [-.55, -.07], p = .015). The complete description of these analyses, including full statistics for all latent correlations, is provided in the Supplement.* (p. 16, lines 1062-1403)

Furthermore, the most important implications of these analyses are addressed in the Discussion:

> *We further showed that cognitive flexibility can be captured via process-based behavioral indicators. Yet, these and ERSP-derived neural markers reflect distinct facets of cognitive flexibility. One interpretation is that alpha/beta suppression captures preparatory attentional reconfiguration as an upstream process that enables but does not fully determine behavioral performance. The drift rate, by contrast, reflects the cumulative efficiency of evidence accumulation during target classification, a downstream process shaped by additional factors such as stimulus–response retrieval and response selection that are not uniquely engaged by switching. Alternatively, the dissociation may reflect differences in measurement granularity: oscillatory dynamics index moment-to-moment neural states with high temporal precision, whereas drift rates aggregate over the entire decision process, potentially diluting flexibility-specific variance. A similar pattern has been observed for theta connectivity in this dataset, which correlated with general cognitive abilities but not with behavioral switch costs (Schubert et al., 2025), reinforcing the broader conclusion that neural signatures of cognitive control are not merely neural translations of behavioral effects. These findings caution against treating neural and behavioral flexibility as interchangeable operationalizations of the same construct. Instead, they point toward a multicomponent architecture in which cognitive flexibility emerges from dissociable processes—some reflected in oscillatory dynamics, others in behavioral output—that may jointly but not redundantly contribute to adaptive behavior.* (p. 19, lines 1350-1380)

We also integrate these findings in the abstract:

> Notably, flexibility-related alpha/beta dynamics did not correlate with measures of intelligence or working memory capacity *nor behaviorally measured flexibility*, underscoring *that alpha/beta suppression captures a neurally coherent but behaviorally distinct dimension of cognitive flexibility*. These findings provide evidence that alpha/beta suppression constitutes a reliable, *cross-task* generalizable neural signature of *attentional reconfiguration during task switching, while highlighting the multicomponent nature of* individual differences in cognitive flexibility.

We report and discuss these additional analyses with all details in the Supplement, such that interested readers can access the information but generally the already complex set of analyses are not further expanded.

In Löffler et al. (2024) we have argued that using the drift rate v is preferable to using reaction time or accuracy data because it combines both. Therefore, it can better account for speed-accuracy trade-offs than reaction time or accuracy on their own. Furthermore, the drift diffusion model distinguishes different cognitive processes that contribute to reaction time but are not all relevant (e.g., non-decision time). Therefore, we estimated a latent change measurement model for the drift rate v to isolate cognitive flexibility. The model converged and fit the data well. Flexibility could be captured by this model, as the latent change residual was significant. Therefore, we combined the three different latent change models, modeling flexibility via cue-related alpha/beta activity, target-related alpha/beta-activity, and drift rate. We allowed correlations between the flexibility factors of all three latent change models as well as correlations between the low-demand repeat conditions. Here we found that the neural flexibility markers are highly correlated, while they do not share a substantial amount of variance with behavioral flexibility measures.

Minor:

We would consider dropping the "of individual differences" in the title. The title right now is a bit on the long side, and while this study sure has important implications for the study of individual differences, we found its implications to extend beyond that.

Indeed, the title is rather long. Therefore, we removed "of individual differences" from the title.

The results section was now written relatively descriptively. We thought it would be very helpful if the authors could provide a little bit more of a narrative in the results section, interim conclusions, or small summaries.

We followed this advice by adding descriptions and conclusions throughout the results section. All changes are highlighted in the manuscript (lines 775-776, 787-789, 796, 806, 818-824, 827-829, 831-833, 875-877, 879-882, 898-902, 915-916, 953-954, 971-981, 984-986, 988, 1006-1009, 1047-1049)

Line 621, please provide references for the sentence that starts with "In line with previous research, the reliabilities of the ...".

We included references to this statement: We now cite Draheim, Tsukahara, Martin, Mashburn, and Engle (2021), generally discussing the problems of manifest difference scores and (von Bastian et al., 2020) reporting the reliabilities of manifest difference scores explicitly for behavioral data of shifting tasks (page 9, lines 680-682).

Other relevant papers may be:

Angelidis, A., van der Does, W., Schakel, L., & Putman, P. (2016). Frontal EEG theta/beta ratio as an electrophysiological marker for attentional control and its test-retest reliability. Biological psychology, 121, 49-52.

We thank the reviewer for pointing out Angelidis et al. (2016). Although that work offers valuable insights into resting-state theta/beta ratio as a predictive electrophysiological marker of attentional control, it does not directly address the specific questions of our investigation, which focuses on event-related oscillatory signatures of individual differences in cognitive flexibility across three cued task-switching paradigms. Consequently, we have opted not to include it in our manuscript.

McKewen, M., Cooper, P. S., Skippen, P., Wong, A. S., Michie, P. T., & Karayanidis, F. (2021). Dissociable theta networks underlie the switch and mixing costs during task switching. Human Brain Mapping, 42(14), 4643-4657.

The work of McKewen et al. (2021) focuses on theta inter-site phase clustering and identifies differential, condition-specific theta effects upon cue (switch effects), but no differential effects upon target. We included this study in our discussion:

> *The lack of cross-task generalizability may also suggest that other parameters than power–for example theta connectivity–underlie cognitive flexibility. This is supported by McKewen et al. (2021), who showed that trial-specific theta connectivity patterns within the fronto-parietal network prepare the cognitive system for the impending need to switch or repeat. Moreover, our own work on this dataset confirms that fronto-parietal theta connectivity robustly indexes flexibility across tasks (Schubert et al., 2025).* (p. 18, lines 1221-1230)

Reviewer #3 (Remarks to the Author):

I commend the authors for addressing an important issue in the field and adding to pre-existing literature in this vein of work. I have several points of improvement that the authors should address before this piece is ready for publication.

We thank the reviewer for taking the time to read our manuscript and provide valuable suggestions for improvement.

Introduction

- Strengthen the justification for why flexibility should or should not relate to general cognitive abilities by explicitly engaging models such as Process Overlap Theory (Kovacs & Conway, 2016), hierarchical control accounts, and resource-based frameworks.

  We agree that a deeper theoretical grounding would strengthen our argument. We have now explicitly engaged the process overlap theory and resource-based frameworks and thereby expand on our argument that, even though flexibility is a clearly defined, independent construct, it should be related to general cognitive abilities.

  > *For example, the process overlap theory (Kovacs & Conway, 2016) posits that domain-general cognitive processes, such as core executive functions, act as bottlenecks that cause the positive manifold. In contrast, resource-based frameworks (cf. Schubert, Löffler, Sadus, et al., 2023) conceptualize*

> *core executive functions as limited resources, predicting that individuals with greater resources will perform better. Taken together, many influential theoretical frameworks suggest that flexibility plays an important role for general cognitive abilities.* (p. 2, lines 41-51)

- Incorporate alternative interpretations of theta and alpha/beta oscillatory functions and clarify why the chosen interpretations best fit the present findings.

  We concur that alternative interpretations of theta and alpha/beta oscillatory functions are an important aspect that should be discussed. We incorporated such alternative interpretations in previous versions of the Introduction, but we had the feeling that this disrupted the flow of reading. We therefore opted for including this topic in the Discussion:

  > *We have focused our interpretation of theta and alpha/beta oscillations on cognitive control and attentional processes, respectively. While there is substantial evidence to support this interpretation, there are also alternative interpretations (Keitel et al., 2025). For example, the widespread alpha/beta effect, may reflect brain-wide reconfiguration processes of neuronal networks, in order to meet the task-demands (Gratton, 2018). Alpha suppression, in particular, is indicative of an activation of the respective region (Haegens et al., 2011). However, alpha oscillations are not merely a marker of cortical idling. Instead, they actively shape cognition by modulating the excitability of specific neuronal populations (Lange et al., 2013), thereby selectively activating or inhibiting particular cortical regions (Sadaghiani & Kleinschmidt, 2016). While alternative interpretations of oscillatory dynamics exist, they converge on the necessity of neural reconfiguration to meet task demands. In the case of cued task-switching, this implies the reallocation of attentional weights and the maintenance of task-relevant neural networks from cue onset through target processing (Kiesel et al., 2010).* (page 18-19, lines 1266-1288)

  We would like to mention that this paragraph was not only added to meet this comment, but also to meet a distinct, but related comment of Reviewer #1.

- Clarify the theoretical status of alpha/beta suppression— does it index attentional reallocation, cognitive control, or more domain-general feedback processes? Explain how these possibilities relate to flexibility specifically.

We interpret alpha/beta suppression as domain-general attentional reallocation rather than pure cognitive control or generic feedback processes. This specific interpretation is grounded in the temporal and condition-specific pattern of the alpha/beta suppression and its generalizability across tasks. The alpha/beta suppression persisted from cue through target processing across all three switching tasks. This pattern aligns with models of top-down attentional control where alpha/beta oscillations modulate the excitability of neuronal

populations to selectively activate task-relevant networks while suppressing competing representations. We now discuss this aspect in more detail on (page 18-19, lines 1266-1288). The alpha/beta effect relates to flexibility because there is stronger suppression in switch trials than in repeat trials. This could reflect the higher processing demands of switch trials, which require more extensive deactivation of previous networks and activation of rule-set-specific networks. We discuss this aspect on (p. 20, lines 1416-1424).

We do not interpret alpha/beta suppression as specific marker for cognitive control. There is substantial evidence that the need for cognitive control is signaled by frontal theta activity (Cavanagh & Frank, 2014; Cavanagh & Shackman, 2015) and distributed via the fronto-parietal network by inter-site phase clustering (e.g. McKewen et al., 2021). The alpha/beta suppression we see, seems to originate in the parietal cortex, which has rather been associated with receiving control commands and adjust attentional weights accordingly (McKewen et al., 2021; Peylo, Hilla, & Sauseng, 2021; Behrmann, Geng, & Shomstein, 2004) (cf. p. 18, lines 1239-1242).

Furthermore, we do not think that alpha/beta suppression indexes domain-general feedback processes. The suppression appears already during the cue period and persists through target processing, indicating a prospective allocation of resources that prepares the system for an upcoming rule change rather than a reactive feedback process.

We hope that the paragraph about alternative interpretations of alpha/beta suppression (page 18-19, lines 1266-1288) together with section "Disentangling cue- and target-related processes" (p. 20, lines 1394-1444) addresses these aspects.

Methodology

- Address the impact of relatively high trial/epoch rejection rates on SNR and discuss whether this could bias individual differences estimates.

  We thank the reviewer for highlighting the potential impact of trial rejection rates on SNR and on individual-differences estimates. While we appreciate the concern, we respectfully disagree that the rejection rates in our dataset are relatively high. Rejection rates for trials were in the expected range for errors (cf. Meiran, 2000), reaction time outliers (cf. Berger & Kiefer, 2021), and EEG artifacts (cf. Delorme, Sejnowski, & Makeig, 2007). Conducting a study with electrophysiological recording, which will lead to trial rejection due to EEG artifacts, we a priori chose an accordingly number of trials: 384 trials for the parity/magnitude and global/local task, and 256 trials for the number/letter task. In the revised manuscript we added the information that these rejection rates are consistent with typical values reported in the literature (p. 8, lines 553-556). We furthermore added the above citations, to give the readers further orientation.

  > *Rejection rates for trials were in the expected range for errors (Meiran, 2000), reaction time outliers (Berger & Kiefer, 2021), and EEG artifacts (Delorme et al., 2007).* (p. 8, lines 553-556)

- Provide justification for relying on latent-change modeling when manifest reliability is near-zero for some indicators; clarify whether the latent construct contains sufficient signal to support trait-level interpretation.

There might be a misunderstanding, which we would like to clarify. Reliabilities of manifest difference scores were unacceptable, which is why we refrained from using those as manifest indicators. We report this on p. 9 lines 680-689. The reliabilities for absolute power values in the switch and repeat conditions however were excellent for alpha/beta power ($\geq$ .95) and at least acceptable, but mostly good for theta power (.65 – .86).

- Discuss whether the lack of generalizability for the theta and beta clusters truly reflects task specificity or could reflect lower cross-task variance or insufficient statistical power?

In case of the target-related theta and beta clusters, we judge them as not generalizable because first, these clusters became only significant in two of the three tasks and second, because of their temporal structure. These clusters either did not overlap across tasks in case of theta or emerged clearly after the behavioral response had been given, and therefore after when we can expect cognitive flexibility processes. This is why we did not submit these to structural equation modeling. We discuss this on (p. 14, lines 934-970).

In case of the cue-related theta cluster, we argue that it is not generalizable because we cannot extract a significant flexibility factor. That is, there must be lower cross-task variance specific to flexibility processes, resulting in a non-significant flexibility factor. Since we are interpreting null findings here, we have added a model comparison to the revised version in which the original latent change model is compared with a constrained model:

> The latent change model with cue-related theta ERSP explained the data well, $\chi^2(12) = 19.16$, $p = .085$, CFI = .98, RMSEA = .06, 90% CI [.00, .12], AIC = 1958.52 (Figure 2). However, flexibility could not be captured by this model, as the variance of the latent change factor was not significant ($\sigma^2_{\text{unstandardized}} = .04$, 95% CI [-.09, .17], $p = .517$). *In order to interpret these null findings, we compared this model with a more constrained latent change model, where we fixed the variance of the flexibility factor and its correlation with the repeat factor to zero. If there is indeed no shared flexibility-specific variance, the constrained latent change model should fit the data as good or even better as the initial model. Indeed, model fit remained good, $\chi^2(14) = 20.03$, p = .129, CFI = .99, RMSEA = .05, 90% CI [.00, .11], AIC = 1955.39. According to the Akaike weights (initial model: .17; constrained model: .83), the constrained model is 4.80 times more likely than the unconstrained model.* (p. 11, lines 854-866)

All our measurement models suffer from insufficient statistical power, which makes it difficult to fully judge them on their own. On the contrary, our full integrated model assessing the relationship of alpha-based cue- and target-related flexibility with general cognitive abilities and WMC has sufficient power. Because the problem of insufficient power applies equally to all our measurement models of flexibility, insufficient power cannot inevitably lead to non-generalizable results.

Statistical Modeling and Interpretation

- Clarify the interpretational implications of the target-related alpha/beta suppression beginning prior to target onset—does this reflect anticipatory processing rather than target-driven flexibility?

  We appreciate this relevant concern. We indeed think that the beginning of the target-related alpha/beta suppression prior to target onset reflects anticipatory attentional processes. Together with the latent near one-correlation of cue-related and target-related cognitive flexibility and the spectral and spatial overlap of the effects, we believe that indeed, they reflect a single, sustained attentional process rather than separate mechanisms. We discuss the theoretical implications of this effect pattern on p. 20, lines 1394-1444 in the Discussion. In essence, the near-perfect correlation between cue- and target-related flexibility suggests these are not distinct processes but a single, sustained attentional mechanism. Three converging lines of evidence support this interpretation: (1) both effects share the same frequency range and scalp topography; (2) they overlap temporally; (3) the pattern is consistent with two-stage models of task switching, where cue-initiated attentional reconfiguration must be maintained through target processing because network reconfiguration cannot be completed before the target appear. In other words, the cue signals the need for greater resource allocation on switch trials (reflected in stronger alpha/beta suppression), and this elevated attentional state is sustained continuously until and through target processing, rather than being re-engaged as a separate target-driven process.

- More clearly articulate limitations of the mass-univariate approach, particularly that cluster extent cannot be precisely localized in space or frequency.

  We fully agree with the reviewer that this is an easily overseen and highly important restriction of the mass-univariate approach. This is why we emphasize this restriction in the Methods section (p. 9, lines 665-668). However, the brief mentioning in the Methods section might easily be overseen by readers, which is why we added this aspect to the Limitations section:

  > *Furthermore, it is essential to keep in mind that cluster-based permutation tests are based on probabilistic test statistics (here t-tests). Consequently, the precise localization of temporal, spectral, and spatial cluster extent is not feasible (Sassenhagen & Draschkow, 2019).* (page 21, lines 1489-1494)

Conclusions and Claims

- Reframe claims that flexibility is 'distinct' from intelligence or WMC as preliminary given measurement constraints, range restriction, and modest reliability of some indicators. This conclusion should be qualified at the minimum.

  We reframed the claim of flexibility being distinct from intelligence and WMC in the revised manuscript:

  > Taken together, we find *preliminary evidence* that alpha/beta suppression is attributable to a general flexibility factor, *that appears to be independent*

> from other performance measures, such as intelligence and WMC. (page 20, lines 1381 and 1383)

Furthermore, we modified our conclusion that the detected non-significant correlation of flexibility with general cognitive abilities and WMC is in accordance with previous findings:

> However, neither flexibility factor correlated significantly with intelligence or WMC*. This is consistent with* previous findings of *a* dissociation between flexibility and general cognitive ability (Kopp, Maldonado, Scheffels, Hendel, & Lange, 2019; Santarnecchi et al., 2021). Nevertheless, empirical findings on the relationship between flexibility, intelligence, and WMC are inconsistent and may furthermore depend on the specific neuronal or behavioral indicators used (Schmitz & Krämer, 2023). *Importantly, we have not explicitly tested the null hypothesis of an independence between the latent constructs. The confidence intervals of the estimated correlations do not exclude the existence of small or even medium effects (cf. Table A3).* (page 19, lines 1328-1341)

- Temper language suggesting definitive neural markers; emphasize instead that alpha/beta suppression is a promising and replicable candidate but requires further validation.

  We think this is a perfectly valid suggestion. We now emphasize that alpha/beta suppression is a promising neural signature of cognitive flexibility, but not a definitive marker. We explicitly call for further validation:

  > We identified alpha/beta suppression as a *promising candidate for a* neural marker of domain-general flexibility *awaiting further validation*, while demonstrating that theta effects reflect task-specific control processes. (p. 21, lines 1523-1525)

  > By *identifying* alpha/beta suppression as a *potential* neural signature of domain-general flexibility, this work provides a neurobiological foundation for cognitive flexibility as a distinct construct. (p. 21, line 1545)

- Discuss future work explicitly—including improving neural reliability, testing broader cognitive batteries, and conducting replication studies—to position conclusions within a developmental trajectory rather than a definitive endpoint. Just suggestions.

  We consent with the reviewer that providing a clearer description of potential avenues for future research would be very useful. In the revised manuscript we give a more explicit outlook:

  > *Future studies should extend these findings by using broader cognitive batteries to determine if alpha/beta suppression, as a domain-general attentional process, generalizes beyond cued task-switching. Additionally, replication*

studies should address the limitations of these analyses to determine if alpha/beta dynamics can serve as a robust neural marker of cognitive flexibility across populations and paradigms. (p. 22, lines 1561-1568)

References

Behrmann, M., Geng, J. J., & Shomstein, S. (2004, April). Parietal cortex and attention. *Current Opinion in Neurobiology*, *14*(2), 212–217. doi: 10.1016/j.conb.2004.03.012

Berger, A., & Kiefer, M. (2021, June). Comparison of Different Response Time Outlier Exclusion Methods: A Simulation Study. *Frontiers in Psychology*, *12*, 675558. doi: 10.3389/fpsyg.2021.675558

Buzsáki, G. (2006). *Rhythms of the Brain*. Oxford University Press. doi: 10.1093/acprof:oso/9780195301069.001.0001

Cavanagh, J. F., & Frank, M. J. (2014, August). Frontal theta as a mechanism for cognitive control. *Trends in Cognitive Sciences*, *18*(8), 414–421. doi: 10.1016/j.tics.2014.04.012

Cavanagh, J. F., & Shackman, A. J. (2015, February). Frontal midline theta reflects anxiety and cognitive control: Meta-analytic evidence. *Journal of Physiology-Paris*, *109*(1), 3–15. doi: 10.1016/j.jphysparis.2014.04.003

Delorme, A., Sejnowski, T., & Makeig, S. (2007, February). Enhanced detection of artifacts in EEG data using higher-order statistics and independent component analysis. *NeuroImage*, *34*(4), 1443–1449. doi: 10.1016/j.neuroimage.2006.11.004

Draheim, C., Tsukahara, J. S., Martin, J. D., Mashburn, C. A., & Engle, R. W. (2021, February). A toolbox approach to improving the measurement of attention control. *Journal of Experimental Psychology: General*, *150*(2), 242–275. doi: 10.1037/xge0000783

Gratton, G. (2018, March). Brain reflections: A circuit-based framework for understanding information processing and cognitive control. *Psychophysiology*, *55*(3), e13038. doi: 10.1111/psyp.13038

Haegens, S., Cousijn, H., Wallis, G., Harrison, P. J., & Nobre, A. C. (2014, May). Inter- and intra-individual variability in alpha peak frequency. *NeuroImage*, *92*, 46–55. doi: 10.1016/j.neuroimage.2014.01.049

Haegens, S., Nácher, V., Luna, R., Romo, R., & Jensen, O. (2011, November). α-Oscillations in the monkey sensorimotor network influence discrimination performance by rhythmical inhibition of neuronal spiking. *Proceedings of the National Academy of Sciences*, *108*(48), 19377–19382. doi: 10.1073/pnas.1117190108

Harris, A. M., Dux, P. E., Jones, C. N., & Mattingley, J. B. (2017, May). Distinct roles of theta and alpha oscillations in the involuntary capture of goal-directed attention. *NeuroImage*, *152*, 171–183. doi: 10.1016/j.neuroimage.2017.03.008

Karr, J. E., Areshenkoff, C. N., Rast, P., Hofer, S. M., Iverson, G. L., & Garcia-Barrera, M. A. (2018). The unity and diversity of executive functions: A systematic review and re-analysis of latent variable studies. *Psychological Bulletin*, *144*(11), 1147–1185. doi: 10.1037/bul0000160

Keitel, A., Keitel, C., Alavash, M., Bakardjian, K., Benwell, C. S. Y., Bouton, S., … Kotz, S. A. (2025, July). *Brain rhythms in cognition – controversies and future directions* (No. arXiv:2507.15639). arXiv. doi: 10.48550/arXiv.2507.15639

Kiesel, A., Steinhauser, M., Wendt, M., Falkenstein, M., Jost, K., Philipp, A. M., & Koch, I. (2010). Control and interference in task switching—A review. *Psychological Bulletin*, *136*(5), 849–874. doi: 10.1037/a0019842

Kopp, B., Maldonado, N., Scheffels, J. F., Hendel, M., & Lange, F. (2019, December). A Meta-Analysis of Relationships between Measures of Wisconsin Card Sorting and Intelligence. *Brain Sciences*, *9*(12), 349. doi: 10.3390/brainsci9120349

Kovacs, K., & Conway, A. R. A. (2016, July). Process Overlap Theory: A Unified Account of the General Factor of Intelligence. *Psychological Inquiry*, *27*(3), 151–177. doi: 10.1080/1047840X.2016.1153946

Lange, J., Oostenveld, R., & Fries, P. (2013, February). Reduced Occipital Alpha Power Indexes Enhanced Excitability Rather than Improved Visual Perception. *Journal of Neuroscience*, *33*(7), 3212–3220. doi: 10.1523/JNEUROSCI.3755-12.2013

Löffler, C., Frischkorn, G. T., Hagemann, D., Sadus, K., & Schubert, A.-L. (2024, June). The common factor of executive functions measures nothing but speed of information uptake. *Psychological Research*, *88*(4), 1092–1114. doi: 10.1007/s00426-023-01924-7

Löffler, C., Sadus, K., Frischkorn, G. T., Hagemann, D., & Schubert, A.-L. (2025). The factor structure of executive functions measured with electrophysiological correlates: An event-related potential analysis. *Journal of Experimental Psychology: Learning, Memory, and Cognition*. doi: 10.1037/xlm0001549

McKewen, M., Cooper, P. S., Skippen, P., Wong, A. S. W., Michie, P. T., & Karayanidis, F. (2021). Dissociable theta networks underlie the switch and mixing costs during task switching. *Human Brain Mapping*, *42*(14), 4643–4657. doi: 10.1002/hbm.25573

Meiran, N. (2000, August). Modeling cognitive control in task-switching. *Psychological Research*, *63*(3), 234–249. doi: 10.1007/s004269900004

Miles, S., Howlett, C. A., Berryman, C., Nedeljkovic, M., Moseley, G. L., & Phillipou, A. (2021, October). Considerations for using the Wisconsin Card Sorting Test to assess cognitive flexibility. *Behavior Research Methods*, *53*(5), 2083–2091. doi: 10.3758/s13428-021-01551-3

Miyake, A., Friedman, N. P., Emerson, M. J., Witzki, A. H., Howerter, A., & Wager, T. D. (2000, August). The Unity and Diversity of Executive Functions and Their Contributions to Complex "Frontal Lobe" Tasks: A Latent Variable Analysis. *Cognitive Psychology*, *41*(1), 49–100. doi: 10.1006/cogp.1999.0734

Peylo, C., Hilla, Y., & Sauseng, P. (2021, September). Cause or consequence? Alpha oscillations in visuospatial attention. *Trends in Neurosciences*, *44*(9), 705–713. doi: 10.1016/j.tins.2021.05.004

Ratcliff, R. (1978). A theory of memory retrieval. *Psychological Review*, *85*(2), 59–108. doi: 10.1037/0033-295X.85.2.59

Sadaghiani, S., & Kleinschmidt, A. (2016, November). Brain Networks and α-Oscillations: Structural and Functional Foundations of Cognitive Control. *Trends in Cognitive Sciences*, *20*(11), 805–817. doi: 10.1016/j.tics.2016.09.004

Santarnecchi, E., Momi, D., Mencarelli, L., Plessow, F., Saxena, S., Rossi, S., … Pascual-Leone, A. (2021, April). Overlapping and dissociable brain activations for fluid intelligence and executive functions. *Cognitive, Affective, & Behavioral Neuroscience*, *21*(2), 327–346. doi: 10.3758/s13415-021-00870-4

Sassenhagen, J., & Draschkow, D. (2019). Cluster-based permutation tests of MEG/EEG

data do not establish significance of effect latency or location. *Psychophysiology*, *56*(6), e13335. doi: 10.1111/psyp.13335

Schmitz, F., & Krämer, R. J. (2023, April). Task Switching: On the Relation of Cognitive Flexibility with Cognitive Capacity. *Journal of Intelligence*, *11*(4), 68. doi: 10.3390/jintelligence11040068

Schubert, A.-L., Löffler, C., & Hagemann, D. (2022, September). A neurocognitive psychometrics account of individual differences in attentional control. *Journal of Experimental Psychology: General*, *151*(9), 2060–2082. doi: 10.1037/xge0001184

Schubert, A.-L., Löffler, C., Hagemann, D., & Sadus, K. (2023). How robust is the relationship between neural processing speed and cognitive abilities? *Psychophysiology*, *60*(2), e14165. doi: 10.1111/psyp.14165

Schubert, A.-L., Löffler, C., Jungeblut, H. M., & Hülsemann, M. J. (2025). Trait characteristics of midfrontal theta connectivity as a neurocognitive measure of cognitive control and its relation to general cognitive abilities. *Journal of Experimental Psychology: General*. doi: 10.1037/xge0001780

Schubert, A.-L., Löffler, C., Sadus, K., Göttmann, J., Hein, J., Schröer, P., ... Hagemann, D. (2023, July). Working memory load affects intelligence test performance by reducing the strength of relational item bindings and impairing the filtering of irrelevant information. *Cognition*, *236*, 105438. doi: 10.1016/j.cognition.2023.105438

Vezoli, J., Vinck, M., Bosman, C. A., Bastos, A. M., Lewis, C. M., Kennedy, H., & Fries, P. (2021, December). Brain rhythms define distinct interaction networks with differential dependence on anatomy. *Neuron*, *109*(23), 3862-3878.e5. doi: 10.1016/j.neuron.2021.09.052

Vissers, M. E., Ridderinkhof, K. R., Cohen, M. X., & Slagter, H. A. (2018, April). Oscillatory Mechanisms of Response Conflict Elicited by Color and Motion Direction: An Individual Differences Approach. *Journal of Cognitive Neuroscience*, *30*(4), 468–481. doi: 10.1162/jocn_a_01222

von Bastian, C. C., Blais, C., Brewer, G., Gyurkovics, M., Hedge, C., Kałamała, P., ... Wiemers, E. (2020, July). *Advancing the understanding of individual differences in attentional control: Theoretical, methodological, and analytical considerations.* OSF. doi: 10.31234/osf.io/x3b9k

Zhang, Y., Deng, F., Liao, J., Verguts, T., & Chen, Q. (2026, January). Medial Frontal Theta Reduction Impairs Rule Switching via Prediction Error. *Journal of Neuroscience*, *46*(1). doi: 10.1523/JNEUROSCI.1236-25.2025

Response Letter

Reviewer #1 (Remarks to the Author):

The authors have been responsive in this revision: both to my previous concerns as well as to issues raised by other reviewers. Yet, notably, most of my concerns were not procedural or identifying issues with specific choices: they were examples of cases where this data-driven analysis and post-hoc interpretation failed to lead to novel theoretical conclusions. This manuscript remains a technical tour-de-force, but I think it will have only a minor impact on the field due to raising more questions than it answers.

We would like to thank the reviewer for their constructive feedback on our manuscript, and for acknowledging the analytical strengths of the paper.

Reviewer #2 (Remarks to the Author):

We would like to thank the authors for responding to our comments, providing extra analyses, and revising their manuscript. We believe the manuscript has certainly improved. However, one of the new additions was to show a relation with behavior for which the authors now provide analyses that link drift diffusion measures to their oscillatory measures of EEG activity. We thought those new analyses were interesting, but believe some important analysis details are missing and the results could be better related to broader theories of task switching. As this already concerns a revised manuscript, we tried to keep our comments targeted:

We sincerely thank the reviewers for their insightful suggestion to conduct additional analyses. These not only strengthened the robustness of our findings but also led to intriguing new results that enriched our interpretation. We are especially grateful that the reviewers built upon these findings with further thoughtful suggestions, which we have now carefully addressed in this revision.

First, we were wondering how different parameters were estimated. Specifically, for which experimental conditions were the individual parameters estimated separately versus shared? The paper refers to Löffler et al. for further details, but we could not immediately find those details there either, and believe these should be repeated in the main manuscript. From the results section, we inferred that the authors must have estimated separate drift rates for task repetitions and task switches? However, did they also do this for non-decision times? The latter would be important as previous studies using DDM in task switching usually rely on the distinction between those two, with differences in drift rates been argued to reflect carry-over effects of the previous task and non-decision time a reconfiguration process (e.g., see Hartanto & Yang, 2016; Imburgio & Orr, 2021; Schmitz & Voss, 2012; 2014). Therefore, we would be interested in seeing the results for relations with switch- versus repetition-related non-decision times, too.

We thank the reviewer for pointing out that information on parameter estimation was missing. We have now added this information.

> *The parameters t_0 and v were estimated separately for the two experimental conditions switch and repeat. All other parameters were estimated jointly across*

conditions. (page 11, lines 806-809)

As we had not estimated t_0 separately for the switch and repeat condition in our prior analyses, we re-ran these analyses and therefore now have slightly different numerical results. These analyses were quite insightful. We found that alpha/beta-derived cue-related flexibility is associated with non-decision time at an inter-individual level. The stronger the alpha/beta-decrease in response to the cue, the shorter the non-decision interval as estimated with the drift diffusion model. Therefore this finding could indicate that the effectiveness of task preparation in switch trials is contingent upon the magnitude of alpha/beta suppression elicited by the cue. The correlation then weakens and becomes non-significant in relation to target-related alpha/beta suppression. This pattern was to be expected because of the rather long cue-target interval implemented in this study (800-1200 ms). Participants were likely able to complete anticipatory task preparations before target onset (Schmitz & Voss, 2012). Notably, flexibility-related alpha/beta suppression was exclusively associated with non-decision time, but not with drift rate.

We decided to migrate all diffusion model related results from the Supplement into the main manuscript, since all the key results are reported and discussed there anyway. Keeping them in the Supplement would have resulted in many redundancies. The migrated parts are highlighted in gray (lines 214-218, 783-816, 1119-1123, 1140-1145, 1165-1169, 1502-1518). All changes in comparison to the previous manuscript version are marked. We summarize the findings in the abstract and further motivate the DDM-analyses in the introduction:

> *However, stronger cue-elicited alpha/beta suppression correlated with reduced non-decision-time switch costs, indicating more effective task preparation, underscoring that alpha/beta suppression captures a neurally coherent but behaviorally distinct dimension of cognitive flexibility.* (page 1, abstract)

> *Furthermore, for neural measures to serve as meaningful individual difference variables, they must demonstrate convergent validity across different flexibility tasks. Moreover, they should demonstrate convergent validity across different flexibility measures, such as process-based behavioral measures.*(page 3, lines 153-156)

We updated the results section and discuss the findings:

> *We further showed that cognitive flexibility can be captured via process-based behavioral indicators. Yet, these and ERSP-derived neural markers reflect distinct facets of cognitive flexibility. One interpretation is that alpha/beta suppression captures preparatory attentional reconfiguration which enables but does not fully determine behavioral performance. Crucially, at the inter-individual level, the magnitude of alpha/beta suppression in response to the cue was linked to switch costs in non-decision time: the stronger the suppression, the less strong the prolongation of the non-decision interval in switch vs. repeat trials. This suggests that the effectiveness of task preparation in switch trials may depend on the degree of alpha/beta suppression elicited by the cue (Schmitz & Voss, 2012; Karayanidis et al., 2009). The drift rate, by contrast, reflects the cumulative efficiency of evidence accumulation during target classification, a process*

> *shaped by additional factors such as stimulus–response retrieval and response selection that are not uniquely engaged by switching. This dissociation maps onto the diffusion-model decomposition of task-switching costs, in which non-decision time has been tied to anticipatory task-set reconfiguration and drift rate to carry-over from the previously active task set (Schmitz & Voss, 2012, 2014). That a preparatory neural marker, cue-locked alpha/beta suppression, tracked the non-decision-time component of flexibility but not the drift-rate component is consistent with this parameter-specific division of labor. We note, however, that this parameter mapping was established through experimental manipulation at the group level, whereas the present association concerns individual differences; the two levels need not converge, so our t_0-specific result should be read as convergent rather than confirmatory.* (pages 20-21, lines 1433-1442)

Second, the authors seem to emphasize that their relations with behavior are not related to task switching, and reflect distinct dimensions, because the brain-behavior correlations are driven by task repetition trials. However, classic and contemporary models of cognitive control suggest that task switching is a dynamic negotiation between task stability and flexibility (Allport et al., 1994; Gilbert & Shallice, 2002; Xu, Verguts, & Braem, 2026). In these frameworks, the drift rate on repetitions is a direct proxy for the "strength" or "inertia" of a task set. If the authors' oscillatory measure predicts drift rates on repetitions, it may be capturing the neural signature of task-set persistence, the very state that must be overcome during a switch? Therefore, the authors could consider discussing their findings in the context of models where flexibility is the emergent product of both inertia and reconfiguration?

We thank the reviewers in general for the suggestion to relate our neuronal findings with parameters from the drift diffusion model and pointing out the relevance of the drift parameter as well as the non-decision time. These analyses indeed give valuable insights. As the reviewers have outlined, our findings align with the interpretation of alpha/beta suppression as a reflection of task-set persistence. However, we would like to emphasize the necessity of caution when interpreting the correlations in the repeat conditions. As we have shown in Löffler, Frischkorn, Hagemann, Sadus, and Schubert (2024) process-based behavioral measures can be fully explained by simpler cognitive processes such as processing speed. Therefore measures should be taken to disentangle basic cognitive processes from task-switching specific effects. More importantly, future research should test alternative explanations against the proposed task inertia effect. We discuss the topic as suggested by the reviewers.

> *Task switching is theorized to involve an ongoing, adaptive interplay between maintaining task stability and allowing for flexibility (Xu, Verguts, & Braem, 2026). Drift rate has been associated with task-set inertia, i.e., the strength of the activation of the task-set in the previous trial (Allport, Styles, & Hsieh, 1994; Schmitz & Voss, 2012). Notably, this association is driven by the repeat trials. Our findings of correlations between alpha/beta suppression and drift rate in the repeat trials are consistent with this interpretation. Since alpha/beta suppression begins as soon as the cue is presented, and target-related alpha/beta suppression occurs even before the target appears, it stands to reason that alpha/beta suppres-*

sion is temporally positioned to support more effective evidence accumulation. Critically, alternative explanations to task-set inertia cannot be discounted and require explicit experimental testing (e.g., by varying the response-cue interval). Generally, interpretations of these correlations are difficult as long as no measures have been taken to account for the basic cognitive processes, that are also present in the repeat trials (Löffler et al., 2024). (page 21, lines 1480-1501)

Reviewer #3 (Remarks to the Author):

The paper is now adequate for publication. Thank you to the authors for addressing the comments provided.

We would like to thank the reviewer for taking the time to review our manuscript and for providing us with valuable feedback that has enabled us to enhance several key aspects of it.

Reviewer #4 (Remarks to the Author):

I co-reviewed this manuscript with one of the reviewers who provided the listed reports. This is part of the Communications Psychology initiative to facilitate training in peer review and to provide appropriate recognition for Early Career Researchers who co-review manuscripts.

We sincerely thank the co-reviewer for their thoughtful engagement with our manuscript. We address the remaining aspects above.

References

Allport, A., Styles, E., & Hsieh, S. (1994). Shifting Intentional Set - Exploring the Dynamic Control of Tasks. In C. Umilta & M. Moscovitch (Eds.), *Attention and Performance Xv: Conscious and Nonconscious Information Processing* (Vol. 15, pp. 421–452). Cambridge: M I T Press.

Karayanidis, F., Mansfield, E. L., Galloway, K. L., Smith, J. L., Provost, A., & Heathcote, A. (2009, June). Anticipatory reconfiguration elicited by fully and partially informative cues that validly predict a switch in task. *Cognitive, Affective, & Behavioral Neuroscience*, *9*(2), 202–215. doi: 10.3758/CABN.9.2.202

Löffler, C., Frischkorn, G. T., Hagemann, D., Sadus, K., & Schubert, A.-L. (2024, June). The common factor of executive functions measures nothing but speed of information uptake. *Psychological Research*, *88*(4), 1092–1114. doi: 10.1007/s00426-023-01924-7

Schmitz, F., & Voss, A. (2012). Decomposing task-switching costs with the diffusion model. *Journal of Experimental Psychology: Human Perception and Performance*, *38*(1), 222–250. doi: 10.1037/a0026003

Schmitz, F., & Voss, A. (2014, September). Components of task switching: A closer look at task switching and cue switching. *Acta Psychologica*, *151*, 184–196. doi: 10.1016/j.actpsy.2014.06.009

Xu, S., Verguts, T., & Braem, S. (2026, January). Cognitive flexibility versus stability via activation-based and weight-based adaptations. *Communications Psychology*. doi: 10.1038/s44271-026-00397-9

Response Letter

Reviewer #2 (Remarks to the Author):

I'd like to thank the authors for considering our comments and suggestions. I think this manuscript has much improved, and believe it is a great contribution to the literature. I have no further suggestions.

Reviewer #4 (Remarks to the Author):

I co-reviewed this manuscript with one of the reviewers who provided the listed reports. This is part of the Communications Psychology initiative to facilitate training in peer review and to provide appropriate recognition for Early Career Researchers who co-review manuscripts.

We would like to thank the reviewers for their thoughtful and encouraging feedback. We are grateful for the suggested analyses, which we believe have substantially improved the rigor of our statistical work and enhanced the interpretability of the results.
