# Supplementary material for: Task-specific theta enhancement and domain-general alpha/beta suppression as oscillatory signatures of cognitive flexibility
Source: Commun Psychol. 2026 Oct 15;4:140. doi: 10.1038/s44271-026-00521-9 (PMC13672302; doi:10.1038/s44271-026-00521-9)
Supplement: Supplementary file 2 [file 44271_2026_521_MOESM2_ESM.pdf]

# Task-specific theta enhancement and domain-general alpha/beta suppression as oscillatory signatures of cognitive flexibility

Mareike Hülsemann*, Christoph Löffler, and Anna-Lena Schubert
Department of Psychology, Johannes Gutenberg University Mainz, Mainz Germany

**Supplementary Figures and Tables**

**Figure S1**

*Equidistant electrode layout used in the current study.*

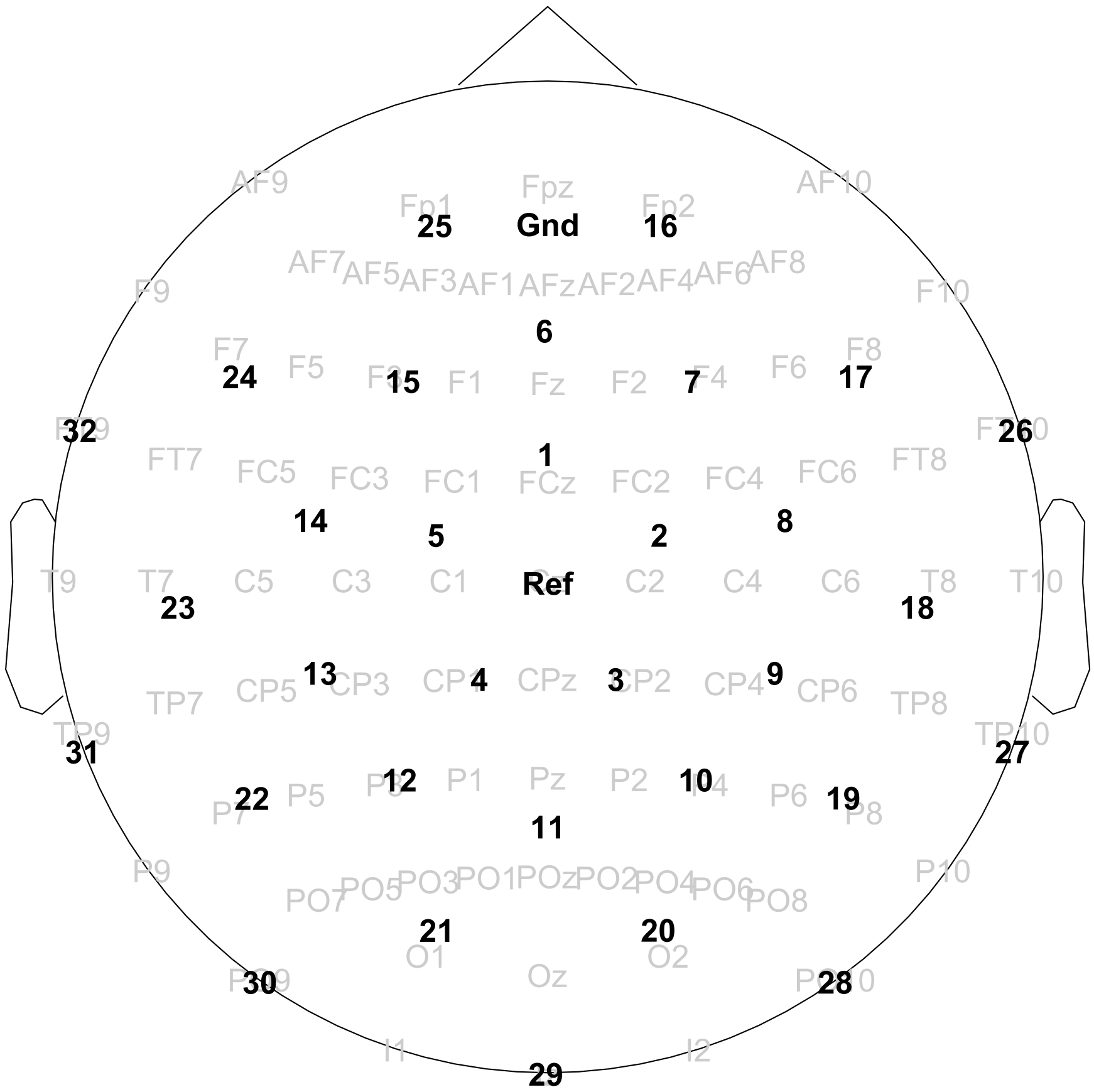


*Note.* The 10-10 % electrode layout is depicted in gray in the background. Cz served as online reference. The figure was created using the Fieldtrip[1] layout files.

**Table S1**

*Statistical description of significant clusters for cue- and target-related analyses.*

| Task | Frequency range | Time range | $t$-mass | | cluster size | | $\omega_p^2$ | | Contributing electrodes |
|---|---|---|---|---|---|---|---|---|---|
| | *Hz* | *ms* | *sum* | *p* | *#voxel* | *p* | *mean ± SD* | *95% CI* | |
| cue-related theta cluster | | | | | | | | | |
| parity/magnitude | 3.0–5.6 | 30–570 | 673.45 | .025 | 212 | .022 | .031 ±.006 | [.030, .032] | 1 2 6 7 8 9 15 25 |
| global/local | 3.0–5.6 | 290–710 | 962.90 | .007 | 281 | .007 | .036 ±.010 | [.034, .038] | 1 2 6 7 15 16 23 24 25 |
| number/letter | 3.0–6.3 | 250–910 | 682.03 | .024 | 208 | .022 | .035 ±.008 | [.033, .037] | 1 2 3 5 7 8 Cz |
| cue-related alpha/beta cluster | | | | | | | | | |
| parity/magnitude | 5.6–46.7 | 190–990 | -25922.50 | <.001 | 6028 | <.001 | .061 ±.034 | [.060, .062] | all electrodes except 18 |
| global/local | 3.0–41.3 | 290–990 | -39560.57 | <.001 | 8381 | <.001 | .073 ±.041 | [.072, .074] | all electrodes |
| number/letter | 5.6–41.3 | 150–990 | -24468.30 | <.001 | 5709 | <.001 | .064 ±.033 | [.063, .065] | all electrodes except 26 |
| target-related theta cluster | | | | | | | | | |
| parity/magnitude | 3.0–8.1 | -210–490 | 1662.94 | .006 | 475 | .006 | .039 ±.014 | [.037, .040] | 7 8 17 18 19 26 27 28 |
| number/letter | 3.0–6.3 | -350–150 | 1557.07 | .003 | 471 | .002 | .036 ±.008 | [.035, .037] | 1 2 3 4 6 7 8 9 15 17 23 24 26 27 28 Cz |
| target-related alpha/beta cluster | | | | | | | | | |
| parity/magnitude | 3.0–46.7 | -410–1490 | -44048.88 | <.001 | 11381 | <.001 | .049 ±.023 | [.048, .050] | all electrodes |
| global/local | 3.9–46.7 | -430–1490 | -54464.53 | <.001 | 12845 | <.001 | .059 ±.036 | [.058, .060] | all electrodes |
| number/letter | 3.4–53.0 | -390–1490 | -50437.00 | <.001 | 12881 | <.001 | .053 ±.024 | [.052, .054] | all electrodes |
| target-related beta cluster | | | | | | | | | |
| parity/magnitude | 6.3–28.4 | 750–1470 | -917.95 | .024 | 234 | .040 | .050 ±.023 | [.047, .053] | 2 8 |
| number/letter | 7.2–32.1 | 670–1490 | -1302.34 | .005 | 395 | .005 | .036 ±.008 | [.035, .037] | 2 7 8 |
| additional non-generalizable clusters | | | | | | | | | |
| number/letter (cue-related) | 3.0–5.6 | 350–810 | 791.81 | .014 | 222 | .017 | .043 ±.014 | [.041, .045] | 17 18 26 27 |
| number/letter (target-related) | 3.0–4.9 | 450–930 | -728.19 | .032 | 195 | .046 | .047 ±.016 | [.044, .050] | 23 24 31 32 |
| global/local (target-related) | 4.9–17.2 | -1870– -1370 | -2090.94 | .001 | 623 | .001 | .035 ±.009 | [.034, .036] | 3 4 6 9 10 11 12 13 14 15 18 19 20 21 22 24 25 26 27 28 29 30 31 32 |

*Note.* The significance level for univariate $t$-tests, defining whether a voxel is significant or not, was set to $p = .005$ according to Benjamin *et al.*[2]. Included cases were $n = 142$ for parity/magnitude task, $n = 144$ for global/local task, and $n = 134$ for number/letter task.

**Table S2**

*This table shows the correlations between the manifest variables separately for both conditions (switch and repeat) with their corresponding reliabilities on the diagonal.*

| | Measure | 1 | 2 | 3 | 4 | 5 | 6 | 7 | 8 | 9 | 10 | 11 | 12 | 13 | 14 | 15 | 16 | 17 | 18 | 19 | 20 | 21 | 22 | 23 | 24 | 25 | 26 |
|---|---|---|---|---|---|---|---|---|---|---|---|---|---|---|---|---|---|---|---|---|---|---|---|---|---|---|---|
| 1 | WMC - memory updating | .88 | | | | | | | | | | | | | | | | | | | | | | | | | |
| 2 | WMC - binding | .69 | .82 | | | | | | | | | | | | | | | | | | | | | | | | |
| 3 | WMC - operation span | .58 | .47 | .89 | | | | | | | | | | | | | | | | | | | | | | | |
| 4 | WMC - sentence span | .61 | .32 | .66 | .87 | | | | | | | | | | | | | | | | | | | | | | |
| 5 | BIS - processing capacity | .60 | .54 | .38 | .41 | .75 | | | | | | | | | | | | | | | | | | | | | |
| 6 | BIS - memory | .34 | .33 | .23 | .27 | .43 | .58 | | | | | | | | | | | | | | | | | | | | |
| 7 | BIS - processing speed | .42 | .44 | .20 | .22 | .50 | .43 | .49 | | | | | | | | | | | | | | | | | | | |
| 8 | BIS - creativity | .13 | .22 | .09 | .13 | .35 | .13 | .34 | .45 | | | | | | | | | | | | | | | | | | |
| 9 | cue-related theta (PM) - switch | .05 | .09 | .12 | .11 | .15 | .11 | .08 | .11 | .86 | | | | | | | | | | | | | | | | | |
| 10 | cue-related theta (PM) - repeat | .02 | .02 | .12 | .14 | .14 | .11 | .05 | .17 | .84 | .81 | | | | | | | | | | | | | | | | |
| 11 | cue-related theta (GL) - switch | .18 | .22 | .11 | .14 | .16 | .10 | .21 | -.08 | .31 | .29 | .79 | | | | | | | | | | | | | | | |
| 12 | cue-related theta (GL) - repeat | .20 | .21 | .14 | .11 | .11 | .09 | .12 | -.13 | .33 | .33 | .78 | .82 | | | | | | | | | | | | | | |
| 13 | cue-related theta (NL) - switch | .09 | .20 | .00 | .09 | .13 | .06 | .12 | -.03 | .35 | .29 | .39 | .42 | .78 | | | | | | | | | | | | | |
| 14 | cue-related theta (NL) - repeat | .07 | .23 | .01 | .07 | .13 | .02 | .08 | .01 | .32 | .29 | .35 | .35 | .70 | .65 | | | | | | | | | | | | |
| 15 | cue-related alpha/beta (PM) - switch | -.19 | -.14 | -.11 | -.12 | -.11 | -.16 | -.12 | -.20 | .12 | .03 | .17 | .02 | .14 | .08 | .97 | | | | | | | | | | | |
| 16 | cue-related alpha/beta (PM) - repeat | -.18 | -.14 | -.15 | -.15 | -.11 | -.15 | -.09 | -.14 | .09 | .03 | .19 | .05 | .13 | .05 | .93 | .96 | | | | | | | | | | |
| 17 | cue-related alpha/beta (GL) - switch | -.14 | -.16 | -.02 | -.07 | -.04 | -.22 | -.19 | -.18 | .09 | .05 | .15 | .03 | .08 | -.04 | .70 | .67 | .96 | | | | | | | | | |
| 18 | cue-related alpha/beta (GL) - repeat | -.17 | -.19 | -.05 | -.11 | -.04 | -.22 | -.12 | -.19 | .05 | .02 | .15 | .06 | .01 | -.15 | .64 | .68 | .94 | .96 | | | | | | | | |
| 19 | cue-related alpha/beta (NL) - switch | -.16 | -.13 | -.02 | -.05 | -.08 | -.04 | -.11 | -.17 | .27 | .17 | .19 | .05 | .21 | .13 | .77 | .72 | .60 | .53 | .96 | | | | | | | |
| 20 | cue-related alpha/beta (NL) - repeat | -.12 | -.14 | -.05 | -.01 | -.02 | .00 | -.05 | -.11 | .23 | .16 | .20 | .07 | .23 | .11 | .68 | .72 | .59 | .57 | .91 | .95 | | | | | | |
| 21 | target-related alpha/beta (PM) - switch | -.30 | -.25 | -.08 | -.12 | -.15 | -.17 | -.15 | -.25 | .12 | .04 | .11 | .00 | .08 | .05 | .76 | .66 | .59 | .56 | .54 | .46 | .97 | | | | | |
| 22 | target-related alpha/beta (PM) - repeat | -.31 | -.29 | -.13 | -.16 | -.17 | -.17 | -.12 | -.19 | .10 | .04 | .13 | .01 | .06 | .03 | .69 | .71 | .57 | .59 | .50 | .50 | .95 | .96 | | | | |
| 23 | target-related alpha/beta (GL) - switch | -.15 | -.18 | -.07 | -.10 | -.10 | -.27 | -.19 | -.06 | -.01 | -.04 | -.01 | -.09 | -.04 | -.06 | .40 | .42 | .78 | .75 | .36 | .39 | .55 | .59 | .98 | | | |
| 24 | target-related alpha/beta (GL) - repeat | -.16 | -.21 | -.09 | -.11 | -.09 | -.27 | -.13 | -.07 | -.04 | -.05 | .01 | -.06 | -.09 | -.13 | .31 | .37 | .68 | .75 | .27 | .34 | .50 | .57 | .95 | .97 | | |
| 25 | target-related alpha/beta (NL) - switch | -.20 | -.15 | .06 | -.11 | -.10 | -.10 | -.14 | -.24 | .15 | .11 | .04 | -.04 | .07 | .05 | .59 | .48 | .50 | .45 | .69 | .57 | .73 | .66 | .48 | .41 | .97 | |
| 26 | target-related alpha/beta (NL) - repeat | -.18 | -.17 | .01 | -.07 | -.09 | -.11 | -.12 | -.24 | .12 | .09 | .06 | -.01 | .15 | .05 | .55 | .51 | .54 | .52 | .64 | .67 | .71 | .70 | .55 | .51 | .91 | .96 |

*Note.* Correlations were calculated on $n = 139$ for memory updating and sentence span, $n = 133$ for binding, $n = 135$ for operation span, $n = 140$ for BIS, $n = 142$ for parity/magnity task, $n = 144$ for global/local task, and $n = 134$ for number/letter task. Reliabilities were estimated with Cronbach's $\alpha$ for WMC and BIS and with Spearman-Brown corrected odd-even split correlations for the electro-physiological data. Missing data was accounted for by calculating pairwise complete correlations. WMC: working memory capacity; BIS: Berlin intelligence structure test; PM: parity/magnitude task; GL: global/local task; NL: number/letter task;

**Figure S2**

*Event-related spectral perturbations in response to the target for non-generalizable clusters.*

(a) *ERSP with a focus on the target-related theta cluster.*

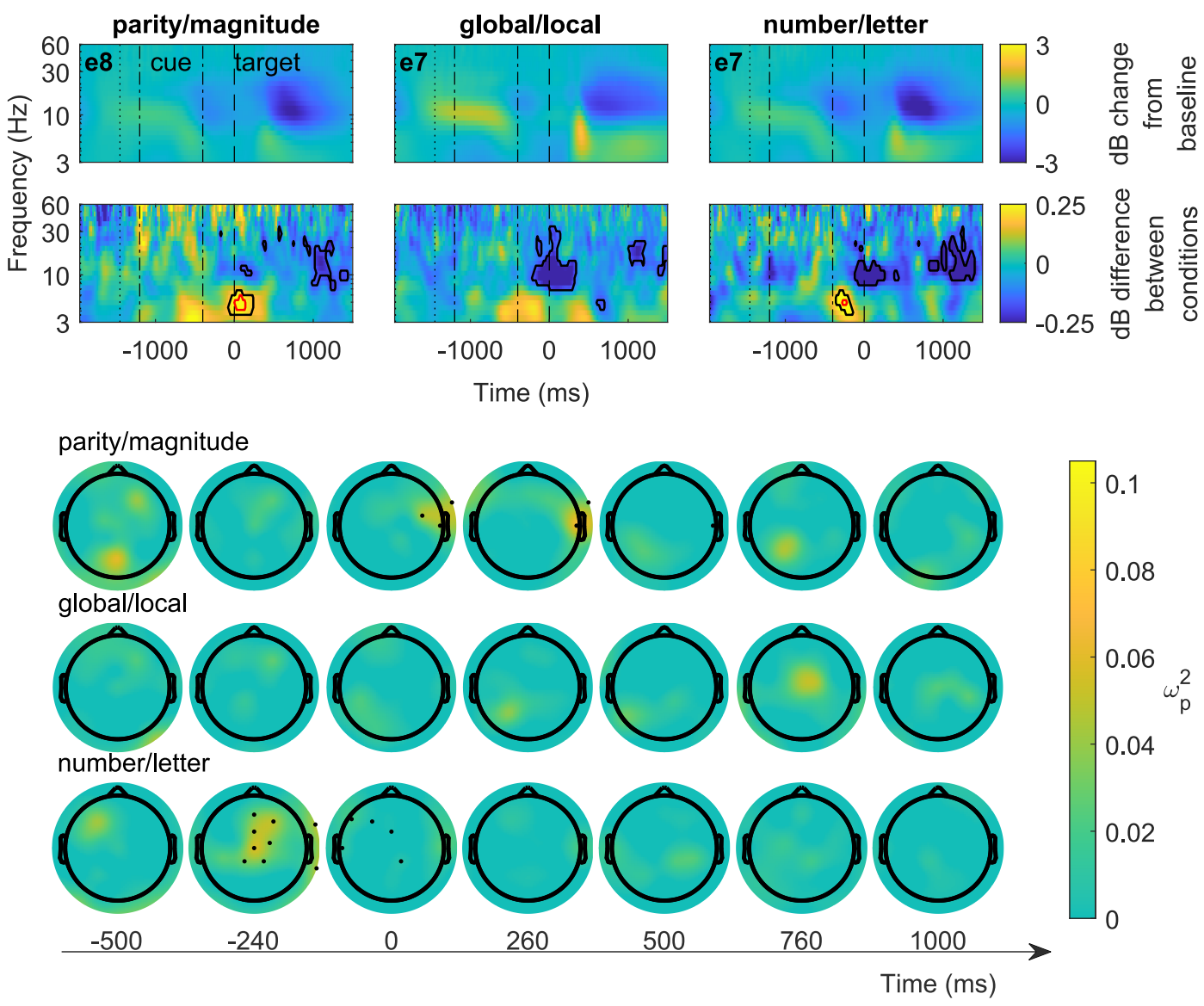


(b) *ERSP with a focus on the target-related beta cluster.*

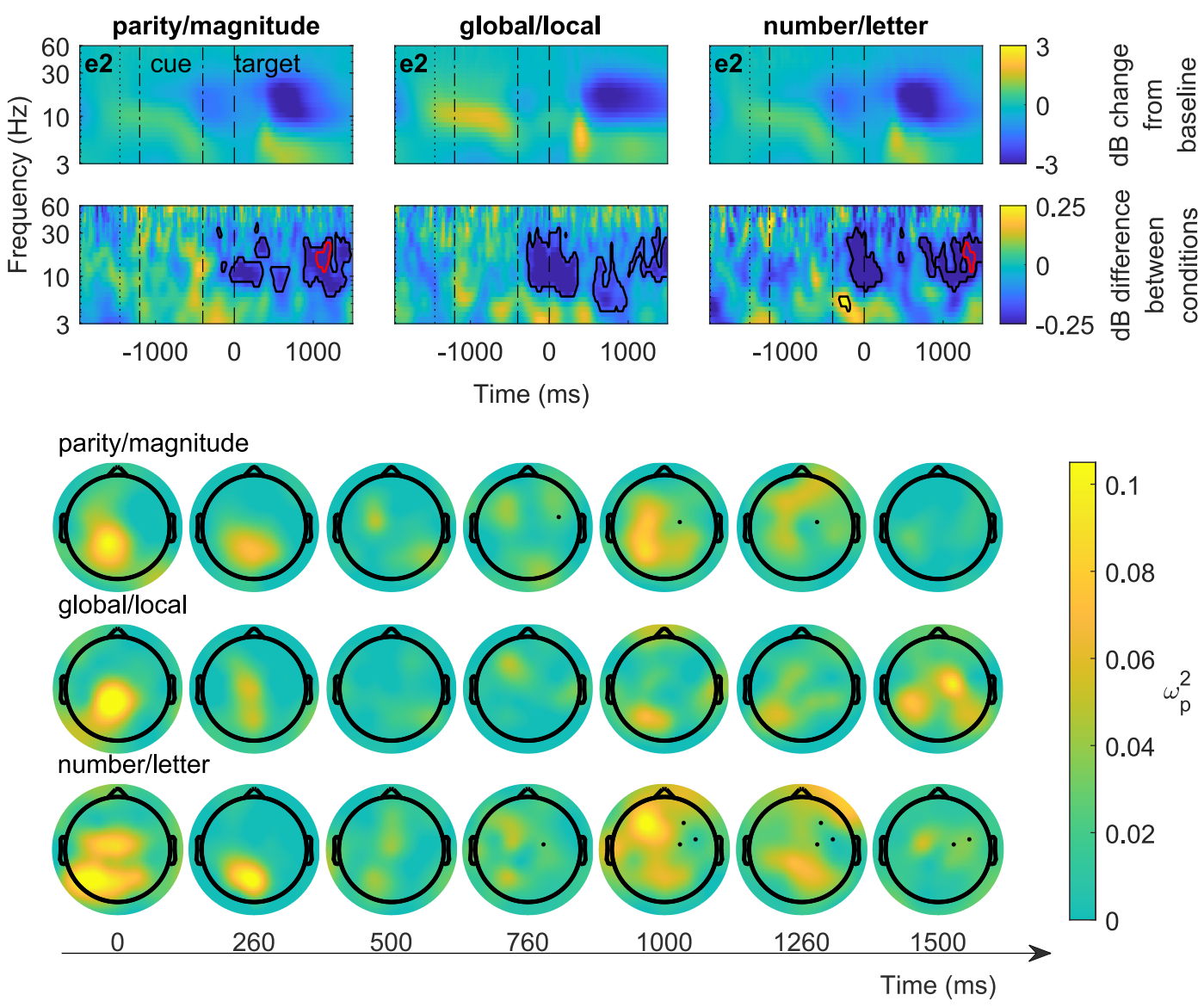


*Note.* The first rows of (a) and (b) show the dB change of power in relation to the baseline (-1950 to -1450 ms relative to target onset) averaged across both conditions. The dashed-dotted lines mark the period during which the cue was presented. The name of the depicted electrode is shown in the top-left corner. The second rows display the dB difference between the conditions (switch minus repeat) at the same electrodes. Positive values represent greater power in switch compared to repeat trials, while negative values represent lesser power. The black border surrounds voxels belonging to a cluster, and the red border indicates voxels belonging to the core region of that cluster. The lower part of (a) and (b) shows the topographic distribution of effect sizes $\omega_p^2$, derived from univariate tests contrasting the two conditions, at an exemplary frequencies of (a) 5 Hz for the theta cluster and (b) 20 Hz for the beta cluster. Additionally, electrodes contributing to the respective clusters are depicted. Included cases were $n = 142$ for parity/magnitude task, $n = 144$ for global/local task, and $n = 134$ for number/letter task.

**Table S3**

*This table shows the correlations between the latent variables of the structural equation model to test the associations between cognitive flexibility, intelligence, and working memory capacity (cf. Figure 4 in the main manuscript).*

| | Measure | 2 | | | 3 | | | 4 | | | 5 | | | 6 | | |
|---|---|---|---|---|---|---|---|---|---|---|---|---|---|---|---|---|
| 1 | cue-related flexibility | .98 | [ .95, 1.00] | < .001 | -.09 | [ -.32, .15] | .479 | -.01 | [ -.23, .21] | .925 | -.11 | [ -.35, .12] | .327 | .05 | [ -.17, .27] | .630 |
| 2 | target-related flexibility | | | | -.09 | [ -.30, .13] | .431 | -.15 | [ -.36, .07] | .193 | -.02 | [ -.25, .21] | .849 | .16 | [ -.06, .37] | .152 |
| 3 | cue-related repeat | | | | | | | .73 | [ .65, .82] | < .001 | -.17 | [ -.36, .02] | .088 | -.23 | [ -.40, -.04] | .016 |
| 4 | target-related repeat | | | | | | | | | | -.23 | [ -.42, -.04] | .020 | -.36 | [ -.53, -.20] | < .001 |
| 5 | g | | | | | | | | | | | | | .78 | [ .66, .89] | < .001 |
| 6 | WMC | | | | | | | | | | | | | | | |

*Note.* Latent correlations were calculated on $N = 147$ participants and we used full information maximum likelihood (FIML) to account for missing data in the structural equation model. g: general cognitive abilities; WMC: working memory capacity;

## Figure S3

*Distribution of the parameters non-decision time $t_0$ and drift rate $v$ from the drift-diffusion model.*

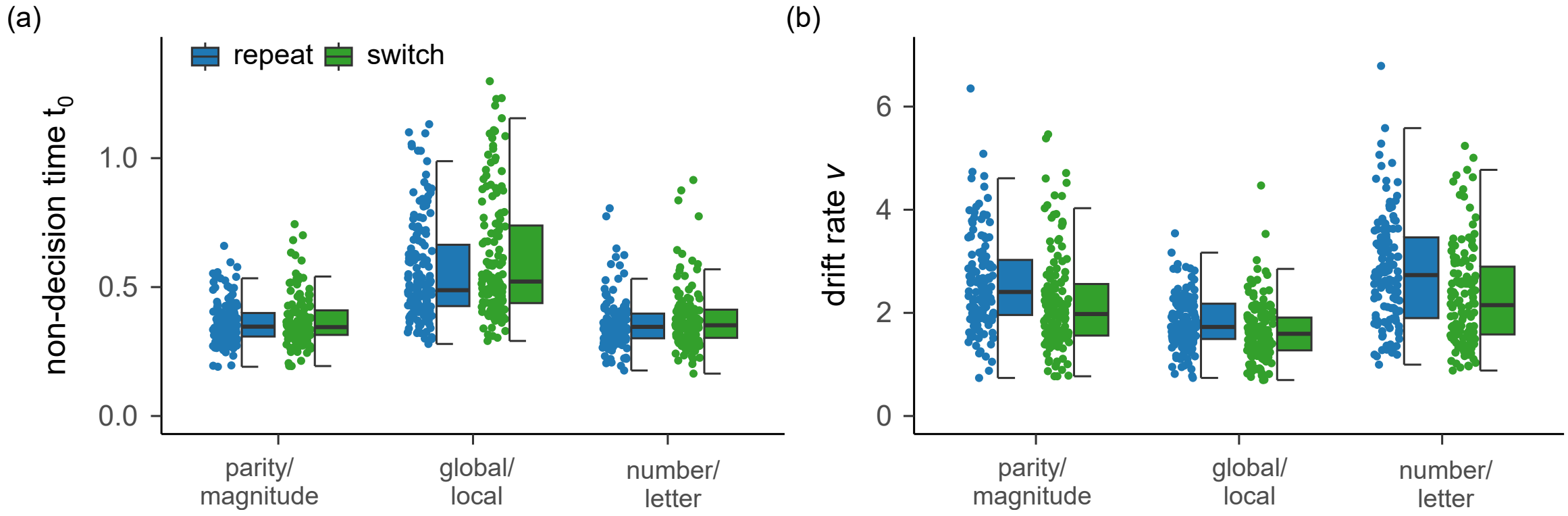


*Note.* Raw data and boxplots of (a) non-decision time $t_0$ and (b) drift rate $v$ for all three shifting tasks for both conditions are depicted. The boxplot shows the mean (center line), upper and lower quartiles (box limits), and the 95 % confidence interval (whiskers). Sample size is $n = 138$ for parity/magnitude task, $n = 144$ for global/local task, and $n = 129$ for number/letter task.

## Table S4

*This table shows the correlations between the manifest variables separately for both conditions (switch and repeat) with the corresponding reliabilities for the drift rate $v$ and non-decision time $t_0$ on the diagonal.*

| | Measure | 1 | 2 | 3 | 4 | 5 | 6 | 7 | 8 | 9 | 10 | 11 | 12 |
|---|---|---|---|---|---|---|---|---|---|---|---|---|---|
| 1 | drift rate $v$ (PM) - switch | .94 | | | | | | | | | | | |
| 2 | drift rate $v$ (PM) - repeat | .87 | .92 | | | | | | | | | | |
| 3 | drift rate $v$ (GL) - switch | .53 | .52 | .88 | | | | | | | | | |
| 4 | drift rate $v$ (GL) - repeat | .61 | .62 | .80 | .84 | | | | | | | | |
| 5 | drift rate $v$ (NL) - switch | .64 | .59 | .39 | .49 | .90 | | | | | | | |
| 6 | drift rate $v$ (NL) - repeat | .57 | .63 | .33 | .48 | .83 | .88 | | | | | | |
| 7 | non-decision time $t_0$ (PM) - switch | -.14 | -.16 | .01 | -.09 | -.14 | -.10 | .91 | | | | | |
| 8 | non-decision time $t_0$ (PM) - repeat | -.04 | -.03 | .06 | -.03 | -.08 | -.09 | .90 | .89 | | | | |
| 9 | non-decision time $t_0$ (GL) - switch | -.31 | -.34 | .00 | -.24 | -.19 | -.26 | .46 | .43 | .96 | | | |
| 10 | non-decision time $t_0$ (GL) - repeat | -.26 | -.29 | .04 | -.15 | -.15 | -.24 | .42 | .42 | .95 | .95 | | |
| 11 | non-decision time $t_0$ (NL) - switch | -.02 | .00 | .11 | .03 | -.02 | -.03 | .46 | .49 | .36 | .32 | .92 | |
| 12 | non-decision time $t_0$ (NL) - repeat | .02 | .03 | .10 | .04 | .09 | .10 | .47 | .52 | .40 | .40 | .84 | .91 |
| 13 | cue-related alpha/beta (PM) - switch | -.17 | -.20 | -.11 | -.15 | -.14 | -.10 | .08 | .00 | .10 | .08 | .17 | .22 |
| 14 | cue-related alpha/beta (PM) - repeat | -.13 | -.12 | -.03 | -.09 | -.10 | -.02 | -.02 | -.09 | .10 | .10 | .13 | .18 |
| 15 | cue-related alpha/beta (GL) - switch | -.24 | -.21 | .02 | -.05 | -.20 | -.11 | .21 | .11 | .23 | .18 | .29 | .31 |
| 16 | cue-related alpha/beta (GL) - repeat | -.31 | -.27 | .02 | -.07 | -.24 | -.12 | .17 | .10 | .18 | .15 | .25 | .27 |
| 17 | cue-related alpha/beta (NL) - switch | -.02 | -.06 | .06 | -.09 | -.13 | -.09 | .01 | -.02 | .07 | .06 | .24 | .24 |
| 18 | cue-related alpha/beta (NL) - repeat | -.02 | -.05 | .14 | -.05 | -.07 | -.01 | -.04 | -.08 | .06 | .05 | .18 | .20 |
| 19 | target-related alpha/beta (PM) - switch | -.33 | -.39 | -.14 | -.19 | -.17 | -.16 | .16 | .09 | .24 | .22 | .22 | .26 |
| 20 | target-related alpha/beta (PM) - repeat | -.31 | -.35 | -.07 | -.15 | -.13 | -.10 | .09 | .03 | .27 | .25 | .19 | .24 |
| 21 | target-related alpha/beta (GL) - switch | -.29 | -.27 | .02 | -.15 | -.19 | -.15 | .24 | .17 | .40 | .33 | .28 | .33 |
| 22 | target-related alpha/beta (GL) - repeat | -.33 | -.30 | .00 | -.18 | -.18 | -.15 | .18 | .14 | .35 | .31 | .23 | .28 |
| 23 | target-related alpha/beta (NL) - switch | -.21 | -.31 | -.12 | -.27 | -.34 | -.39 | .10 | .05 | .17 | .13 | .28 | .24 |
| 24 | target-related alpha/beta (NL) - repeat | -.21 | -.25 | -.02 | -.22 | -.25 | -.28 | .08 | .01 | .19 | .15 | .25 | .25 |

*Note.* Full correlations for all electrophysiological data can be found in Table S3. Correlations were calculated on $n = 138$ for parity/magnitude task, $n = 144$ for global/local task, and $n = 129$ for number/letter task. Reliabilities were estimated with Spearman-Brown corrected odd-even split correlations and are shown on the diagonal axis in columns 1 to 12. Missing data was accounted for by calculating pairwise complete correlations. PM: parity/magnitude task; GL: global/local task; NL: number/letter task;

**Table S5**

*This table shows the freely estimated correlations between the latent variables of the structural equation model to test the associations between behaviorally-measured and electrophysiologically-measured cognitive flexibility.*

| | Measure | 2 | | | 3 | | | 4 | | | 5 | | | 6 | | | 7 | | | 8 | | |
|---|---|---|---|---|---|---|---|---|---|---|---|---|---|---|---|---|---|---|---|---|---|---|
| | | $r$ | 95% CI | $p$ | $r$ | 95% CI | $p$ | $r$ | 95% CI | $p$ | $r$ | 95% CI | $p$ | $r$ | 95% CI | $p$ | $r$ | 95% CI | $p$ | $r$ | 95% CI | $p$ |
| 1 | cue-related flexibility | .99 | [.96,1.00] | $<$ .001 | .25 | [.02,.49] | .040 | .15 | [-.10,.40] | .227 | -.02 | [-.10,.07] | .694 | | | | | | | | | |
| 2 | target-related flexibility | | | | .17 | [-.07,.41] | .152 | .17 | [-.07,.42] | .171 | | | | -.09 | [-.17,-.01] | .030 | | | | | | |
| 3 | $t_0$-related flexibility | | | | | | | .08 | [-.20,.36] | .568 | | | | | | | -.42 | [-.76,-.08] | .060 | | | |
| 4 | $v$-related flexibility | | | | | | | | | | | | | | | | | | | -.22 | [-.44,-.00] | .053 |
| 5 | cue-related repeat | | | | | | | | | | | | | .74 | [.66,.82] | $<$ .001 | -.01 | [-.18,.15] | .878 | -.21 | [-.38,-.05] | .016 |
| 6 | target-related repeat | | | | | | | | | | | | | | | | .08 | [-.08,.24] | .337 | -.39 | [-.53,-.24] | $<$ .001 |
| 7 | $t_0$-related repeat | | | | | | | | | | | | | | | | | | | -.12 | [-.29,.05] | .162 |
| 8 | $v$-related repeat | | | | | | | | | | | | | | | | | | | | | |

*Note.* Latent correlations were calculated on $N = 147$ participants and we used full information maximum likelihood (FIML) to account for missing data in the structural equation model.

## Supplementary References

1. Oostenveld, R., Fries, P., Maris, E. & Schoffelen, J.-M. FieldTrip: Open Source Software for Advanced Analysis of MEG, EEG, and Invasive Electrophysiological Data. *Computational Intelligence and Neuroscience* **2011,** 156869. doi:10.1155/2011/156869 (2011).
2. Benjamin, D. J. *et al.* Redefine Statistical Significance. *Nature Human Behaviour* **2,** 6–10. doi:10.1038/s41562-017-0189-z (2018).
